# Molecular Scale Hydrophobicity and Adsorption Thermodynamics on Hydrophobic-Charged Surfaces

**DOI:** 10.1021/acsnano.5c18643

**Published:** 2026-02-16

**Authors:** Md Jakir Hossen, Adel Nematipour, Camille Bilodeau

**Affiliations:** † Department of Chemical Engineering, 2358 University of Virginia, Charlottesville, Virginia 22903-1738, United States

**Keywords:** hydrophobicity, chemical patterning, self-assembled monolayers, molecular dynamics, enhanced sampling, functionalized gold nanoparticle, adsorption

## Abstract

Molecular-scale hydrophobicity, which governs many important phenomena, such as aggregation, repulsion, or separation of molecules, is determined largely by the chemical composition of the functional groups exposed near the surface-water interface. However, the contributions of these groups to water-mediated interactions are nonadditive, making it challenging to understand how chemical patterning influences hydrophobicity. To address this challenge, we examined a series of model alkanethiol self-assembled monolayers (SAMs) functionalized with 1) nonpolar methyl head groups and 2) polar (hydroxyl) and positively charged (guanidinium and ammonium) head groups separated at short, intermediate, and large spacings. Using molecular dynamics (MD) simulations and enhanced sampling tools, we quantified hydrogen bonding and ordering of local hydration water molecules as a function of the hydrophilic group spacing and hydrophilic group type. Additionally, we quantified the dewetting thermodynamics of interfacial water near patterned surfaces, along with the binding strength of two model hydrophobic solutes: an alkanethiol-functionalized gold nanoparticle (GNP) and a hydrophobic protein, hydrophobin. We found that the interface dewets less readily near charged groups compared with uncharged hydrophilic groups due to their tendency to impede cavity growth at the interface. We also found that different positively charged groups influence hydrophobicity in different ways due to variations in the geometry, partial charge distribution, and local hydrogen bonding network. Furthermore, the spacing between charged groups plays a major role in modulating hydrophobicity, with specific ‘sweet spot’ distances maximizing hydrophilicity. This work conceptually bridges dewetting and adsorption thermodynamics, elucidating how surface chemistry and patterning govern hydrophobic behavior.

## Graphical abstract

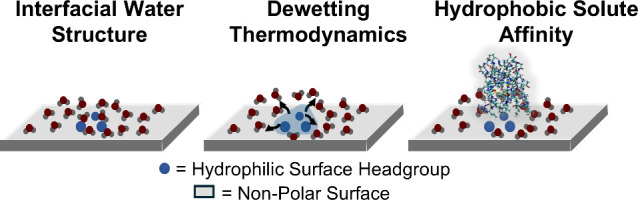

Surface hydrophobicity can be defined as the tendency of nonpolar molecules to be attracted to each other in water due to their lack of favorable interactions with water. This molecular scale repulsion of water governs a range of phenomena both at natural and engineered surfaces such as selective adsorption or permeation at biological interfaces,^1–3^ protein-protein interactions,^4–6^ fouling and antifouling phenomena,^7–9^ and reactant adsorption in catalytic systems.^10–12^ Notably, on molecular length scales these surfaces are highly heterogeneous and contain nonpolar, polar, and charged groups in a complex arrangement. While nonpolar surfaces are conventionally thought of as hydrophobic and polar/charged surfaces as hydrophilic, the hydrophobicity of mixed nonpolar and polar/charged surfaces is difficult to predict. For instance, Abbott and co-workers investigated the hydrophobicity of mixed charged and hydrophobic surfaces and showed that coimmobilization of charged amine near nonpolar residues enhanced the hydrophobic interaction, whereas guanidinium groups eliminated the hydrophobic interaction, even though both are individually hydrophilic.^13–15^ Furthermore, multiple studies exploring the hydrophobicity of mixed nonpolar and polar surfaces revealed that hydrophobicity depends not only on the fraction of polar/nonpolar groups, but also on their arrangement and patterning.^7,16–21^ However, most of these investigations that explore hydrophilic group arrangement have been limited to uncharged surfaces with only a few focusing on charged groups.^7^ Therefore, to further understand how the charged group type (e.g., ammonium and guanidinium) and arrangement modulate hydrophobicity, it is essential to examine the molecular structure and behavior of interfacial water molecules and quantify their role in water-mediated interactions.

The simplest approach to measuring the hydrophobicity of a surface is to study its interfacial water structure. The interfacial water can be characterized either by computational approaches such as density functional theory (DFT) and molecular dynamics (MD) simulation or experimental techniques such as sum-frequency generation (SFG) spectroscopy and terahertz (THz) calorimetry.^22–27^ Many studies have shown that structural features of interfacial water, such as local density, number of hydrogen bonds, and orientational distribution, are correlated with water stability and therefore the hydrophobicity of surfaces.^28–36^ Water molecules near hydrophobic surfaces tend to be less stable (less ordered) due to the unfavorable interaction between nonpolar surface headgroups and interfacial water molecules making them easier to displace, whereas water molecules near hydrophilic surfaces are generally more ordered and therefore more stable.^37,38^ Notably, for surfaces exhibiting both charge and hydrophobicity, interfacial water molecules are more ordered near charged groups and less ordered near hydrophobic groups.^6,39,40^ In this way, characterizing the behavior of interfacial water molecules elucidates the influence of surface chemistry and patterning on hydrophobicity, providing an important link to understanding more complex phenomena such as dewetting and adsorption.

Indirect umbrella sampling (INDUS)^41,42^ has recently emerged as a widely used computational tool for directly quantifying the dewetting free energy, which is defined as the energy required to create a cavity by removing interfacial water molecules.^18,20,28,31,43–48,48,49^ The cavity is created by defining an observation volume near the interface and introducing an unfavorable bias to facilitate sampling the full dewetting process. This approach directly quantifies the thermodynamic stability of interfacial water molecules (i.e., the hydrophobicity of the surface) by measuring the free energy penalty associated with removing them from the surface. Importantly, this approach does not involve interactions with a hydrophobic probe molecule and instead focuses on measuring the contribution of the dewetting process to the hydrophobic interaction. In this way, INDUS provides a solute-agnostic approach to measuring the stability of interfacial water molecules across surfaces with varying degrees of hydrophobicity.

In contrast, multiple experimental and simulation approaches measure the hydrophobicity of surfaces by measuring how strongly they interact with a hydrophobic probe molecule in an aqueous solution. For example, by functionalizing an atomic force microscope (AFM) probe with nonpolar molecules, hydrophobicity can be quantified by measuring adhesion forces between the probe and a target surface in both water and methanol and subtracting these forces.^13,14,50,51^ Additionally, protein adsorption measurements using quartz crystal microbalance (QCM) offer valuable insights into how proteins interact with surfaces of varying degrees of hydrophobicity.^52^ Both experimental approaches are consistent with theoretical expectations that hydrophobic solutes preferentially bind to hydrophobic surfaces. Similarly, the thermodynamics of hydrophobic solute binding can be measured in molecular simulations via umbrella sampling.^53^ Umbrella sampling measures the free energy of binding by introducing a positional bias to the solute and quantifying the force imposed on the solute by the surface and solvent.^7,53,54^ Collectively, these methods provide a valuable complement to INDUS by directly measuring the strength of the hydrophobic adsorption of specific solutes, making them more applicable to real applications. For example, this approach can be systematically utilized to understand the role of hydrophobic and hydrophilic hydration water that are crucial in protein stability, phase separation and protein-ligand binding phenomena.^48,55,56^ While their solute-dependence makes them less direct indicators of intrinsic surface hydrophobicity, they play a key role in linking dewetting thermodynamics to practical phenomena.

In exploring the relationships between surface chemistry, the thermodynamics of dewetting, and hydrophobic solute adsorption, two key questions arise: first, how do the chemistry and spatial arrangement of charged or polar groups influence the structure and stability of interfacial water molecules, and second, how do these interfacial water molecules, in turn, govern the binding affinity of hydrophobic solutes? To address these questions, we began by characterizing the role of these groups in shaping the water structure and stability at the interface. We created homogeneous methyl- and hydroxyl-terminated self-assembled monolayers (SAMs) as hydrophobic and hydrophilic reference surfaces as well as surfaces containing hydroxyl, guanidinium, and ammonium terminated strands positioned within a hydrophobic methyl terminated SAM at varying separation distances. Then, we utilized unbiased molecular dynamics (MD) simulations to characterize the interfacial water structure and biased indirect umbrella sampling (INDUS) simulations to quantify the dewetting free energy at various interfaces. Finally, to investigate the thermodynamics of hydrophobic solute adsorption, we utilized spatial umbrella sampling simulations and compared the binding of two hydrophobic solutes, a homogeneous alkanethiol functionalized gold nanoparticle (GNP) and a model hydrophobic protein, hydrophobin, which is comparatively heterogeneous (containing a combination of polar and nonpolar groups even at its hydrophobic interface). We also examined the behavior of hydration waters that remain at the interface of the hydrophobic solutes and the surface, which play a key role in governing the overall solute binding strength. Collectively, our results shed light on how the arrangement of charged and hydrophobic groups governs the hydrophobicity.

## Results and Discussion

### Behavior of Hydration Water Molecules near Polar/Charged-Hydrophobic Surfaces

We first examined the interfacial water structure near surfaces containing three hydrophilic groups, hydroxyl, ammonium, and guanidinium, at a range of spacings. To describe the spacing between hydrophilic groups, we describe each surface using the label SX where X denotes the number of SAM strands separating the hydrophilic groups (Figure 1a-c). Each additional SAM strand separates each hydrophilic group by approximately 0.5 nm. Our analysis focuses on three interfacial water properties previously linked to hydrophobicity, the average number of SAM-water hydrogen bonds, local water density, and water orientational distribution.^28,33^ Additionally, we characterized local water-water hydrogen bonding by analyzing spatial hydrogen bond density around the hydrophilic group.

**Figure 1:**
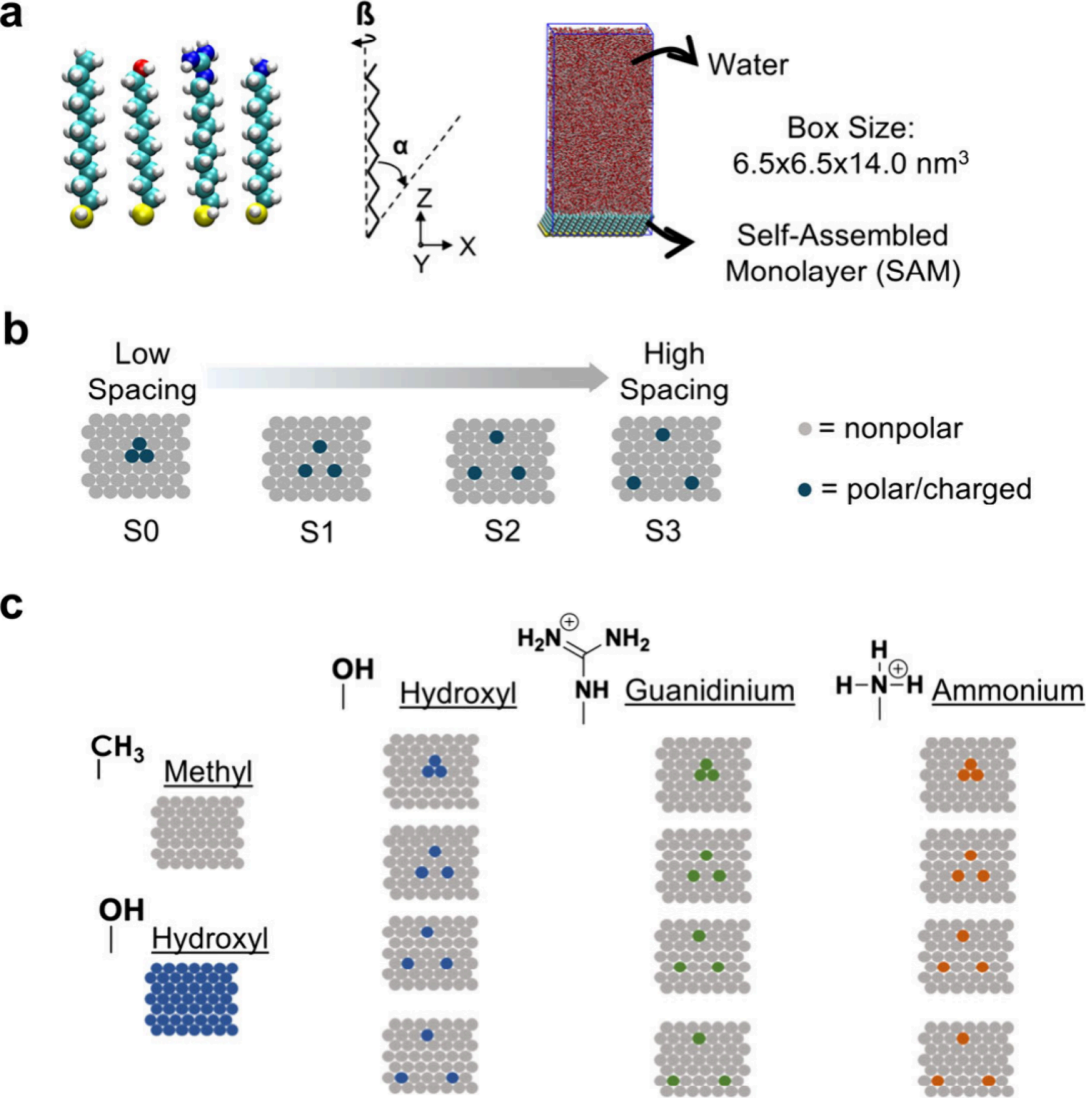
System description (a) left: Illustration of the four alkanethiol strand consisted of 10 carbon atom each and each strand functionalized with a terminal group of either nonpolar, polar, or charged (+1) group (red: oxygen, cyan: carbon, yellow: sulfur, white: hydrogen, and blue: nitrogen). Center: Schematic of the tilt (α ∼ 30°) and twist (β = 53°) applied to each SAM strand. Right: Snapshot of SAM system with a box of water (containing approximately 19,500 water molecules) added at the top of the surface which is used as the initial structure for molecular dynamics simulations. (b) Illustration of spacing between hydrophilic groups. (c) Schematic of surface functional group arrangement in SAM systems (gray: methyl, blue: hydroxyl, green: guanidinium, orange: ammonium).

We begin by measuring the number of hydrogen bonds between water and the SAM surfaces, which has been shown previously to be strongly correlated with the hydration free energy by Van Lehn and co-workers.^28^ The SAM-water hydrogen bonding showed spacing dependence, with the closest spacing (S0 ∼ 0.5 nm) resulting in a minimum for charged surfaces and a plateau at larger spacings (Figure 2a). At the closest spacing, the first hydration shells of the charged groups overlap, leading to competition among water molecules for hydrogen bonding. In contrast, at larger spacings (S1, S2, and S3, >1 nm), the first hydration shells no longer overlap and the number of hydrogen bonds plateaus, becoming independent of spacing. Among the hydrophilic groups, hydroxyl forms the fewest hydrogen bonds across all spacings because it has fewer hydrogen bond donor/acceptor sites. Interestingly, guanidinium and ammonium exhibit a similar number of hydrogen bonds at larger spacings, despite guanidinium having more hydrogen bond donor sites. We expect that this is due to the planar geometry of the guanidinium group, which limits it to forming a maximum of three hydrogen bonds.^30^ We note that hydrogen-bonding strength varies across systems, and the average number of SAM-water hydrogen bonds reported here corresponds to a specific donor–acceptor distance (≤0.35 nm) and angle threshold (D–H–A angle ≥ 150°) (see for trends with a slightly relaxed threshold).

**Figure 2:**
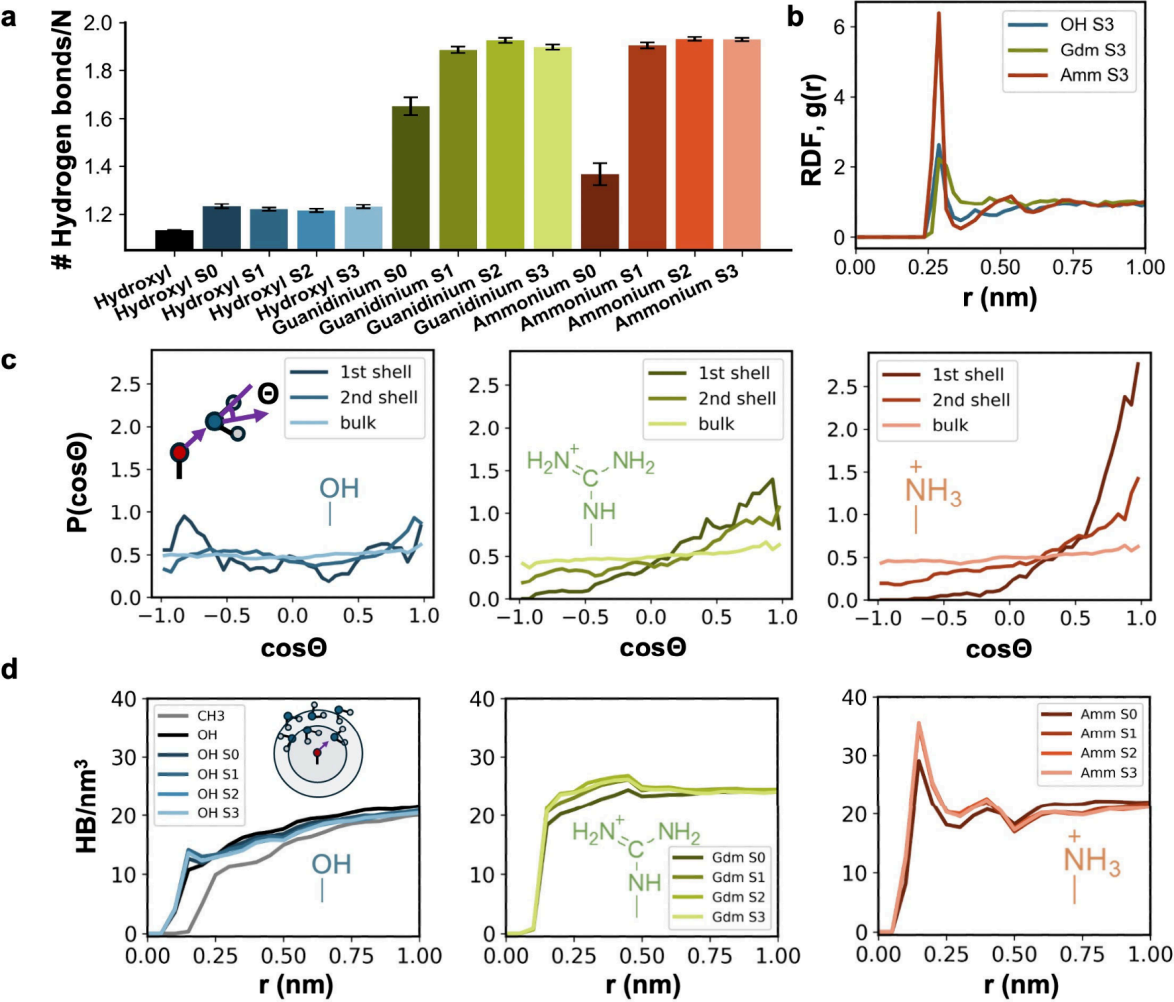
Effect of hydrophilic group type and arrangement on the structural features of interfacial water. (a) Average number of SAM–water hydrogen bonds per hydrophilic group (N denotes the number of hydrophilic groups). For hydroxyl-containing SAMs, the average number of hydrogen bonds per hydrophilic group includes both donor and acceptor interactions. Error bars represent the standard error of the mean, obtained via block averaging. (b) The pairwise radial distribution function between the oxygen of water and the oxygen in hydroxyl, nitrogen in guanidinium, and nitrogen in ammonium groups. S3 denotes the farthest spaced configuration of hydrophilic groups. (c) Probability density of the cosine of the angle between the hydrogen bond vector and the oxygen–water center of mass vector. The first hydration shell is defined as *r* < 0.4 nm, the second shell as 0.4 < *r* < 0.7 nm, and the bulk region as 1.2 < *r* < 1.5 nm. (d) Local water–water hydrogen bond density. The hydrogen bond counts correspond to hydrogen bond donor water, and the radial distance r corresponds to the center of spherical shell of 0.35 nm thick around the oxygen in hydroxyl, nitrogen in guanidinium, and nitrogen in ammonium groups. A hydrogen bond is identified using a geometric criterion: donor–acceptor (D–A) distance ≤ 0.35 nm and D–H–A angle ≥ 150°.

To investigate the water structure near the individual groups, we analyzed the radial distribution function (RDF) and angular distributions in the S3 configuration (Figure 2b-c). The RDF of ammonium (Figure 2b) shows a first peak that is roughly three times higher than the first peaks of hydroxyl and guanidinium, indicating that ammonium is more strongly hydrated than the guanidinium or hydroxyl groups, which is consistent with previous studies.^57^ We note that since guanidinium has three nitrogens, both the RDF and angular distributions are reported for one nitrogen (terminal) only. The angular distributions in the first and second hydration shells similarly illustrate that water is highly ordered near ammonium and moderately ordered near hydroxyl and guanidinium (Figure 2c). Around the hydroxyl oxygen, we observe small peaks at −1 < *cos θ* < −0.5 and 0.5 < *cos θ* < −1, which are consistent with the angles expected for hydrogen bond donor and acceptor interactions. In contrast, in the first solvation shell of the terminal nitrogen of guanidinium and the nitrogen of ammonium, only the hydrogen bond acceptor configuration of water is observed, with a significantly higher peak observed near ammonium. The higher peak indicates more strongly oriented water dipole. As expected, in the bulk region, the probability distribution is flat for all surfaces, indicating that water molecules are oriented randomly. RDF and angular distribution for the S0, S1, and S2 configurations are shown in Figures S2, S3.

Furthermore, we analyzed the water hydrogen bond density near the hydrophilic groups in each system (Figure 2d). Local hydrogen bond analysis provides a way to understand restructuring of water near interfaces.^24,36,58^ We observed pronounced differences in water-water hydrogen bond densities across surfaces of different chemistries as well as slight variations among different configurations. The differences in peak heights suggest enhanced hydrogen bonding interactions between water molecules, which also correlate with the number of water molecules present. Charged functional groups, particularly ammonium groups, showed higher hydrogen bond density peaks compared to others. Overall, the results show the influence of surface charged groups on restructuring of the local hydrogen bonding network.

### Measuring the Dewetting Free Energy of Polar/Charged-Hydrophobic Surfaces Using Indirect Umbrella Sampling (INDUS)

While measurements of interfacial water structure can form a picture of how the chemistry and arrangement of the surface impact water behavior, they are not a direct measurement of the hydrophobicity of a surface. Here, we quantitatively measured the hydrophobic effect by defining it as the free energy of cavity formation, that is, the free energy contribution to solute adsorption or aggregation associated with removing water molecules from the surface. Because the formation of large cavities in molecular simulations is highly improbable, to measure the free energy, we must utilize indirect umbrella sampling (INDUS) to increase sampling of the dewetted and partially dewetted states. Specifically, we used the sparse INDUS method,^59^ which uses a linear bias (*U*_*ϕ*_ = *ϕN*_*v*_) with a defined probe volume of water. We used an approximately triangular probe volume (Figure S4) encapsulating the hydrophilic group and then used the response subsequently to calculate the free energy of cavity formation (further detail is available in SI).

For all uncharged surfaces studied (fully methyl, fully hydroxyl, hydroxyl S0 and hydroxyl S1) at low values of *βϕ*, increasing the biasing potential leads to an increase in Gaussian water density fluctuations, resulting in a linear response (Figure 3a, left panel). The slope of this response is directly related to the compressibility of the first water shell of the surfaces, with more hydrophilic surfaces exhibiting a shallower slope (lower compressibility) and more hydrophobic surfaces exhibiting a steeper slope (higher compressibility). At *βϕ* ∼ 1–2, all uncharged surfaces exhibit a fast collective dewetting transition with the homogeneous hydroxyl SAM transitioning at a higher *βϕ* compared to the other surfaces. This fast collective dewetting is associated with the nucleation and growth of interfacial cavities within the probe volume, indicating that once a critical number of water molecules is removed from the probe volume, there is little to no barrier associated with growing the resulting cavity. In each case, a small number of water molecules remains in the probe volume after this transition due to the free energy penalty associated with removing water molecules from the probe volume boundary. Almost a few to no water molecules remain within the probe volume around *βϕ* ∼ 4 for all uncharged surfaces studied.

**Figure 3:**
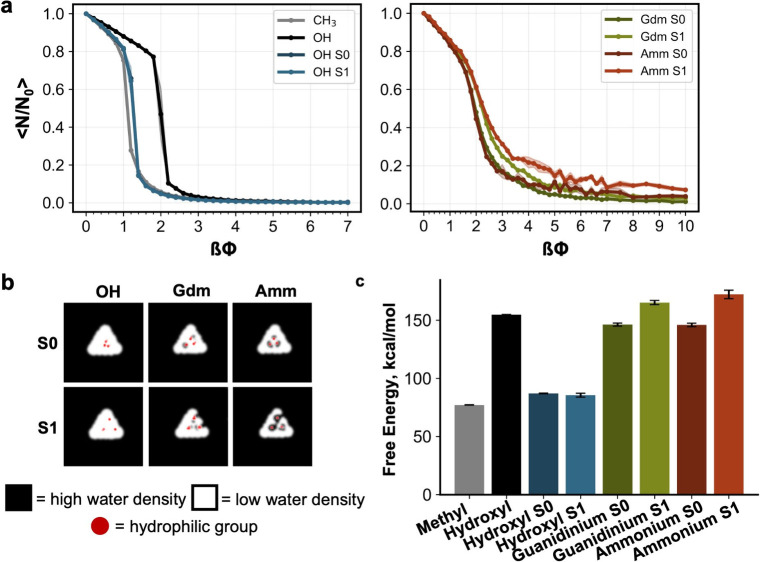
Effect of hydrophilic group type and arrangement on dewetting thermodynamics. (a) Normalized number of water molecules as a function of the applied potential. N or N_0_ denotes the average number of water molecules in the probe volume, calculated by discarding the initial 1.5 ns of a 4 ns simulation for uncharged surfaces (left panel) and the initial 3 ns of a 6 ns simulation for charged surfaces (right panel). ⟨N/N_0_⟩ represents the average from three independent simulations, with shaded error bars indicating the standard error of the mean. (b) 2D water density plot at the interface (0.6 nm, roughly 2 hydration layers) at βϕ = 7. The threshold for low water density is 5 molecules/nm^3^. (c) Free energy of cavity formation near different surfaces. The values represent the average free energy from three independent simulations, with error bars showing the standard error of the mean.

In contrast, the response of the charged surfaces studied (ammonium S0/S1 and guanidinium S0/S1, Figure 3a, right panel) to the linear biasing potential differs substantially from that of the uncharged surfaces. At low values of *βϕ*, the slope of the linear response (and therefore the compressibility of the first water shell) is nearly identical for all charged surfaces studied, matching that of the methyl surface. In this way, unlike the uncharged surfaces, compressibility is not an accurate measure of hydrophobicity, in contrast with previous studies.^16,18^ Furthermore, at intermediate values of *βϕ*, the dewetting region undergoes a slower transformation than in uncharged ones. This is because the charged groups act as “hydrophilic pins” which impede the growth of the nucleated cavities, slowing down the dewetting process. The dewetting response also suggests that S1 configurations of charged groups stabilize the water molecules more than the S0 configurations. Although it might be expected that bridging water contributes to resistance against dewetting, evidence of such water molecules was observed only for the S0 configuration across all systems, and none for the S1 configuration of ammonium S1 (Figure S5). Thus, while bridging water can effectively block hydrophobic gaps between surface polar groups, this alone cannot fully account for the observed resistance to dewetting. In addition, at a high bias potential (*βϕ* = 7), a few water molecules remain in the probe volume, indicating a high energetic penalty associated with removing those water molecules, particularly those in the first hydration shell of the ammonium groups. Figure 3b visualizes these remaining water molecules by illustrating the dewetted area. While hydroxyl S0 and S1 surfaces are fully dewetted, small regions of high-density water (illustrated in black) can be found near the charged groups on the ammonium and guanidinium surfaces.

We then calculated the dewetting free energy for each surface using the method described by Xi et al.^59^ Because it is highly energetically unfavorable to remove the final water molecules from the surfaces containing charged groups, here we defined the dewetting free energy as the energy required to remove all but the most strongly bound water molecules (those remaining at *βϕ* = 7). A higher dewetting free energy indicates a higher penalty associated with removing water molecules, reflecting a more hydrophilic surface, and a lower dewetting free energy indicates a more hydrophobic surface. As expected, the dewetting free energy is lowest for the homogeneous methyl surfaces and significantly higher near the homogeneous hydroxyl surface (Figure 3c). The S0 and S1 configurations of the hydroxyl surfaces exhibit intermediate dewetting free energy. In contrast, surfaces containing charged groups in both S0 and S1 configurations show dewetting free energies comparable to that of the homogeneous hydroxyl surface, despite a large fraction of the probe volume covering methyl groups. Notably, the S1 configurations of the charged guanidinium and ammonium groups display higher dewetting free energy as compared with S0, demonstrating that the S1 configuration of these charged groups is more hydrophilic than the S0 configuration. Importantly, these free energy trends are driven by differences in the energetics of cavity growth at intermediate values of *βϕ*, in contrast to previously studied uncharged systems, where differences in hydration free energy are driven by differences in compressibility or the potential associated with the initial onset of cavity nucleation.^47,48^ These findings are consistent with both the hydrogen bonding trends (Figure 2a) described in the previous section and previous studies that show that surface hydrophobicity is higher for spaced apart configurations of hydrophilic groups.^18,28^ Dewetting free energy for all surfaces including S2 and S3 configurations is shown in Figure S6.

Interestingly, when compared at the same spacings, ammonium and guanidinium groups do not exhibit a significant difference in dewetting free energy, as defined, despite their different effects on the interfacial water structure. This observation contrasts with previous experimental studies, which indicated that ammonium-methyl SAMs are more hydrophobic than guanidinium-methyl SAMs.^13,15^ Our simulations differ from those previous experimental studies; however, our simulations directly measure the free energy of removing interfacial water molecules, while those studies assessed the hydrophobicity by using force measurements involving hydrophobic interactions with hydrophobic probes. Therefore, in cases where the interaction with a hydrophobic probe does not lead to significant interfacial water removal as in our free energy calculation, we expect discrepancies between two hydrophobicity measurements.

### Measuring the Hydrophobicity of Polar/Charged-Hydrophobic Surfaces Using Hydrophobic Probe Solutes

To gain deeper insight into the discrepancy between INDUS and previously published experimental force measurements, we measured the free energy of binding of an idealized hydrophobic solute (obtained by equilibrating at bulk and pulling toward the surface unless otherwise specified), an alkanethiol-functionalized gold nanoparticle (GNP) (Figure 4a), to each of the mixed hydrophobic–hydrophilic SAM surfaces (Figure 4b). Additionally, we analyzed the behavior of interfacial water molecules during the interaction by measuring the dewetted area of the GNP in the most favorable bound state to investigate the fate of the hydration water of the hydrophilic groups (Figure 4c). In cases where favorable binding did not exist, we analyzed the footprint at the distance corresponding to the most favorable binding of another system.

**Figure 4:**
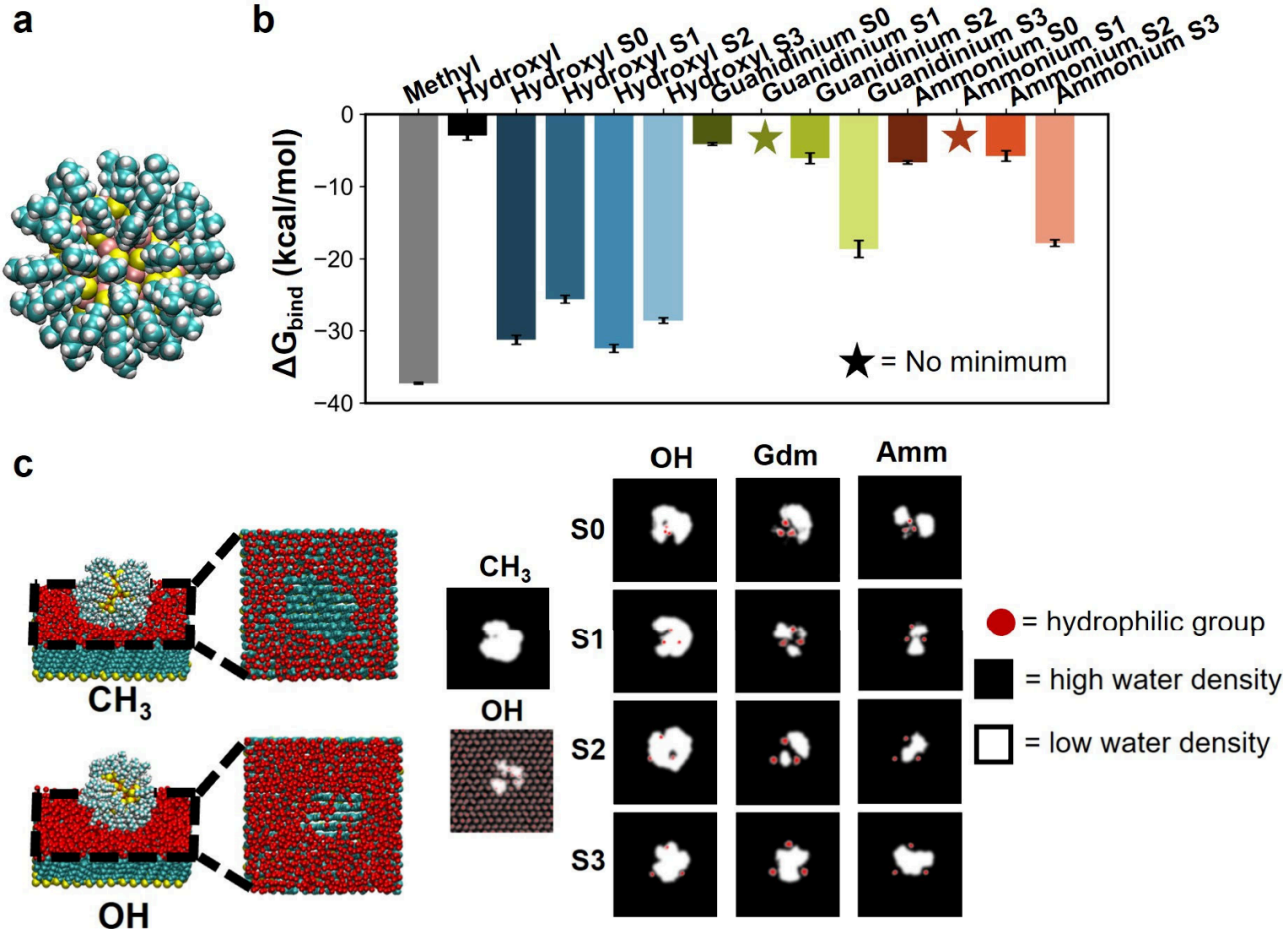
Effect of hydrophilic group type and arrangement on hydrophobic solute (GNP) binding. (a) Heptanethiol functionalized GNP (cyan: carbon, white: hydrogen, yellow: sulfur, orange: gold). (b) Free energy of GNP binding on different surfaces. The error bars represent the standard error of the mean obtained from three independent simulations. (c) Dewetted area of GNP at favorable bound state corresponding to PMF minimum. (In cases where favorable binding does not exist, we analyzed the dewetted area at distance corresponding to favorable binding of other system). The threshold for low water density is 5 molecules/nm^3^. A schematic is also shown for GNP adsorbed on homogeneous methyl and hydroxyl SAMs. Only the first two hydration shell water oxygens are shown for clarity.

### Gold Nanoparticles (GNP) Binding to Polar/Charged-Hydrophobic Surfaces

Figure 4b shows a range of binding free energies, where more negative values correspond to more favorable binding. As expected, the GNP binds most favorably to the homogeneous methyl SAM surface and significantly less favorably to the homogeneous hydroxyl SAM surface. This is expected because dewetting is significantly much more favorable on the homogeneous methyl surface than on the homogeneous hydroxyl surface, thus leading to a higher binding affinity.

GNP binding to the polar/charged-hydrophobic SAM surfaces indicates that the S1 configurations are more hydrophilic (less favorable binding) than the S0 configurations (more favorable binding), aligning with our expectations from the INDUS results. Interestingly, charged groups in the S1 configuration show more unfavorable GNP binding than the homogeneous hydroxyl SAM surface, and we do not observe any minimum in potential of mean force (PMF) (Figure S7). We note that both guanidinium S0 and ammonium S0 showed slower PMF convergence, and an extended equilibration time (40 ns for guanidinium S0 and 30 ns for ammonium S0) for these systems in particular was considered to get accurate binding free energy and subsequent analyses (Figure S8). In the S2 and S3 configurations, binding becomes more favorable, likely due to the fact that as the charged groups approach the periphery of the GNP, the GNP can form a stable interaction between the charged groups. Overall, these results illustrate a strong spacing-dependent binding affinity in charged-hydrophobic surfaces, with a clear minimum in hydrophobic binding observed for the S2 and S3 configuration.

Contrary to expectations, strongly hydrogen-bonding surfaces do not always show a reduced GNP binding affinity. For instance, the ammonium S0 SAM exhibited more favorable binding than the guanidinium S0 SAM, showing stronger agreement with previous AFM studies that suggested ammonium-methyl SAMs were more hydrophobic than guanidinium-methyl SAMs.^13,15^ However, we did not observe significant differences between ammonium and guanidinium in the binding affinity of the GNP in other configurations. This discrepancy with previous experimental studies may be due to differences in atomic resolution between our simulations and experimental setups.

Figure 4c illustrates the dewetted area of the GNP on SAM surfaces, with the dewetted regions illustrated in white and the positions of the hydrophilic groups illustrated in red. On the homogeneous hydrophobic surface (−CH_3_) the dewetting is uniform and large, while for the homogeneous hydrophilic surface (−OH) the dewetting is less continuous and small, as expected. However, the heterogeneous surfaces exhibit distinctly different dewetting behaviors. In particular, the charged groups resist dewetting more strongly than polar hydroxyl, consistent with our ‘hydrophilic pins’ observation from INDUS simulations in the previous section. Specifically, the charged groups retain nearly all hydrogen bonded water (counterion and hydrogen bonding interaction further illustrated in Figures S9–S11) upon GNP binding emphasizing the high energetics associated with removing those strongly bound water. Notably, even for hydroxyl-methyl surfaces, we did not observe complete displacement of hydrogen bonded water (Figure S9) at the favorable bound state, meaning a few water molecules dynamically form hydrogen bonds with hydroxyl groups while adsorption is still favorable.

To explore the dewetting contribution to binding affinity, we quantified the correlation between the free energy of binding and dewetted area by calculating a correlation coefficient (Figure 5). Overall, a strong correlation (Pearson’s r = -0.952) is observed between the extent of dewetting and the free energy of GNP binding, suggesting dewetting plays a central role in GNP binding. However, it is important to note that, while this correlation effectively captures the overall trend, it cannot be used to explain small differences in binding free energy. For example, while the ammonium S0 configuration exhibits a higher binding affinity compared to guanidinium S0, it does not consistently form a larger dewetted area (Figure S12).

**Figure 5:**
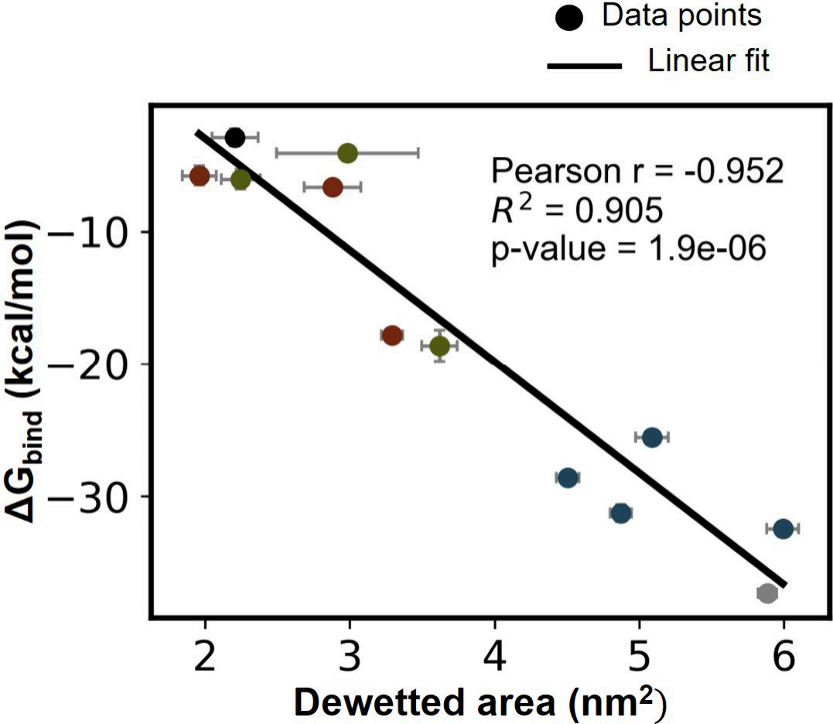
Correlation between the free energy of binding and the dewetted area in GNP binding. The data points labeled ‘Gray’, ‘Black’, ‘Blue’, ‘Green’, and ‘Red’ correspond to methyl, hydroxyl, hydroxyl–methyl, guanidinium–methyl, and ammonium–methyl SAMs, respectively.

### Hydrophobin Binding to Polar/Charged-Hydrophobic Surfaces

While the GNP binding studies in the previous section illustrate how a homogeneous idealized hydrophobic solute interacts with mixed charged/nonpolar surfaces, most real applications, such as designing drug carriers or protein separation materials, concern the adsorption of less ideal hydrophobic solutes. For example, various biomolecular interactions, such as protein-ligand binding, protein-protein interactions, and other aggregation or phase separation phenomena, are driven by complex hydration-water dynamics.^22,24,44,60^ These processes involve dewetting, direct solute-surface, solute-water interactions, and significant energetic contributions from retained water, which can serve to stabilize such interactions. To understand how these surfaces behave when interacting with a heterogeneous solute such as a protein, we extended our analysis to hydrophobin (Figure 6a) adsorption and repeated our binding studies. Notably, hydrophobin contains a large hydrophobic patch which we reoriented toward the surface during umbrella sampling. This hydrophobic patch contains mostly hydrophobic residues such that any polar interactions are driven by protein backbone atoms and a small number of polar, uncharged side chains at the periphery of the binding face. In this way, we can think of hydrophobin binding as representative of idealized hydrophobic protein binding.

**Figure 6:**
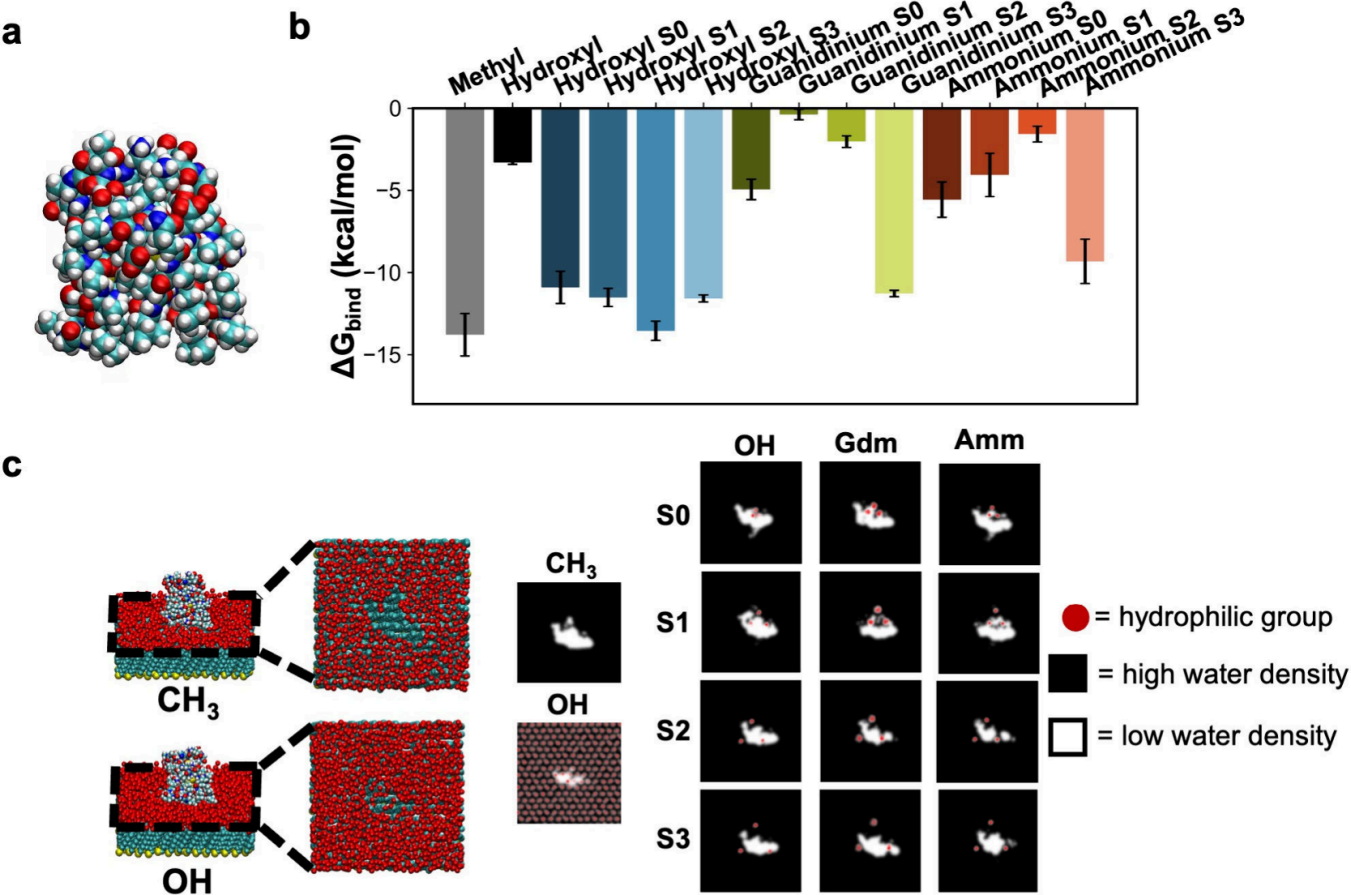
Effect of hydrophilic group type and arrangement on hydrophobic solute (hydrophobin) binding. (a) Hydrophobin (red: oxygen, cyan: carbon, yellow: sulfur, white: hydrogen, and blue: nitrogen). (b) Free energy of hydrophobin binding on different surfaces. The error bars represent the standard error of the mean obtained from three independent simulations. (c) Dewetted area of the hydrophobin at favorable bound state corresponding to PMF minimum (In cases where favorable binding does not exist, we analyzed the footprint at distance corresponding to favorable binding of other system). The threshold for low water density is 5 molecules/nm^3^. A schematic is also shown for hydrophobin adsorbed on homogeneous methyl and hydroxyl SAMs. Only first two hydration shell water oxygen is shown for clarity.

Figure 6b shows the hydrophobin binding affinity to all SAM surfaces (see Figures S13–S17 for PMF plots, counterion, and hydrogen bonding interaction). Similar to the GNP binding studies, these results suggest a stronger spacing-dependent binding affinity in charged-hydrophobic surfaces. However, there are multiple differences between GNP and hydrophobin binding. First, hydrophobin binds favorably across all surface types including charged S1 surfaces, likely due to more favorable direct interactions. This underscores the complex nature of binding, where even for a hydrophobic protein patch composed predominantly of nonpolar groups, the binding strength is dictated by more than simple hydrophobicity. Second, although the binding affinity still depends on the spacing of hydrophilic groups, the trend diverges. Specifically, the most hydrophilic configuration (S1), which consistently showed reduced GNP binding affinity, does not exhibit the same behavior for hydrophobin interacting with the ammonium-methyl surface. These differences may be attributed to either small changes in the shape of hydrophobin compared with the GNP or to its differences in surface chemistry.

To explore the dewetting behavior associated with hydrophobin adsorption, we again analyzed the areas dewetted by hydrophobin at the PMF minimum (Figure 6c). We note that the binding face of hydrophobin is expected to be less readily dewetted than the alkanethiol-functionalized GNP because of the presence of few polar groups. Additionally, the size of the dewetted region is expected to differ, as hydrophobin is smaller than GNP. Overall, our results indicate that, qualitatively, the dewetted regions are largely similar across all SAM surfaces, unlike the significantly varied dewetting observed in GNP binding, except for the reference hydrophobic and hydrophilic surfaces (Figure S18). We note that the dewetted area suggests a possible water-bridge formation between polar side chains of hydrophobin and polar or charged residues on SAM surfaces. While bridging water may contribute to the hydrophobin binding strength on homogeneous hydroxyl and ammonium S1 surfaces (Figure S19), its role in modulating binding affinity is likely less central on the S2 and S3 surfaces, which predominantly contain nonpolar groups. We expect that the influence of bridging water could become more significant in systems containing more hydrophilic groups. Overall, the results suggest a less dominant contribution of dewetting in driving the binding affinity of hydrophobin.

Finally, to quantify the dewetting contribution in hydrophobin binding, we measured the correlation between the free energy of binding and dewetted area. Overall, the correlation between dewetted areas and free energy of binding is significantly lower for hydrophobin binding compared with GNP binding (Figure 7), suggesting dewetting plays a less central role in binding of hydrophobin in polar or charged-hydrophobic SAM surfaces. Instead, direct favorable interactions between polar backbone and side chain groups (present even on the hydrophobic face of hydrophobin) likely play an important role in the favorable interactions between the SAMs and hydrophobin.

**Figure 7:**
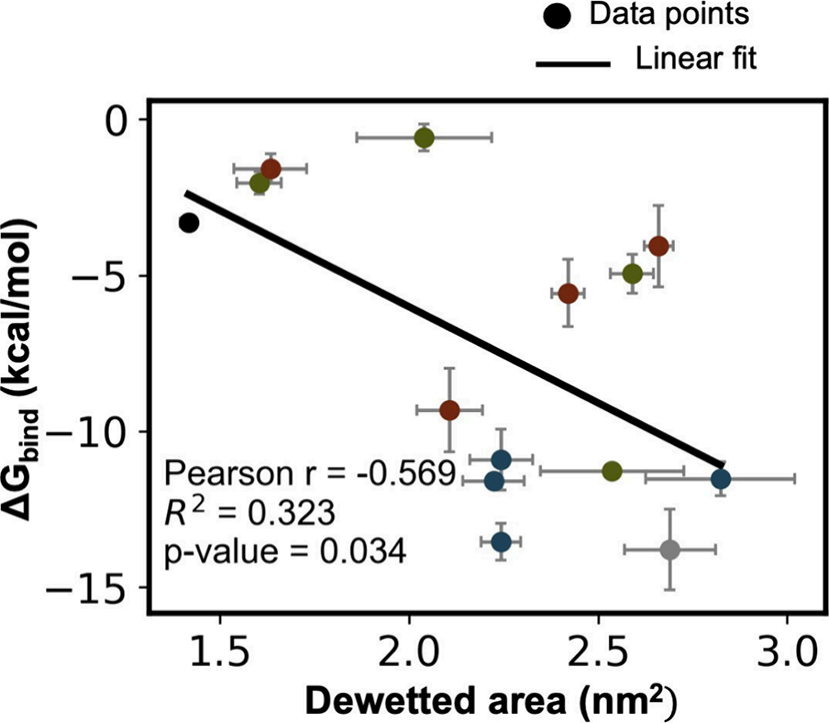
Correlation between the free energy of binding and the dewetted area in hydrophobin binding. The data points labeled ‘Gray’, ‘Black’, ‘Blue’, ‘Green’, and ‘Red’ correspond to methyl, hydroxyl, hydroxyl–methyl, guanidinium–methyl, and ammonium–methyl SAMs, respectively.

While the binding affinity and dewetting trend reported here are reported for initiating the system at the wet-unbound state, the overall trend and conclusions remain consistent for both GNP and hydrophobin adsorption when initiated from the dry-bound state, with only minor variations. A comprehensive discussion of these differences lies beyond the scope of this work; however, additional analyses comparing wet and dry initial state simulations are provided in the Supporting Information (Figures S22–S27).

## Conclusions

Charged-hydrophobic surfaces are ubiquitous in nature, and engineering and understanding the role of charged/hydrophilic group type and spacing on modulating molecular-scale hydrophobicity have remained a major challenge. Here, we characterized the molecular-scale hydrophobicity of charged-hydrophobic surfaces on three levels: (1) analyzing structural features of hydration water molecules, (2) quantifying surface dewetting thermodynamics using INDUS, and (3) evaluating hydrophobic solute binding through spatial umbrella sampling. Using self-assembled monolayers (SAMs) with varied arrangements of polar and charged groups, we investigated how molecular-scale surface chemistry modulates the interfacial water behavior and solute binding. These insights offer a mechanistic foundation for understanding and manipulating water-mediated interactions on natural and engineered surfaces.

We first analyzed the behavior of hydration waters and found that closely spaced hydrophilic groups limit hydrogen bonding due to competition among water molecules, whereas larger separations between hydrophilic groups mitigate this effect. Moreover, calculating the RDFs, orientational distributions, and local hydrogen bond density of water molecules near the various surfaces suggested that ammonium exhibits stronger hydration due to its compact structure and high charge density. Quantitative analysis from INDUS showed that dewetting is more favorable on uncharged surfaces, as expected. In contrast, charged groups resist cavity formation by impeding cavity growth at the interface, especially in the S1 configuration, which stabilizes the interfacial water. Finally, while guanidinium and ammonium display similar hydrophilicity in terms of interfacial water stability, they differ in hydrophobic solute binding affinity, as observed in umbrella sampling simulations. Specifically, we found that (1) thermodynamically stable interfacial water does not always reduce solute binding affinity (i.e., ammonium surfaces can often promote favorable binding without disrupting hydration water) and (2) while dewetting strongly correlates with binding affinity for idealized hydrophobic solutes like functionalized gold nanoparticles, this relationship weakens for realistic hydrophobic proteins such as hydrophobin, suggesting that dewetting plays a less dominant role in hydrophobin binding. We note that the hydrophobic interface of hydrophobin contains mostly hydrophobic residues, and any polar interactions are driven by protein backbone atoms and a small number of polar, uncharged side chains at the periphery of the binding face. Overall, the chemistry and spatial arrangement of polar and charged groups critically influence the interfacial water structure and significantly affect hydrophobic solute binding behavior. While we focused on hydroxyl, guanidinium, and ammonium groups only, the approach can be readily extended to a broader chemical space, including negatively charged and structurally diverse polar groups. In particular, although our results suggest that the “pinning” effect is more characteristic of charged groups than uncharged hydrophilic groups, it would be interesting to determine whether uncharged hydrophilic groups that form stronger hydrogen bonds exhibit similar behavior. Additionally, it would be valuable to explore how variations in the salt concentration influence the hydrophobicity trends observed here.

## Methods

### System Description

#### Self-Assembled Monolayers (SAMs)

We constructed and energy minimized *n*-alkanethiol strands, each comprising 10 carbon atoms and a terminal functional group, using Avogadro^61^ (Figure 1a, left). The terminal functional groups of the four strands are methyl, hydroxyl, guanidinium, and ammonium, respectively. Each strand was adjusted to the appropriate tilt and twist angles (Figure 1a, center) associated with a gold (111) surface as outlined by Love et al.^62^ The strands were then replicated in the *x*-direction, each separated by approximately 0.5 nm, and in the *y*-direction, each separated by 0.432 nm, to generate self-assembled monolayers (SAMs). Each SAM was solvated with approximately 19,500 water molecules using the OPC water model,^63^ with counterions (Cl^–^) added for charge neutrality in charged systems, to study interfacial water dynamics (Figure 1a, right). To probe the role of arrangement, we generated SAMs with varying hydrophilic group arrangement, from a low spacing of the hydrophilic group to a high spacing of the hydrophilic group (Figure 1b). Each surface is labeled as SX, where X represents the number of hydrophobic SAM strands separating adjacent hydrophilic groups, to characterize their spacing. We generated two homogeneous SAMs as reference hydrophobic and hydrophilic surfaces and 12 additional SAMs to probe the effects of both chemistry and arrangement (Figure 1c). We parametrized SAMs with the general amber force field GAFF2 ^64^ and AM1-BCC^65^ charge method. The periodic box dimensions were set to 6.5 × 6.5 × 14 nm^3^.

#### Gold Nanoparticle (GNP)

The initial structure and parameter file of the gold nanoparticle (diameter ∼ 3.5 nm) were generated using the Nanomodeler Web server.^66^ Nanomodeler employs parameters for the nonbonded internal gold atoms based on the work of Heinz et al.,^67^ and for the gold–sulfur interface, it utilizes parameters developed by Pohjolainen et al.^68^ For the alkanethiol ligands, atomic partial charges were calculated using the RESP method, while bonded parameters were assigned according to the GAFF^64^ force field.

### Hydrophobin

The PDB structure of hydrophobin, developed by Hakanpää et al.,^69^ was used. We note that only a single chain of hydrophobin (diameter ∼ 2.5 nm) dimer was used in the simulation. The corresponding parameter file was generated using the ff19SB^70^ force field.

### Simulation Details

#### Unbiased Simulation

We conducted molecular dynamics (MD) simulations in Amber (version 22)^71^ using GPU-accelerated program pmemd.cuda. The initial solvated SAM structures were energy-minimized using the steepest decent algorithm followed by the conjugate gradient algorithm. To hold each SAM strand fixed in the correct lattice position, a positional restraint of 1000 kcal/mol/nm^2^ was applied to the sulfur and seventh carbon atom of each strand. We conducted simulations for 50 ns with a 2 fs time step, maintaining constant temperature (300 K), constant pressure (1.01325 bar), and constant surface tension (10 dyn/cm) along the XY plane with semi-isotropic pressure scaling. A Langevin thermostat with a collision frequency of 3 ps^–1^ and a Berendsen barostat with a pressure relaxation time of 2 ps were used. The nonbonded cutoff was set to 0.9 nm. The SHAKE algorithm was applied to constrain bonds involving hydrogen atoms.

MDAnalysis^72^ package was utilized in python for hydrogen bond, radial distribution function (RDF) and angular distribution analysis. A hydrogen bond was considered present if the donor–acceptor distance was less than 0.35 nm and the angle between the donor, hydrogen, and acceptor atoms was less than 150°.

#### Indirect Umbrella Sampling (INDUS)

The GROMACS molecular dynamics package (version 2021.4)^73^ was modified to bias the coarse-grained water number, denoted as Ñ_v_, within the probe volume using the indirect umbrella sampling (INDUS)^42,43^ method. The Gaussian coarse-graining function employed in INDUS was configured with a standard deviation of σ = 0.01 nm and a truncation length of r_c_ = 0.02 nm. Water dynamics in the defined volume were governed by the Hamiltonian: H = H_o_ + ϕÑ_v_, where H_0_ is the unbiased Hamiltonian, and the second term represents the linear biasing potential, with ϕ as the biasing strength and Ñ_v_ as the instantaneous water number.

A triangular volume near the SAM interface (Figure S3), created by the union of spheres (R_v_ = 0.6 nm), encapsulating roughly two hydration shells near hydrophilic groups was used. Then, a linear biasing potential was employed to sample dewetting at different biasing potentials. The equilibrium number of water molecules at different biasing potentials was used to calculate free energy using the sparse sampling method.^59^ Sparse sampling returns a free energy profile as a function of ⟨Ñv⟩

$$F\left({Ñ}_{v}\right)=-\frac{1}{\beta }\;\ln P_{v}^{\phi }\left({Ñ}_{v}\right)-\phi \left\langle {Ñ}_{v}\right\rangle +\int_{0}^{\phi }\langle {Ñ}_{v}\rangle_{\phi \prime }\;d\phi^{\prime }$$

Here, *F*(Ñ_v_) denotes the unbiased free energy, β = 1/(*k*_B_ *T*) is the inverse thermal energy, and ⟨Ñ_v_⟩ is average coarse-grained number of water molecules in probe volume v, and *P*_*v*_^ϕ^ is the biased probability. The integral term is the free energy difference between biased and unbiased ensembles.

### Umbrella Sampling

Both the solvated GNP–SAM and hydrophobin–SAM systems were energy minimized using the steepest descent algorithm and subsequently equilibrated through short NPT simulations. For the production runs, positional restraints of ∼1000 kcal/mol/nm^2^ (4200 kj/mol/nm^2^) were applied in the x and y directions to keep the GNP or hydrophobin positioned above the target binding region (see Figures S20, S21 for the scatter plot showing the position of the restrained atom in GNP and hydrophobin umbrella sampling simulations). The center of mass (COM) between the GNP/hydrophobin was restrained using a harmonic umbrella potential with a pulling force constant of ∼715 kcal/mol/nm^2^ (3000 kJ/mol/nm^2^). For each system, 25–35 umbrella sampling windows were generated using steered molecular dynamics (SMD), with each window spaced 0.1 nm apart—ranging from configurations where the GNP or hydrophobin was far from the surface to those close to the surface. Both GNP and hydrophobin umbrella sampling simulations were initialized by solvation and equilibration in bulk water and then pulling them toward the surface. Each window was simulated for 100 ns. Umbrella sampling simulations were analyzed using the Weighted Histogram Analysis Method (WHAM),^74^ as implemented in GROMACS, to generate the potential of mean force (PMF). The first 2 ns of each simulation were considered equilibration unless otherwise specified and were excluded from the WHAM analysis. Error estimation of the PMF was performed using bootstrapping with 100 trials (-nBootstrap 100), as implemented in WHAM.

The free energy of binding is defined by the following equation^75^

$$\Delta G_{bind}=-RT\;\ln\left[\int_{z1}^{z2}\exp\left(-\frac{W\left(z\right)}{RT}\right)\;dz/\int_{z3}^{z4}\exp\left(-\frac{W\left(z\right)}{RT}\right)\;dz\right]$$

The potential of the mean force (PMF), denoted as *W*(*z*), describes the energy profile along the reaction coordinate. The bound region is defined between *z*_1_ and *z*_2_, selected to include the vicinity of the PMF minimum and account for thermal fluctuations up to 0.592 kcal/mol (equivalent to 1 kT). Here, *R* is the universal gas constant, *T* is the absolute temperature, and *z*_3_ and *z*_4_ define the corresponding unbound region in the bulk, where the PMF reaches a plateau. This plateau has been shifted to the *x*-axis; therefore, the denominator in the equation effectively represents the width of the bound region.

## Supplementary Material





## Data Availability

All data and scripts supporting the findings of this work are available on GitHub (https://github.com/BilodeauGroup/manuscript-data/).

## Notes

Behavior of water molecules at the interface; INDUS simulation parameters; Potential of mean force (PMF) of GNP and hydrophobin binding at SAM surfaces; Detailed analysis of hysteresis and convergence in binding affinity calculation (PDF)
Data files such as csv, and xlsx required to reproduce the results. (ZIP)

## Abbreviations

SAM: self-assembled Monolayers
MD: molecular dynamics
INDUS: indirect umbrella sampling
GNP: gold nanoparticle
QCM: quartz crystal microbalance
AFM: atomic force microscope
Amm: ammonium
Gdm: guanidinium
SMD: steered molecular dynamics
