# Supplementary material for: Molecular Scale Hydrophobicity and Adsorption Thermodynamics on Hydrophobic-Charged Surfaces
Source: ACS Nano. 2026 Feb 16;20(8):6970–82. doi: 10.1021/acsnano.5c18643 (PMC12961923; doi:10.1021/acsnano.5c18643)
Supplement: Supplementary file 1 [file nn5c18643_si_001.pdf]

Supporting Information

Molecular Scale Hydrophobicity and Adsorption Thermodynamics on Hydrophobic-Charged Surfaces

Md Jakir Hossen[1], Adel Nematipour[1], and Camille Bilodeau*[1]

[1]Department of Chemical Engineering, University of Virginia, Charlottesville, Virginia 22903-1738, United States

*Corresponding author: cur5wz@virginia.edu

Contents

1 Behavior of water molecules at the interface 3

2 INDUS: simulation parameters 4

3 Potential of Mean Force (PMF) of gold nanoparticle (GNP) binding to SAM surfaces 7

4 Potential of mean force (PMF) of hydrophobin binding to SAM surfaces 13

5 Exploring hysteresis and convergence in binding affinity calculation 22

1 Behavior of water molecules at the interface

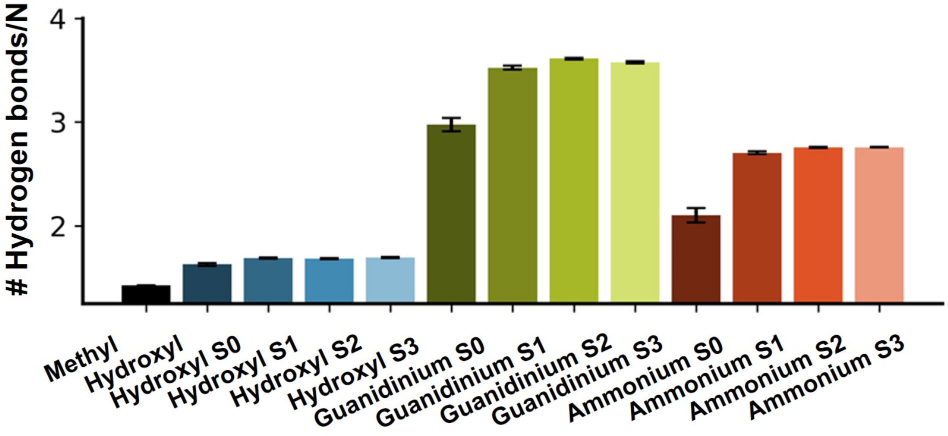

Figure S1: Average number of SAM–water hydrogen bonds per hydrophilic group (N denotes the number of hydrophilic groups). For hydroxyl-containing SAMs, the average number of hydrogen bonds per hydrophilic group includes both donor and acceptor interactions. Error bars represent the standard error of the mean, obtained via block averaging. Hydrogen bonds are identified using a geometric criterion: donor–acceptor (D–A) distance $\leq$ 0.35 nm and D–H–A angle $\geq 135°$.

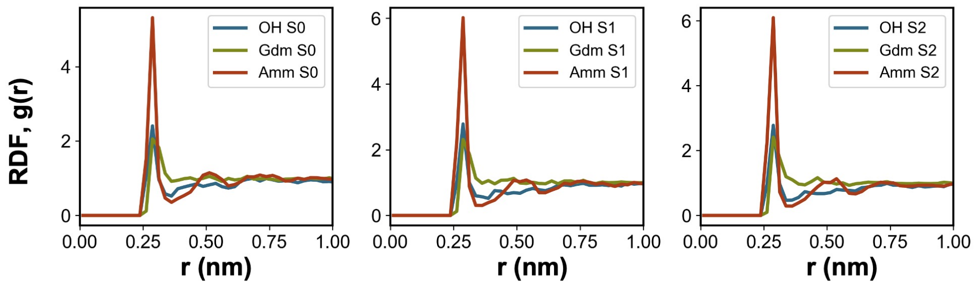

Figure S2: Effect of hydrophilic group type and arrangement on the structural features of interfacial water. The pairwise radial distribution function between the oxygen of water and the oxygen in hydroxyl, nitrogen in guanidinium and nitrogen in ammonium. S0, S1 and S3 denote the system configuration.

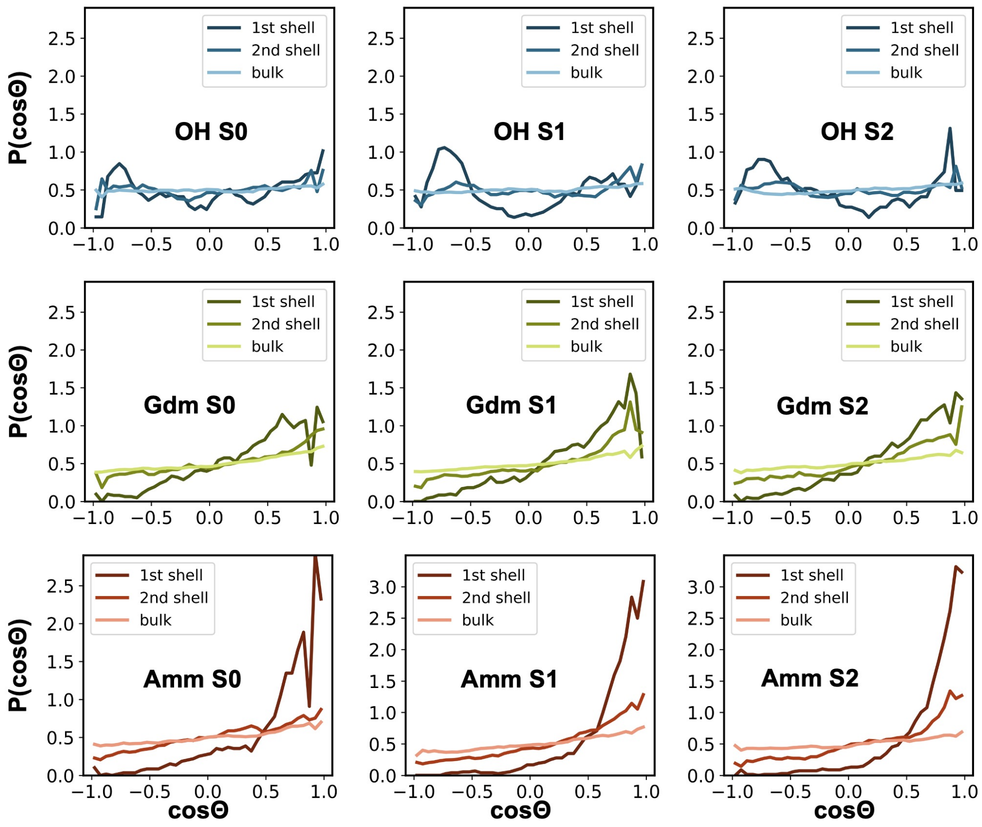

Figure S3: Effect of hydrophilic group type and arrangement on the structural features of interfacial water. Probability density of the cosine angle between the hydrogen bond vector and oxygen-water center of mass vector. The 1st hydration shell is defined as $r < 0.4$ nm, 2nd shell as $0.4 < r < 0.7$ nm and bulk as $1.2 < r < 1.5$ nm.

2 INDUS: simulation parameters

Preequilibrated structures were taken from the final frame of unbiased simulations. AMBER structure files were converted to GROMACS using ACPYPE[1] and simulations were run using the OPC water model.[2] Temperature was maintained at 300 K using the canonical velocity-rescaling thermostat,[3] and pressure at 1.01325 bar using a semi-isotropic stochastic cell-rescaling barostat.[4] Bonds involving hydrogen atoms were constrained using LINCS.[5] A strong positional restraint was applied to the sulfur atom and the seventh carbon of the SAM molecule. Weak positional restraints ($\sim$50 kJ mol^{-1} nm^{-2}) were applied to remaining atoms to preserve hydration structure.

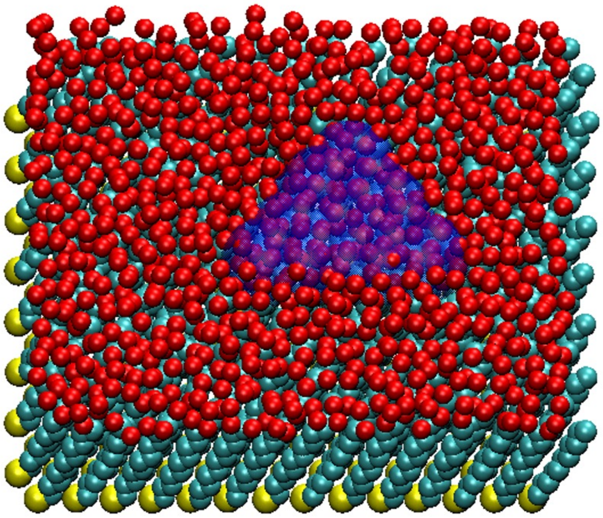

Figure S4: Illustration of the defined volume at the top of SAM surface. The schematic shows roughly two hydration layers of water oxygen at the top of methyl SAM surface. The triangular volume (shown in blue) was created by the union of spheres with a radius of 0.6 nm, with centers positioned appropriately to ensure the volume encompasses approximately two hydration layers of water molecules. red: oxygen, cyan: carbon, yellow: sulfur (hydrogen atoms are not shown for clarity)

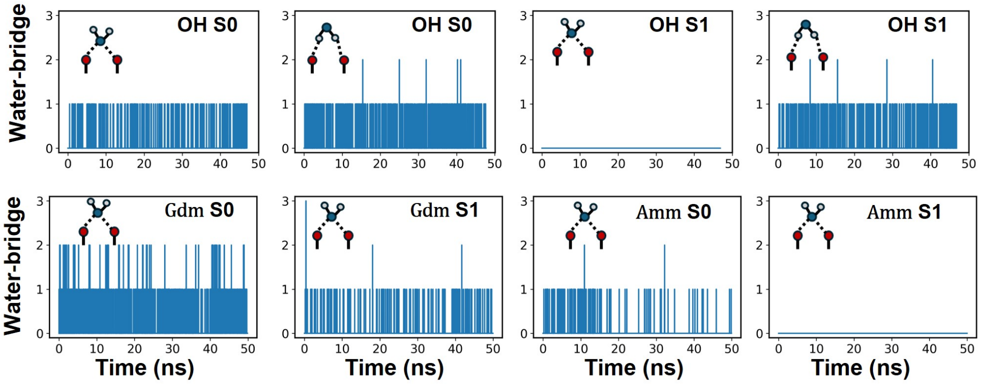

Figure S5: Water-bridge counts between hydrophilic surface groups. A water bridge is defined as a single water molecule that simultaneously forms hydrogen bonds with multiple hydrophilic groups on the surface. Hydrogen bonds are identified using a geometric criterion: donor–acceptor (D–A) distance $\leq$ 0.35 nm and D–H–A angle $\geq 150°$.

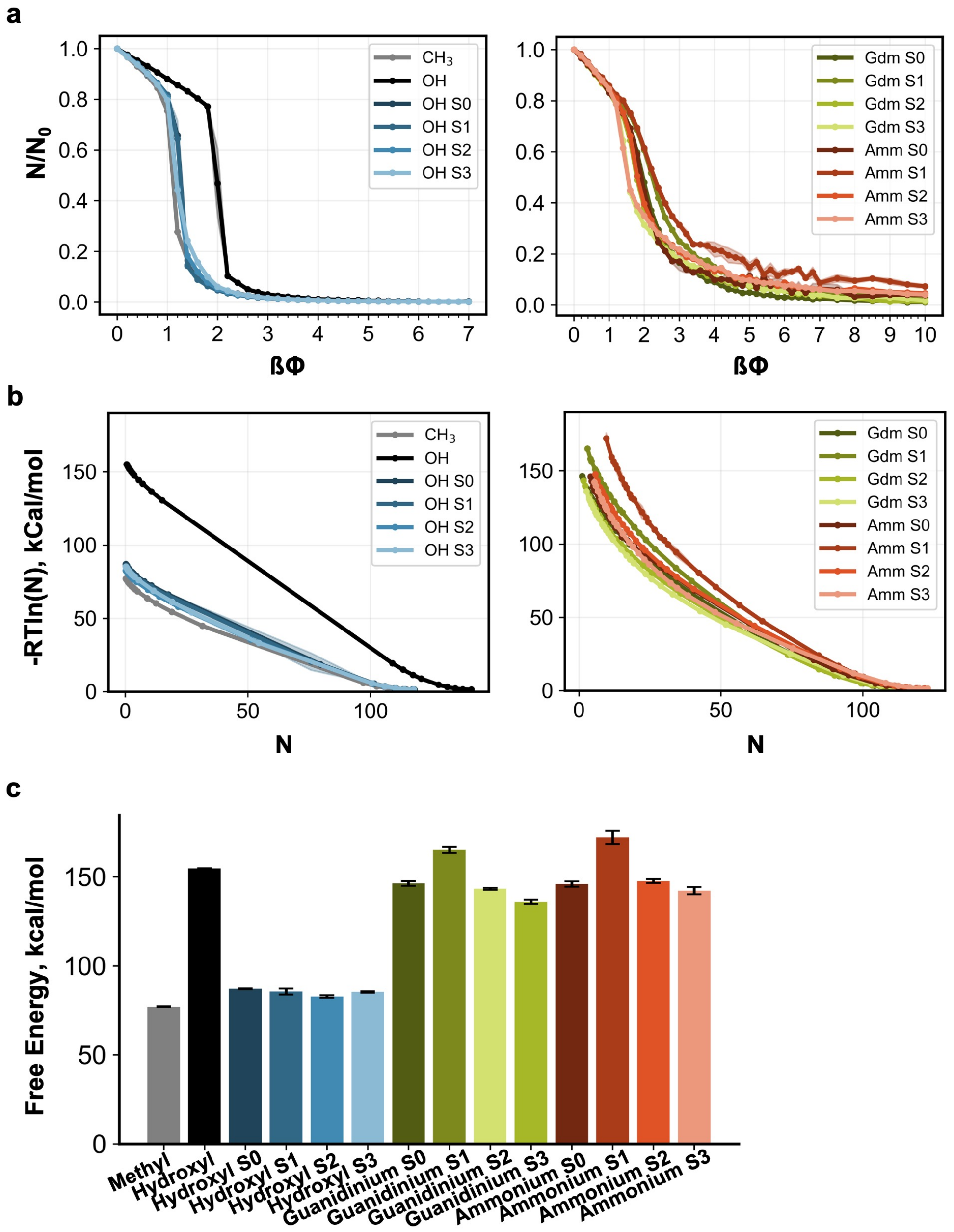

Figure S6: Effect of hydrophilic group type and arrangement on dewetting thermodynamics. (a) Normalized number of water molecules as a function of applied potential. N or N_0 denotes the average number of water molecules in the probe volume, calculated by discarding the initial 1.5 ns of a 4 ns simulation for uncharged surfaces (left panel), and the initial 3 ns of a 6 ns simulation for charged surfaces (right panel). N/N_0 represents the average from three independent simulations, with shaded error bars indicating the standard error of the mean. (b) Free energy profile as a function of number of water molecules for uncharged (left panel) and charged surfaces (right panel). (c) Free energy of cavity formation near different surfaces. The values represent the average free energy from three independent simulations, with error bars showing the standard error of the mean.

3 Potential of Mean Force (PMF) of gold nanoparticle (GNP) binding to SAM surfaces

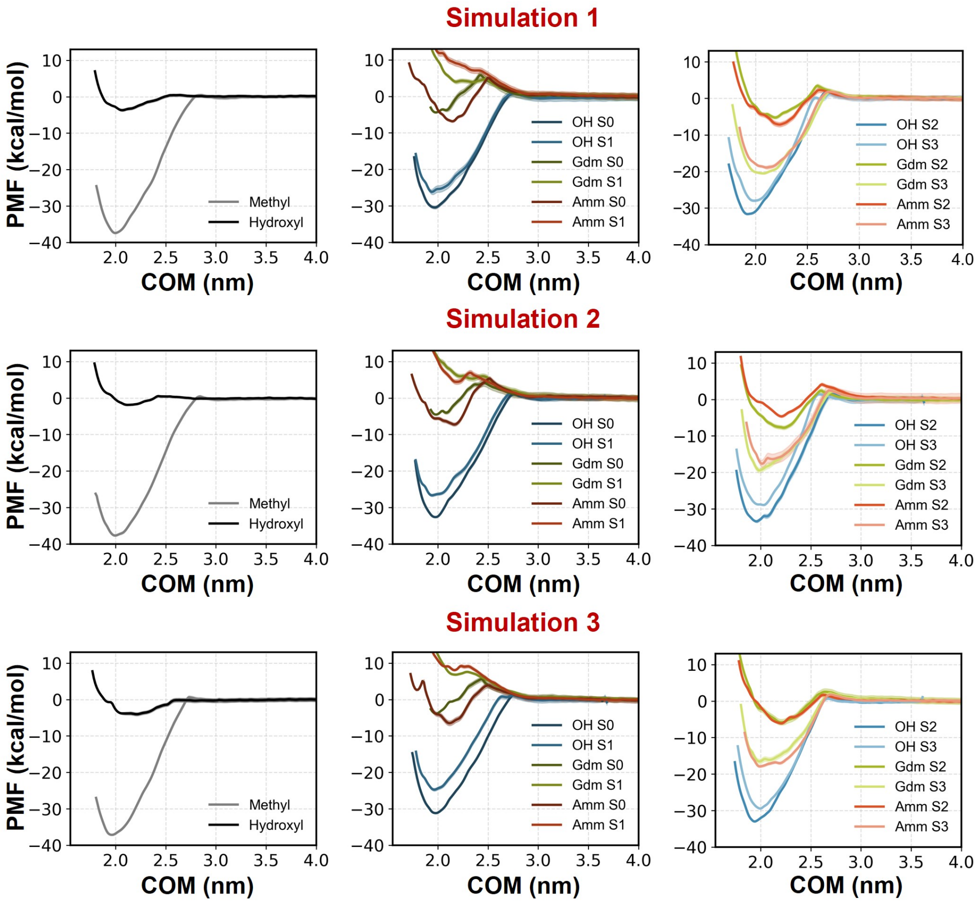

Figure S7: Potential of mean force (PMF) as a function of center of mass between the gold nanoparticle (GNP) and Self-assembled monolayers (SAM). Left panel: PMF of GNP adsorption on homogeneous methyl and homogeneous hydroxyl SAM surfaces. Middle panel: PMF of GNP adsorption on S0 and S1 configuration of hydroxyl-methyl, guanidinium-methyl, and ammonium methyl SAM surfaces. Right panel: PMF of GNP adsorption on S2 and S3 configuration of hydroxyl-methyl, guanidinium-methyl, and ammonium-methyl SAM surfaces. Three separate panels are shown for clarity. The error bars are obtained from bootstrapping and represented as shaded outline. A total of three replicate simulations were performed and PMF as a function of COM is shown as simulation 1, simulation 2 and simulation 3.

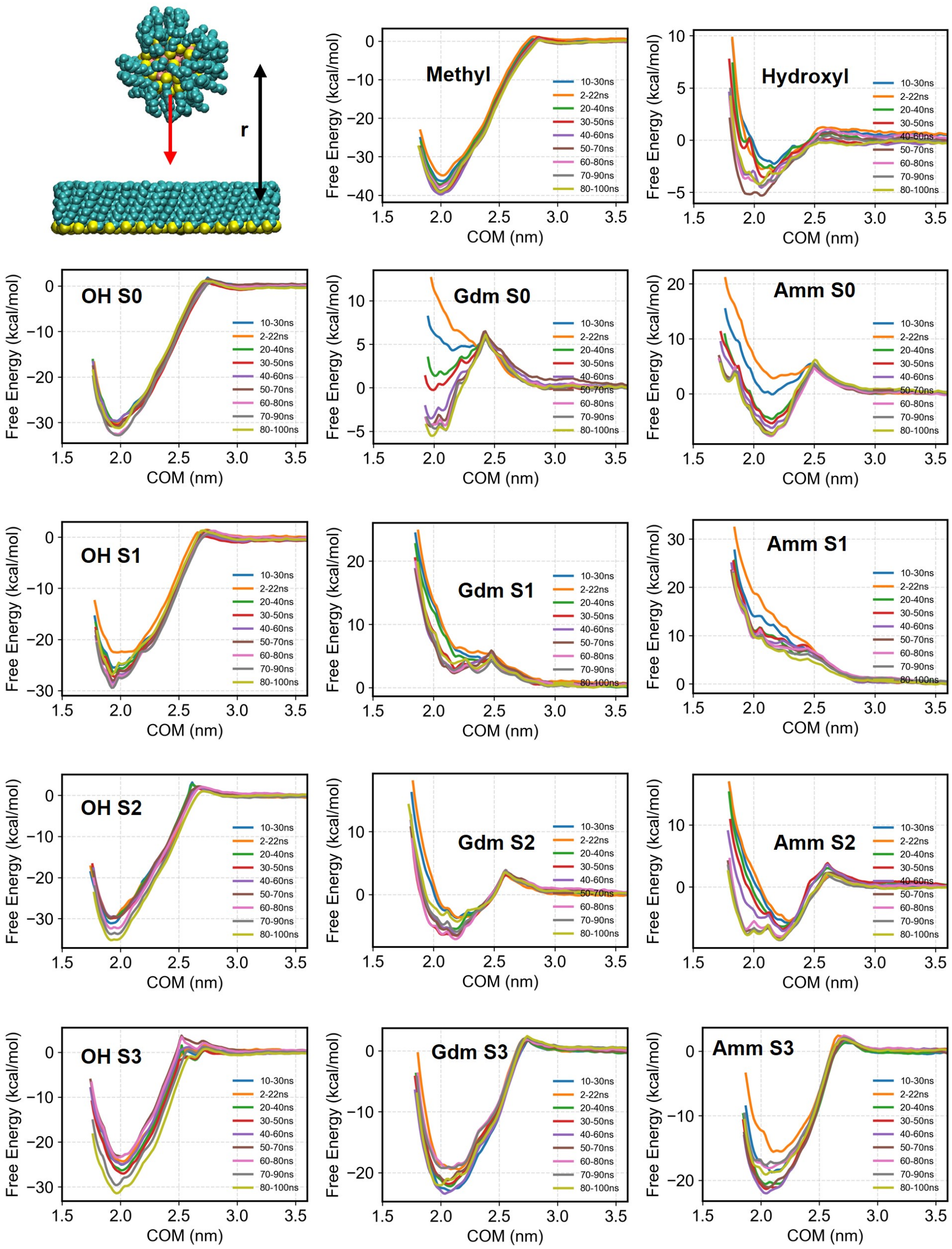

Figure S8: Potential of mean force (PMF) for GNP adsorption (simulation 1). The PMF profiles were computed using 20 ns blocks spaced 10 ns apart upto 100 ns. The SAM–GNP system was initially prepared in unbound, wet state, after which it was equilibrated. This equilibrated structure was then used to generate the starting configurations by pulling the GNP toward the surface via steered molecular dynamics (SMD) for umbrella sampling. The PMF shows substantial fluctuations up to 40 ns for Gdm S0 and 30 ns for Amm S0, suggesting that the final 60 ns and 70 ns, respectively, represent equilibrated trajectories. All other systems exhibit minimal sensitivity to the analysis window; thus, a 2 ns equilibration time was used. In the schematic, only the surface and solute are shown, with hydrogen atoms omitted for clarity.

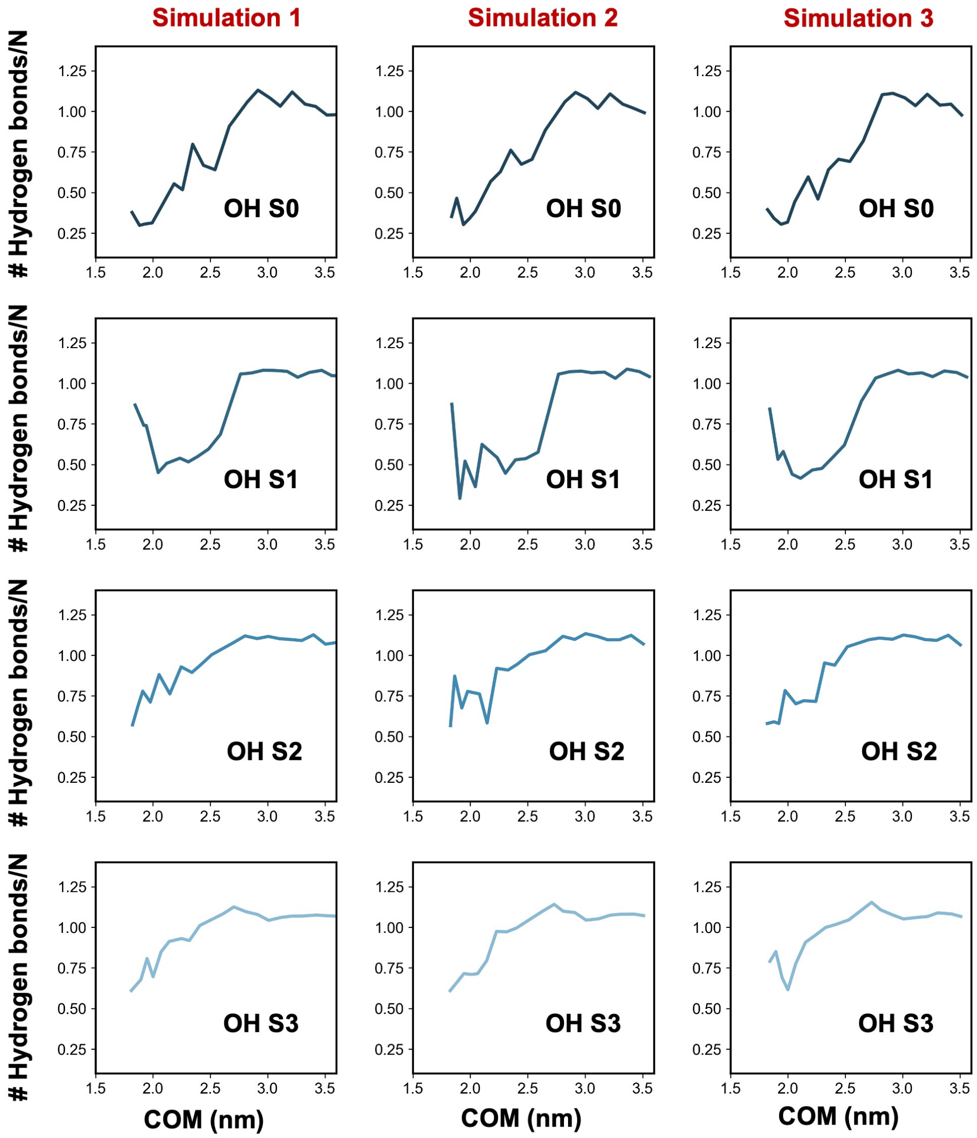

Figure S9: SAM-water hydrogen bonding as a function of center of mass between the gold nanoparticle (GNP) and Self-assembled monolayers (SAM). The average number of hydrogen bonds per hydrophilic group (N denotes the number of hydrophilic groups) includes both donor and acceptor interactions.

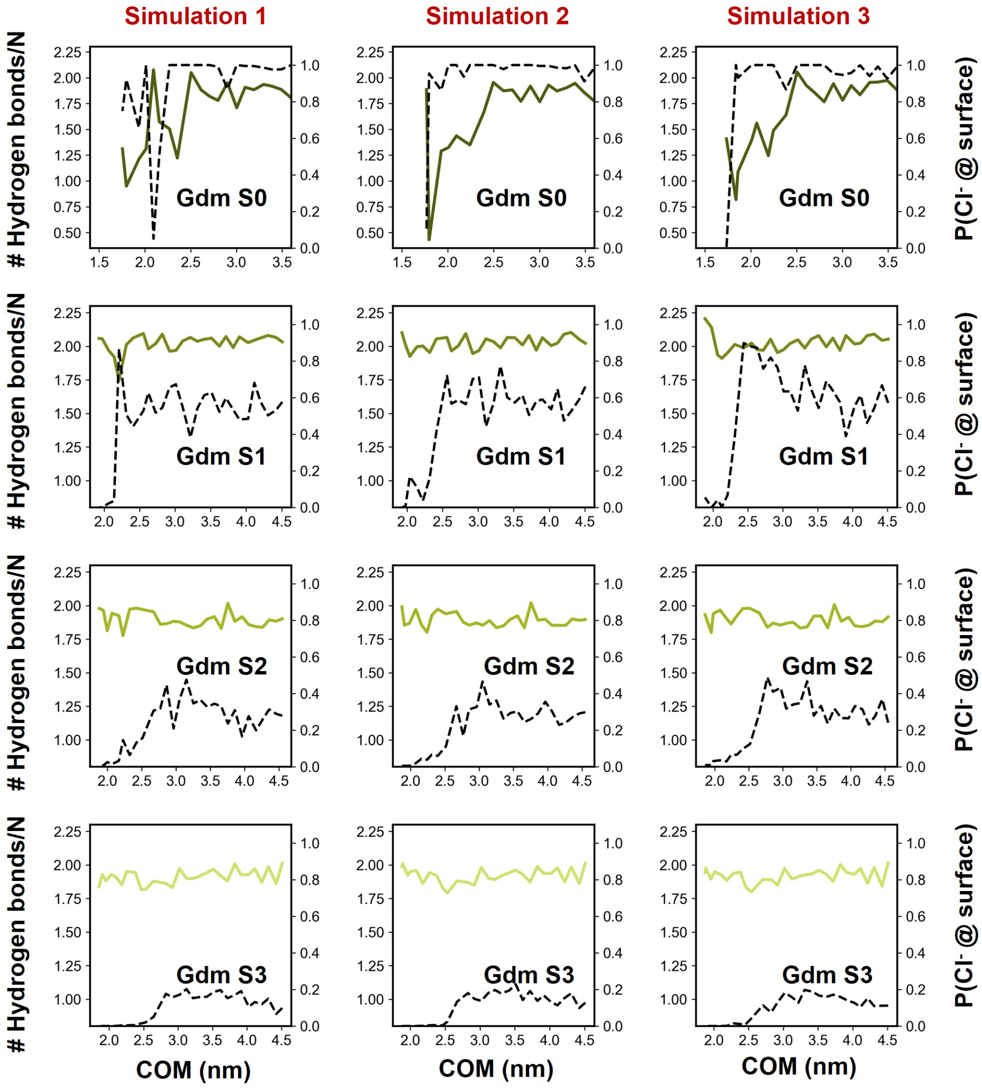

Figure S10: SAM-water hydrogen bonding and probability of observing a counter ion at GNP binding region as a function of center of mass between the gold nanoparticle (GNP) and Self assembled monolayers (SAM). The primary y-axis (left) represents the average number of SAM water hydrogen bonds per hydrophilic group (where N denotes the number of hydrophilic groups), shown as a solid line. The secondary y-axis (right) indicates the probability of observing a counterion within the 1 nm radius of the center of mass of the three charged functional groups, shown as a dashed line.

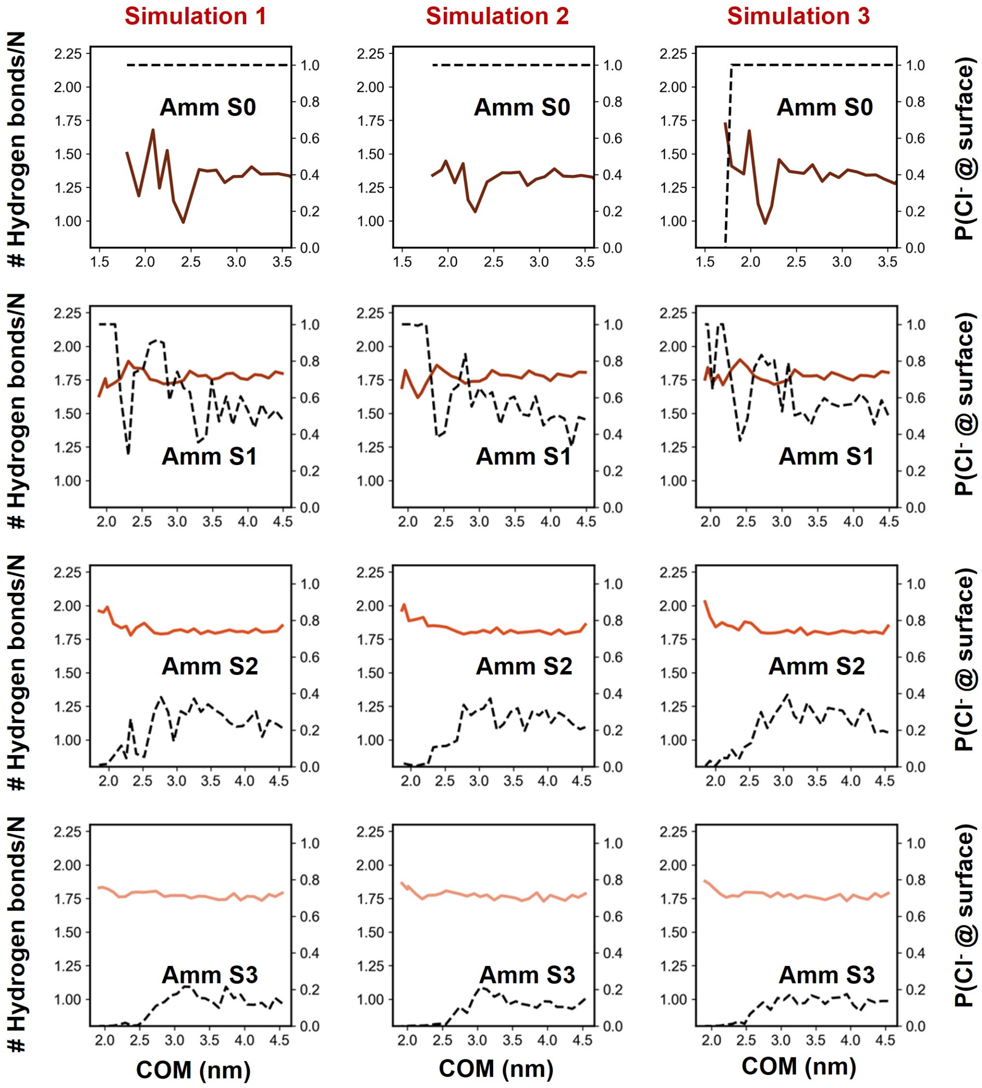

Figure S11: SAM-water hydrogen bonding and probability of observing a counter ion at GNP binding region as a function of center of mass between the gold nanoparticle (GNP) and Self assembled monolayers (SAM). The primary y-axis (left) represents the average number of SAM water hydrogen bonds per hydrophilic group (where N denotes the number of hydrophilic groups), shown as a solid line. The secondary y-axis (right) indicates the probability of observing a counterion within a 1 nm radius of the center of mass of the three charged functional groups, shown as a dashed line.

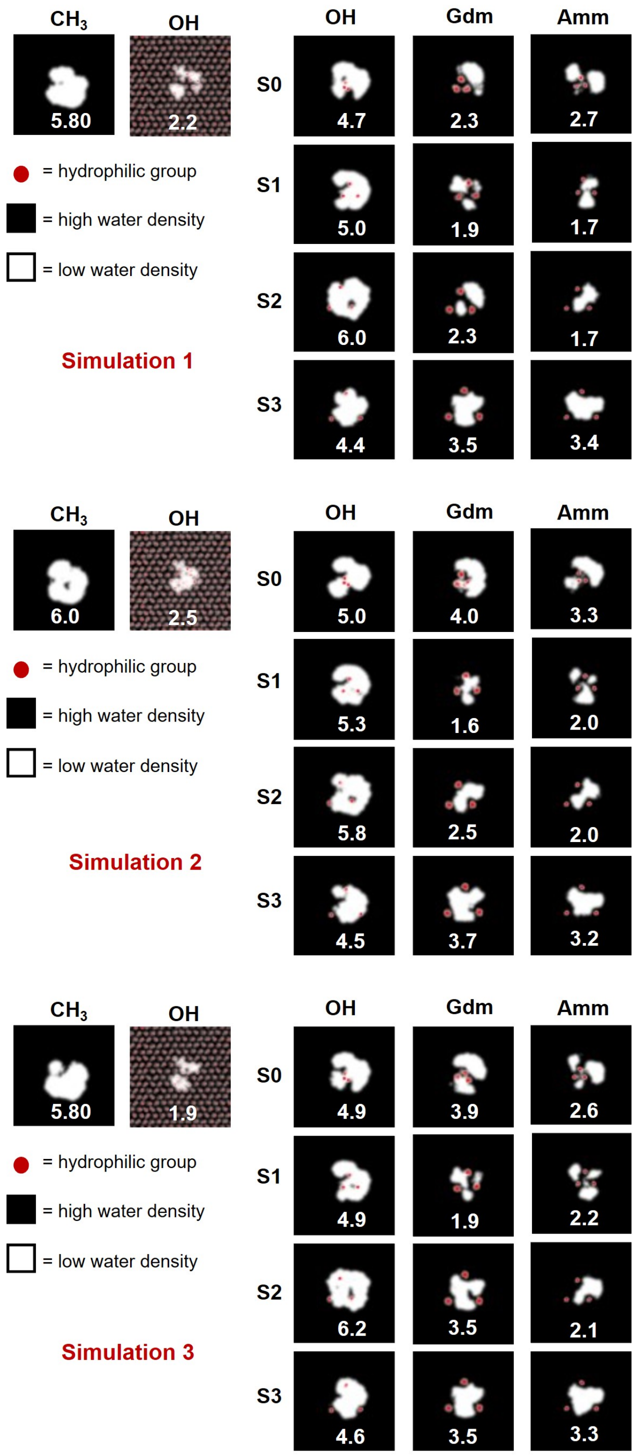

Figure S12: Dewetted area on SAM at favorable bound state of GNP corresponding to PMF minimum (In cases where favorable binding does not exist, we analyzed the dewetted area at distance corresponding to favorable binding of other system). The threshold for low water density is 5 water molecules/nm^3. The numbers represent the dewetted area in /nm^2.

4 Potential of mean force (PMF) of hydrophobin binding to SAM surfaces

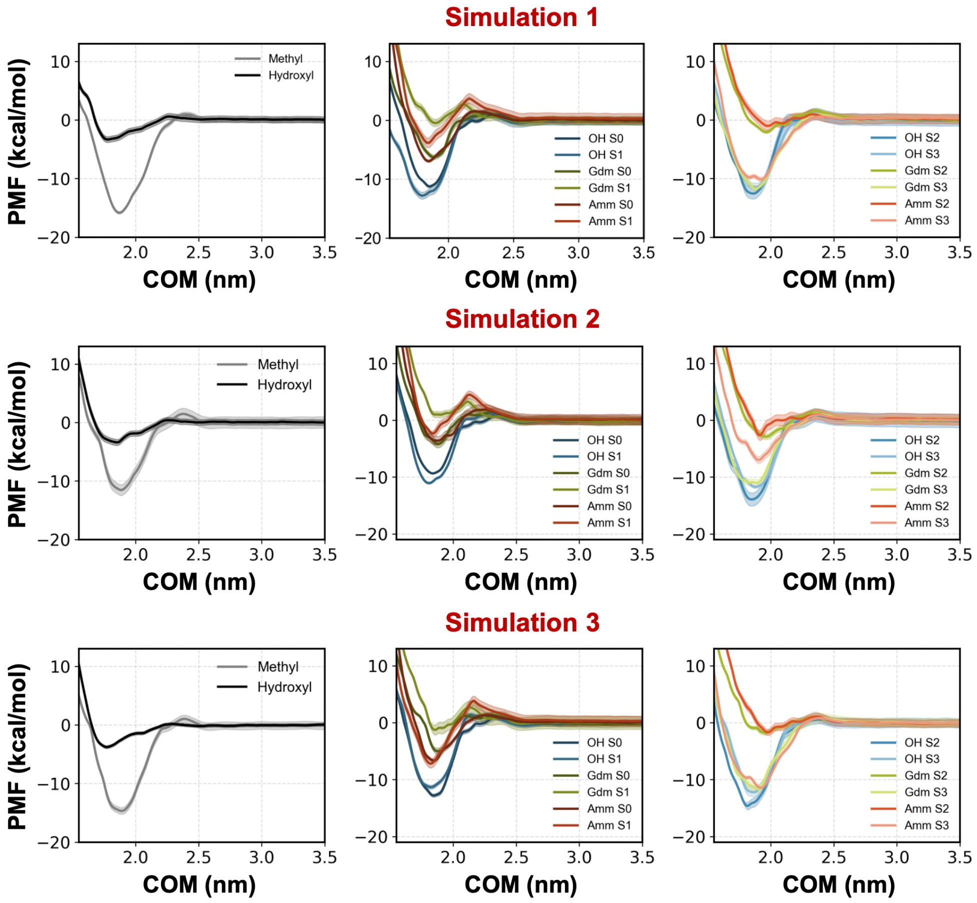

Figure S13: Potential of mean force (PMF) as a function of center of mass between the hydrophobin and Self-assembled monolayers (SAM). Left panel: PMF of hydrophobin adsorption on homogeneous methyl and homogeneous hydroxyl SAM surfaces. Middle panel: PMF of hydrophobin adsorption S0 and S1 configuration of hydroxyl-methyl, guanidinium-methyl, and ammonium-methyl SAM system. Right panel: PMF of hydrophobin adsorption in S2 and S3 configuration of hydroxyl-methyl, guanidinium-methyl, and ammonium-methyl SAM system. Three separate panels are shown for clarity. The error bars are obtained from bootstrapping and represented as shaded outline. A total of three replicate simulations were performed and PMF as a function of COM is shown as simulation 1, simulation 2 and simulation 3.

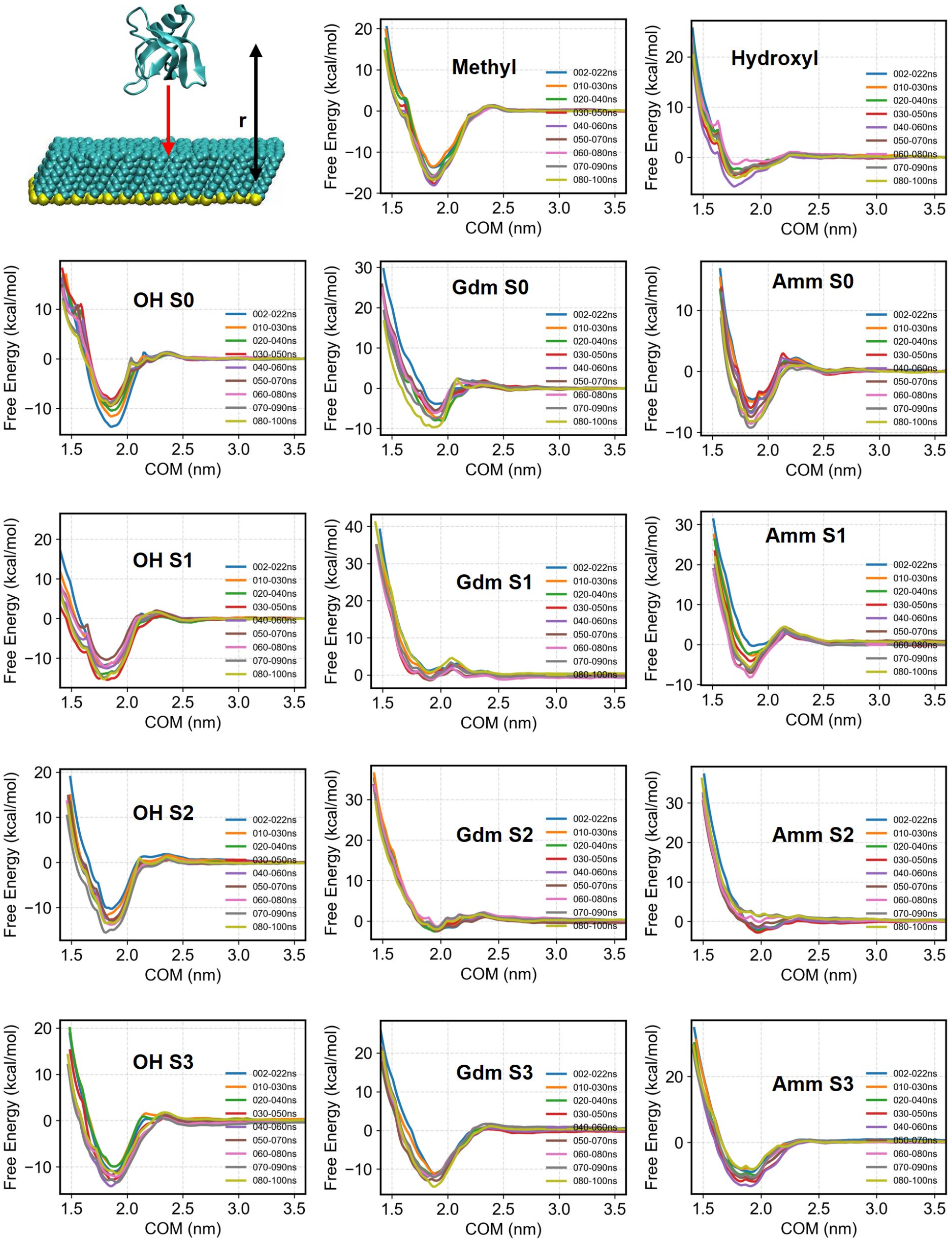

Figure S14: Potential of mean force (PMF) for hydrophobin adsorption (simulation 1). The PMF profiles were computed using 20 ns blocks spaced 10 ns apart upto 100 ns. The SAM–hydrophobin system was initially prepared in unbound, wet state, after which it was equilibrated. This equilibrated structure was then used to generate the starting configurations by pulling the GNP toward the surface via steered molecular dynamics (SMD) for umbrella sampling. All systems exhibit minimal sensitivity to the analysis window; thus, a 2 ns equilibration time was used. In the schematic, only the surface and solute are shown, with hydrogen atoms omitted for clarity.

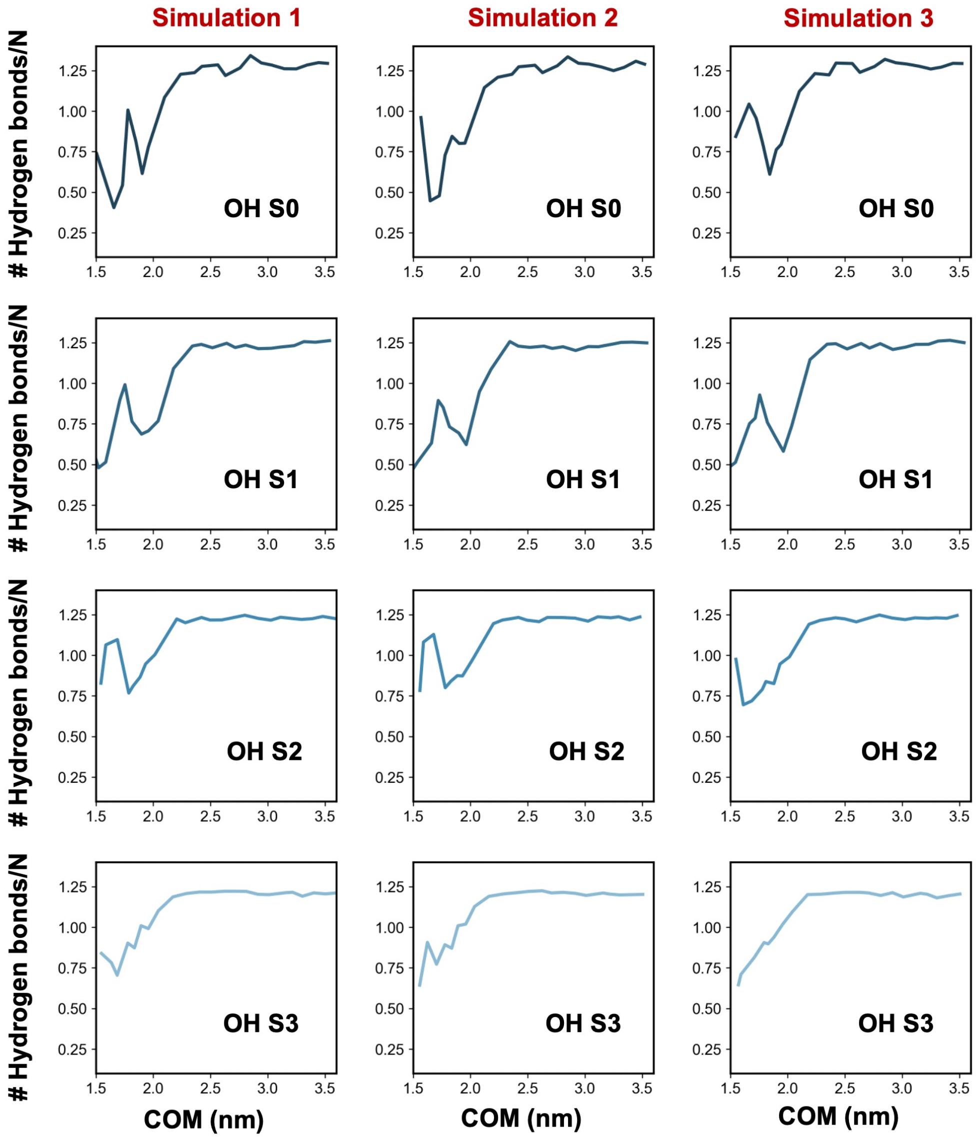

Figure S15: SAM-water hydrogen bonding as a function of center of mass between the hydrophobin and Self-assembled monolayers (SAM). The average number of hydrogen bonds per hydrophilic group (N denotes the number of hydrophilic groups) includes both donor and acceptor interactions.

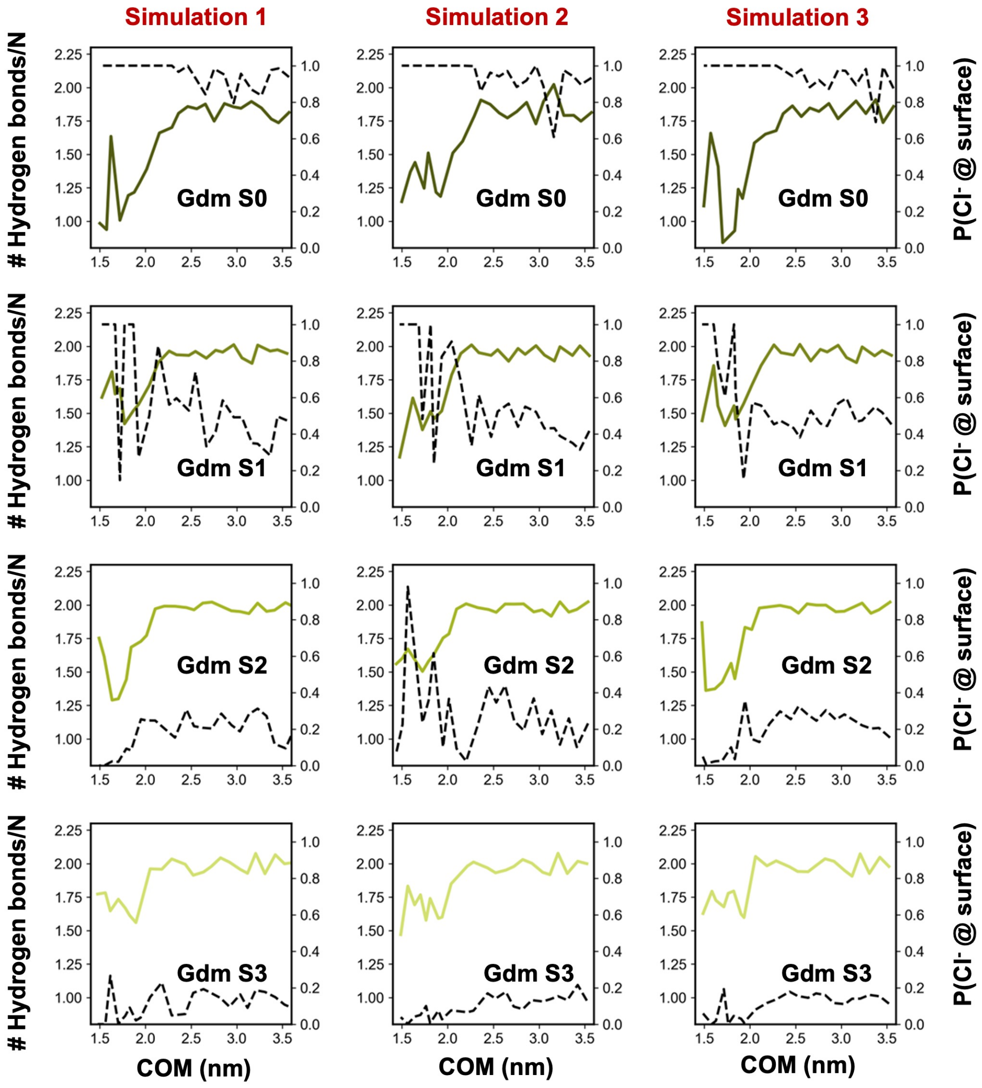

Figure S16: SAM-water hydrogen bonding and probability of counter ion being at hydrophobin binding region as a function of center of mass between the hydrophobin and Self-assembled monolayers (SAM). The primary y-axis (left) represents the average number of SAM–water hydrogen bonds per hydrophilic group (where N denotes the number of hydrophilic groups), shown as a solid line. The secondary y-axis (right) indicates the probability of observing a counterion within a 1 nm radius of the center of mass of the three charged functional groups as shown as a dashed line.

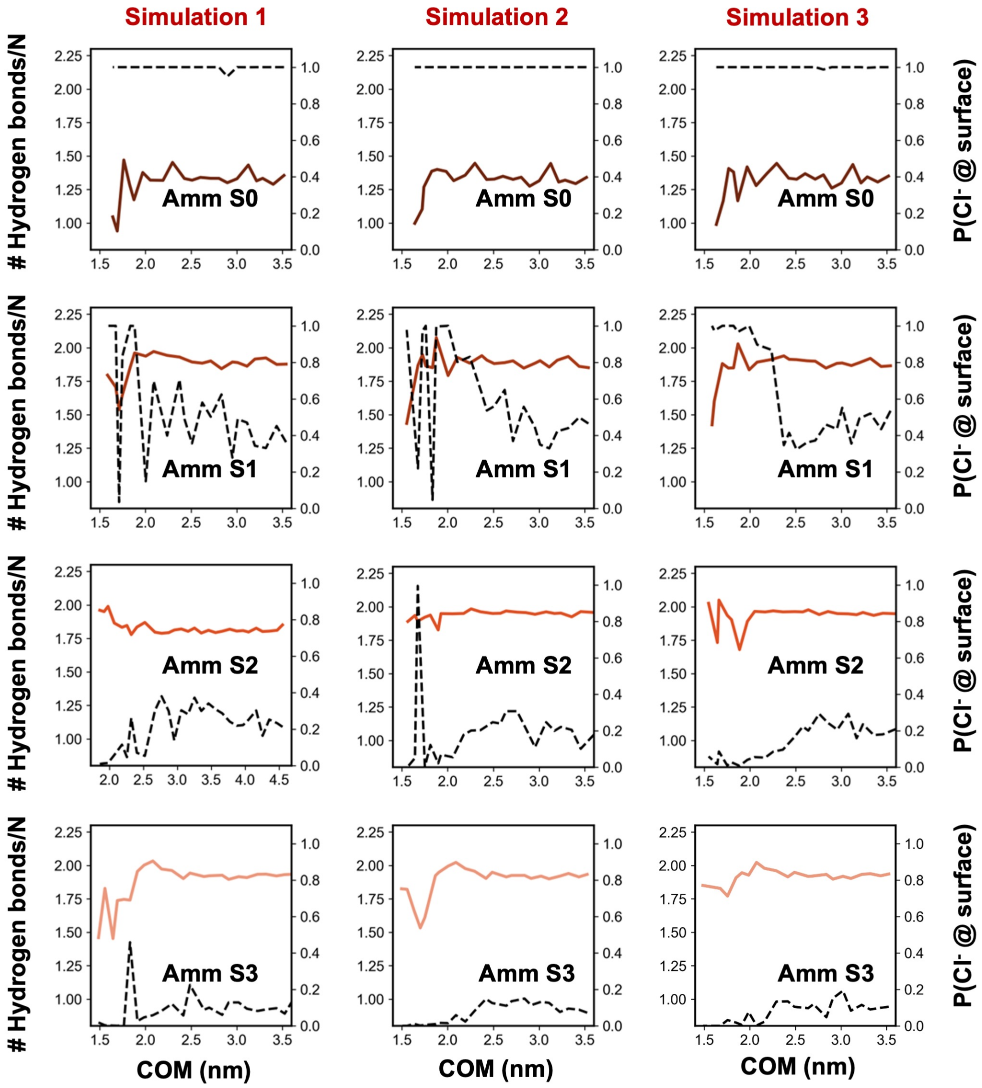

Figure S17: SAM-water hydrogen bonding and probability of counter ion being at hydrophobin binding region as a function of center of mass between the hydrophobin and Self-assembled monolayers (SAM). The primary y-axis (left) represents the average number of SAM–water hydrogen bonds per hydrophilic group (where N denotes the number of hydrophilic groups), shown as a solid line. The secondary y-axis (right) indicates the probability of observing a counterion within a 1 nm radius of the center of mass of the three charged functional groups as shown as a dashed line.

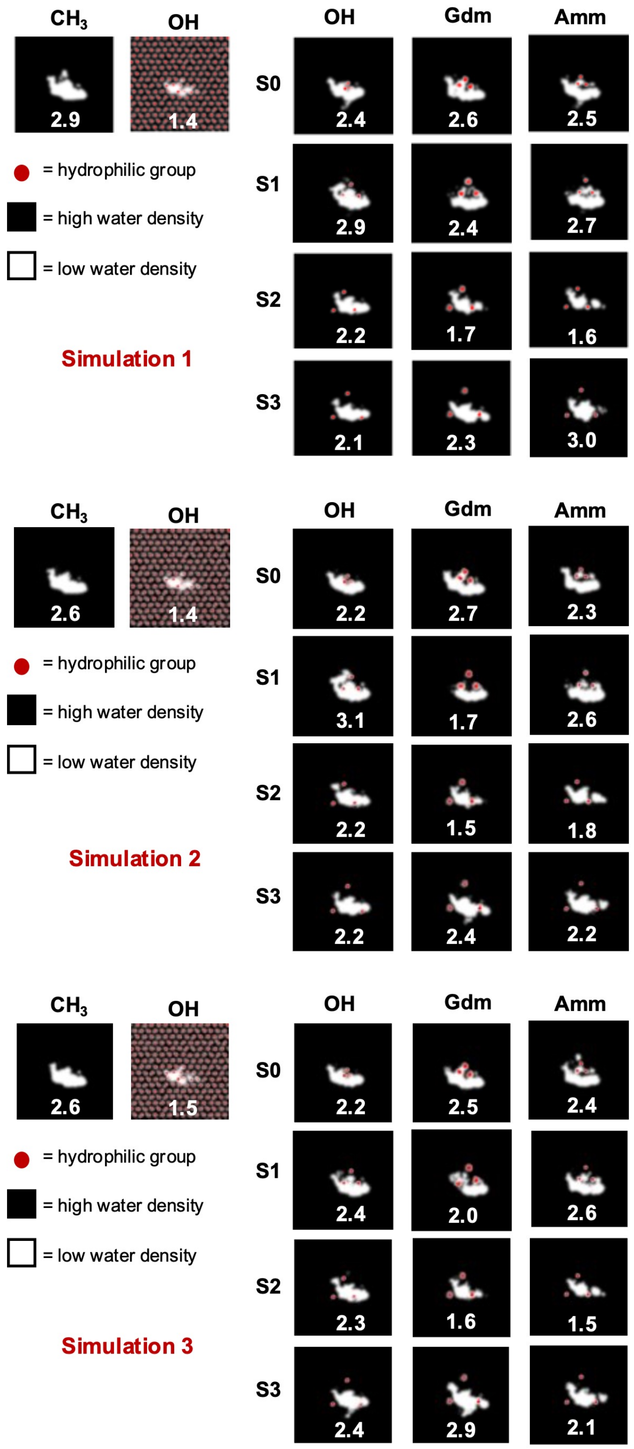

Figure S18: Dewetted area on SAM at favorable bound state of hydrophobin corresponding to PMF minimum (In cases where favorable binding does not exist, we analyzed the dewetted area at distance corresponding to favorable binding of other systems). The threshold for low water density is 5 water molecules/nm^3. The numbers represent the dewetted area in nm^2.

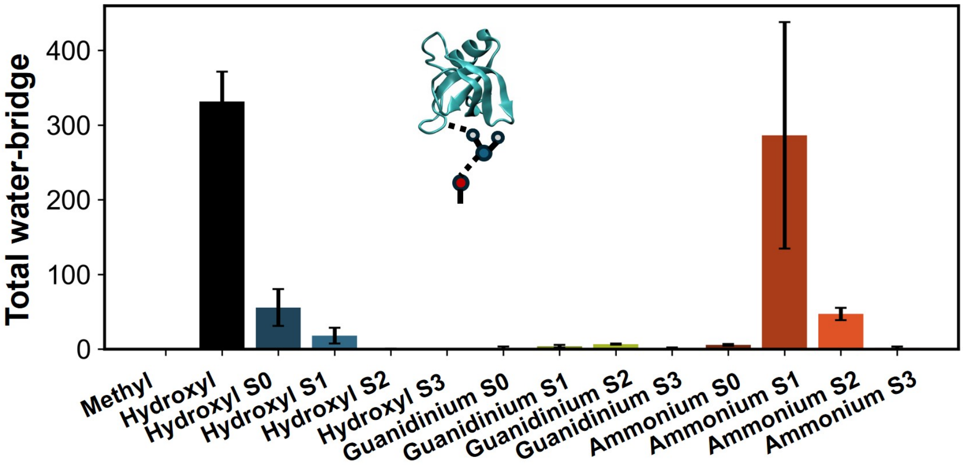

Figure S19: Total water-bridge count in the bound state of hydrophobin. The total represents the sum of all water bridges observed in 980 equally spaced frames from 2 ns to 100 ns. A water-bridge is defined as a single water molecule simultaneously forming hydrogen bonds with both hydrophobin and the SAM surface. Error bars indicate the standard error of the mean. The large standard error of the mean (total number of water bridge in simulation 1, 2 and 3 were 107,588, and 164 respectively) for ammonium S1 primarily arises from persistent water bridge formation (22 percent of analyzed frames) between the carboxylate oxygen of aspartic acid in hydrophobin and the SAM hydrophilic group in the second replicate simulation Hydrogen bonds are identified using a geometric criterion: donor–acceptor (D–A) distance ≤ 0.35 nm and D–H–A angle $\geq 150°$.

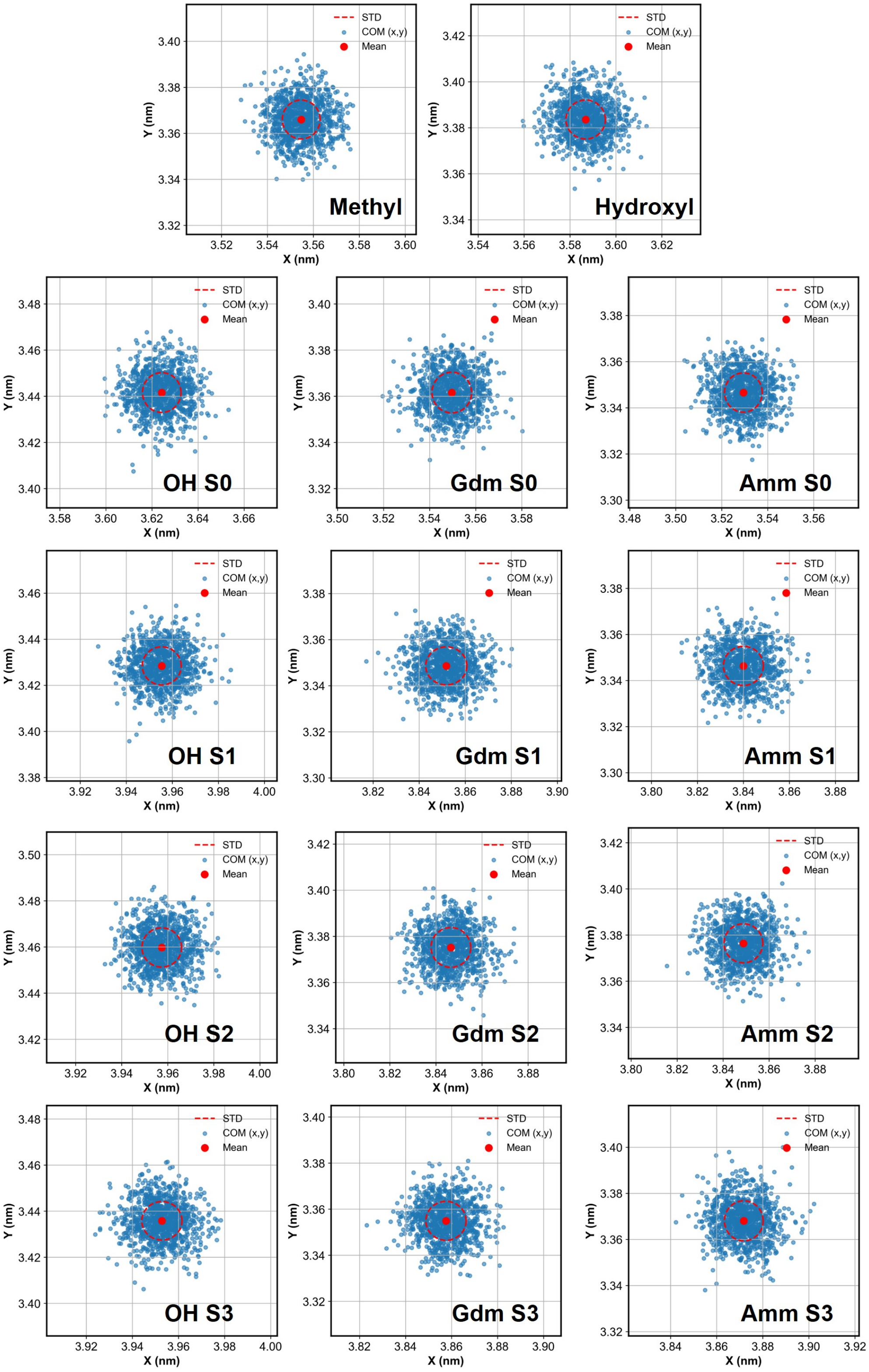

Figure S20: Center-of-mass (COM) positions of the sulfur atoms (restrained within the hydrophobin) in the bound state from Simulation 1, sampled at equally spaced frames between 2 ns and 100 ns. All plots use a fixed 1 nm span on both x- and y-axes to enable direct comparison of fluctuation in x and y direction.

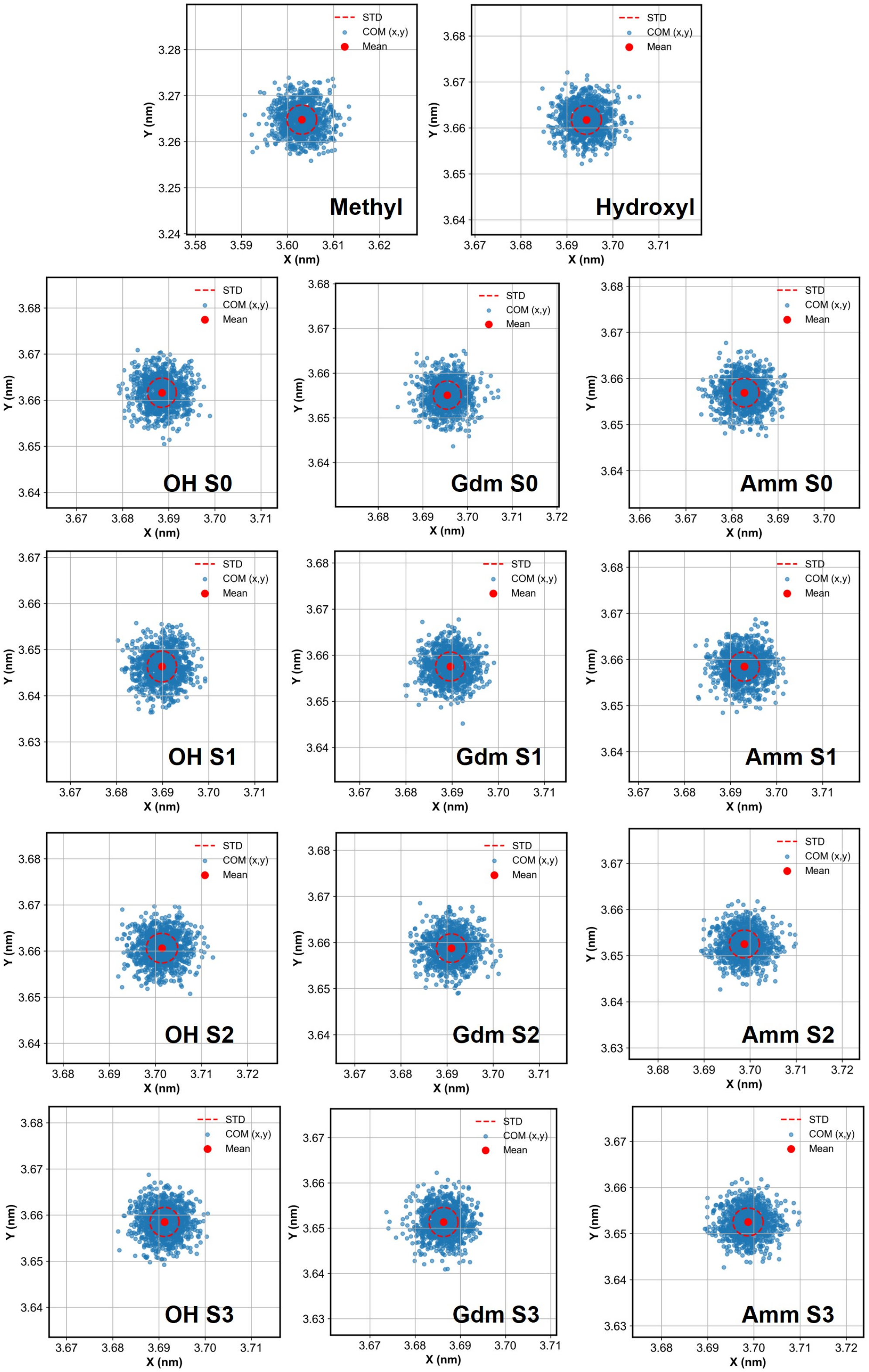

Figure S21: Center-of-mass (COM) positions of the AU atoms (restrained within the GNP) in the bound state from Simulation 1, sampled at equally spaced frames between 2 ns and 100 ns. All plots use a fixed 0.5 nm span on both x- and y-axes to enable direct comparison of fluctuation in x and y direction.

5 Exploring hysteresis and convergence in binding affinity calculation

Umbrella sampling for PMF calculations along a collective variable can exhibit hysteresis, meaning results may not be reversible along the path.[6,7] In the main manuscript, our PMFs were obtained by equilibrating the solute in bulk and then pulling it toward the surface, with sufficient sampling and error bars estimated from three independent replicates. To assess potential hysteresis, we also computed PMFs starting from a dry-bound equilibrium state, where the solute was initially placed at the surface in a dewetted configuration, then solvated and equilibrated. The PMF was subsequently generated by moving the solute away from the surface. The resulting profiles are shown in the following plots.

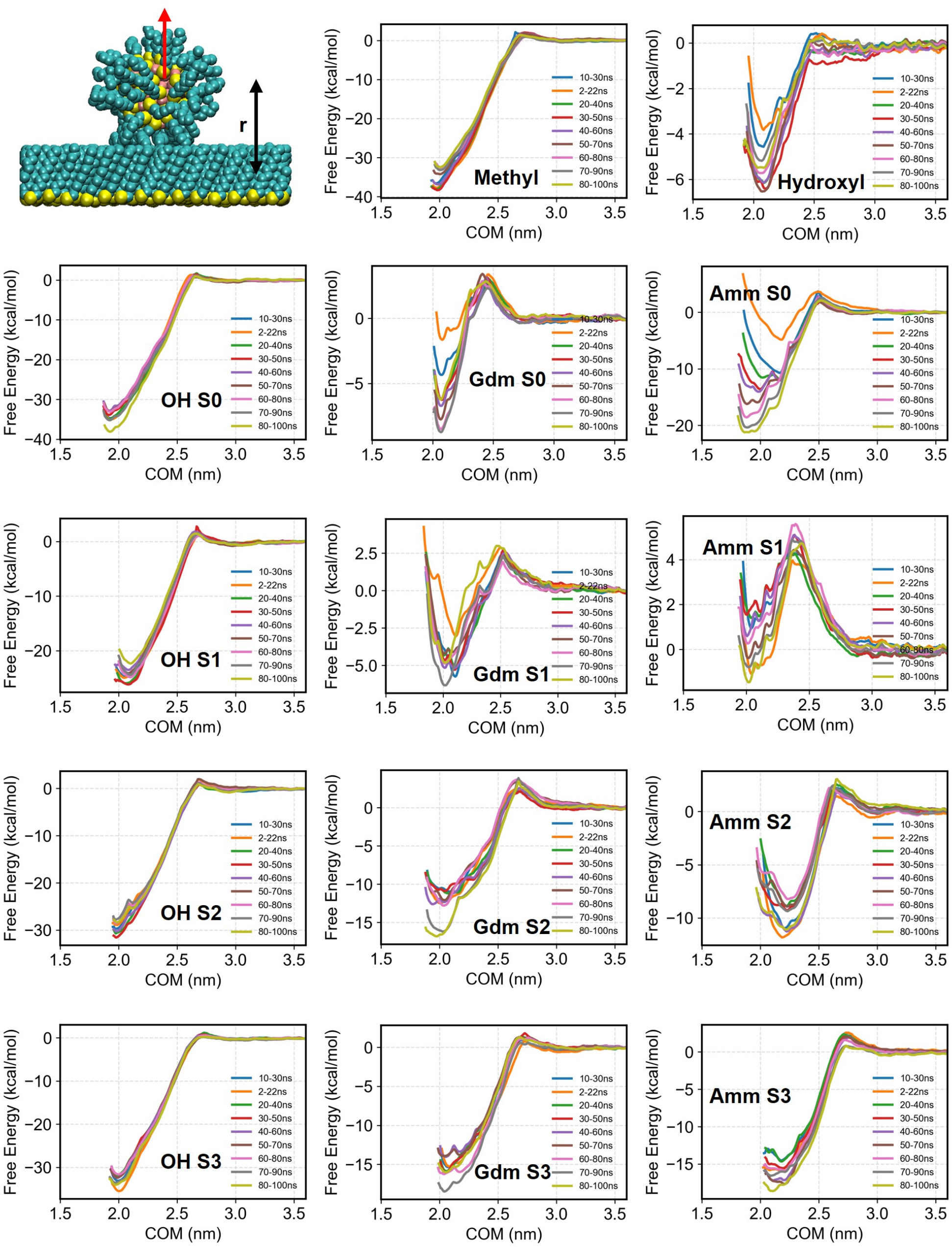

Figure S22: Potential of mean force (PMF) for GNP desorption. The PMF profiles were computed using 20 ns blocks spaced 10 ns apart upto 100 ns. The SAM–GNP system was initially prepared in a bound, dry state, after which it was solvated and equilibrated. This equilibrated structure was then used to generate the starting configurations for umbrella sampling via steered molecular dynamics (SMD).The PMF exhibits significant fluctuations up to approximately 60 ns, indicating that the final 40 ns likely represent an equilibrated trajectory.In the schematic, only the surface and solute are shown, with hydrogen atoms omitted for clarity.

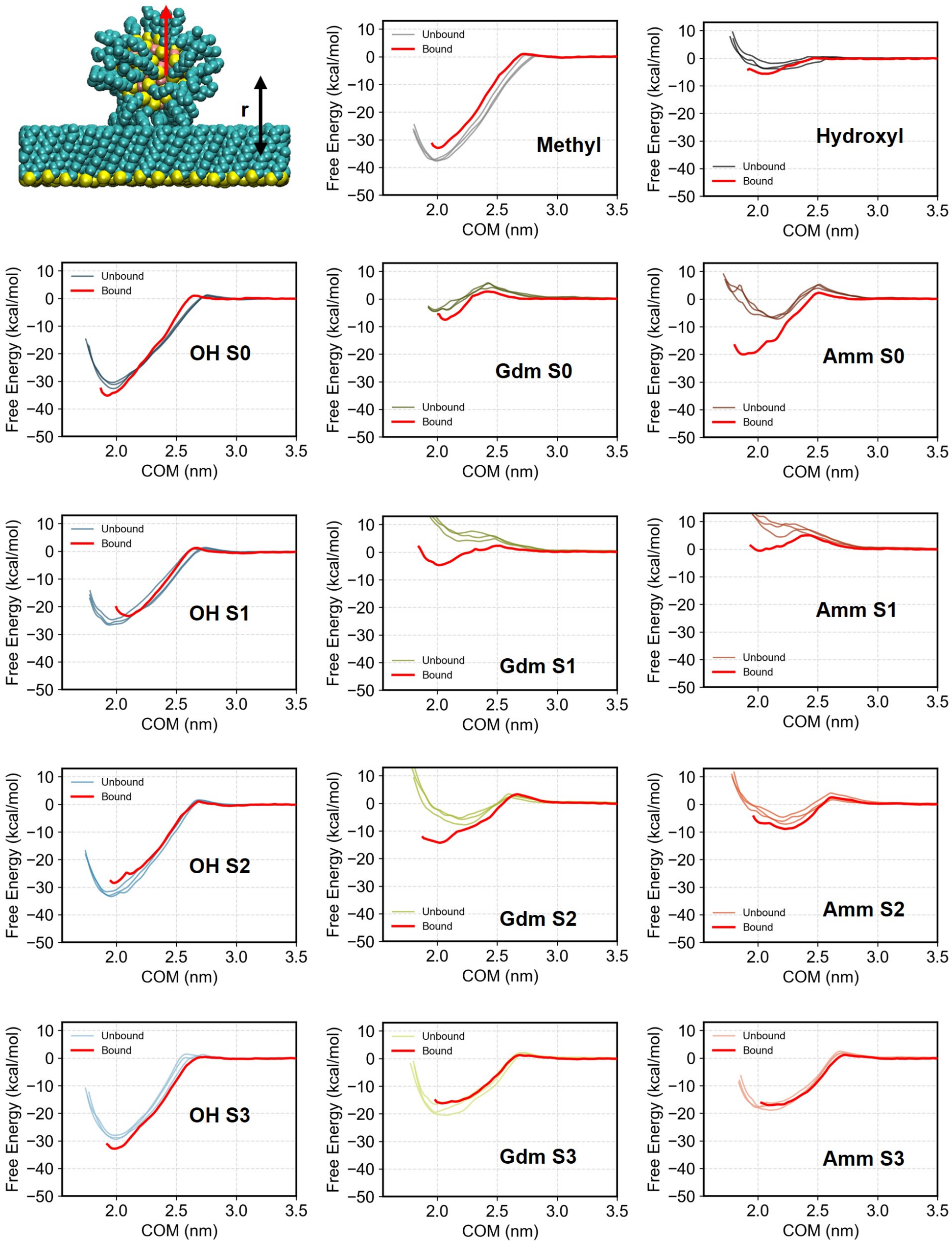

Figure S23: Potential of mean force (PMF) comparison for GNP dry-bound vs wet-unbound initial state simulation. The PMF profile was computed using last 40 ns for dry-bound simulation. For wet-unbound initial state simulations, the PMF profiles are same as reported in the main manuscript for 3 replicate simulations. In the schematic, only the surface and solute are shown, with hydrogen atoms omitted for clarity.

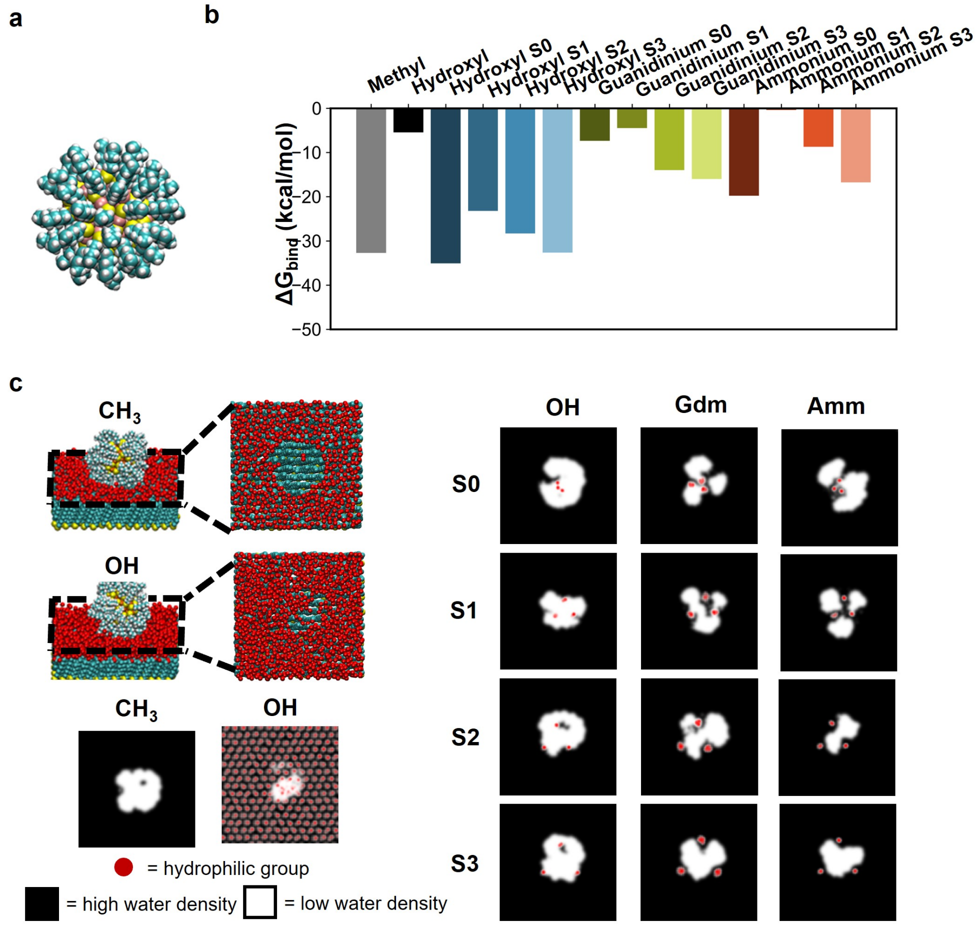

Figure S24: Free energy and dewetting profile in dry-bound initial state simulation. (a) Heptane thiol functionalized GNP (cyan: carbon, white: hydrogen, yellow: sulfur, orange: gold) (b) Free energy of GNP binding on different surfaces. The error bars represent the standard error of the mean obtained from three independent simulations. (c) Dewetted area of GNP at favorable bound state corresponding to PMF minimum (In cases where favorable binding does not exist, we analyzed the dewetted area at distance corresponding to favorable binding of other system). The threshold for low water density is 5 molecules/nm^3. A schematic is also shown for GNP adsorbed on homogeneous methyl and hydroxyl SAMs. Only first two hydration shell water oxygen is shown for clarity.

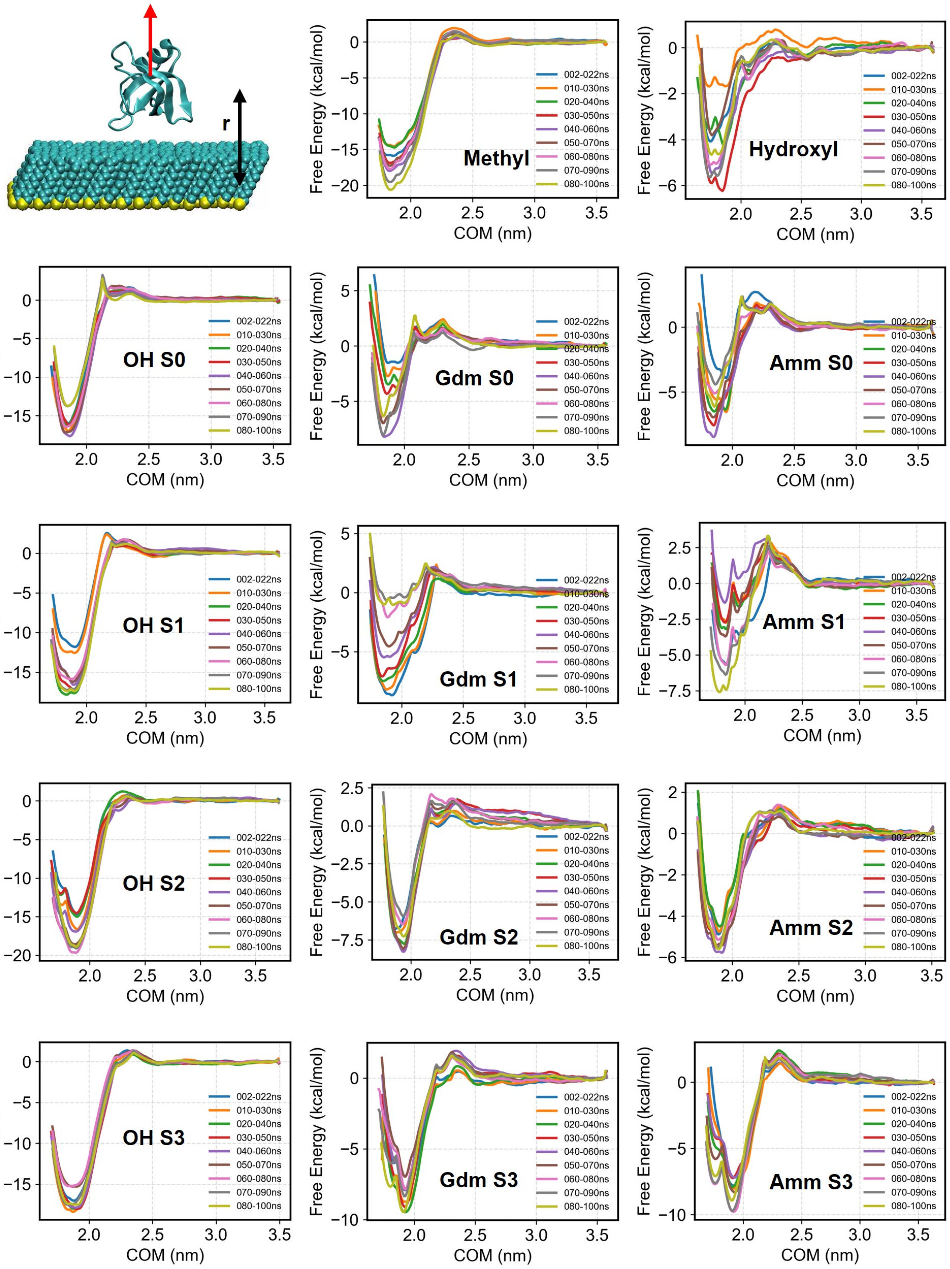

Figure S25: Potential of mean force (PMF) for hydrophobin desorption. The PMF profiles were computed using 20 ns blocks spaced 10 ns apart upto 100 ns. The SAM–hydrophobin system was initially prepared in a bound, dry state, after which it was solvated and equilibrated. This equilibrated structure was then used to generate the starting configurations for umbrella sampling via steered molecular dynamics (SMD).The PMF exhibits significant fluctuations up to approximately 60 ns, indicating that the final 40 ns likely represent an equilibrated trajectory. In the schematic, only the surface and solute are shown, with hydrogen atoms omitted for clarity.

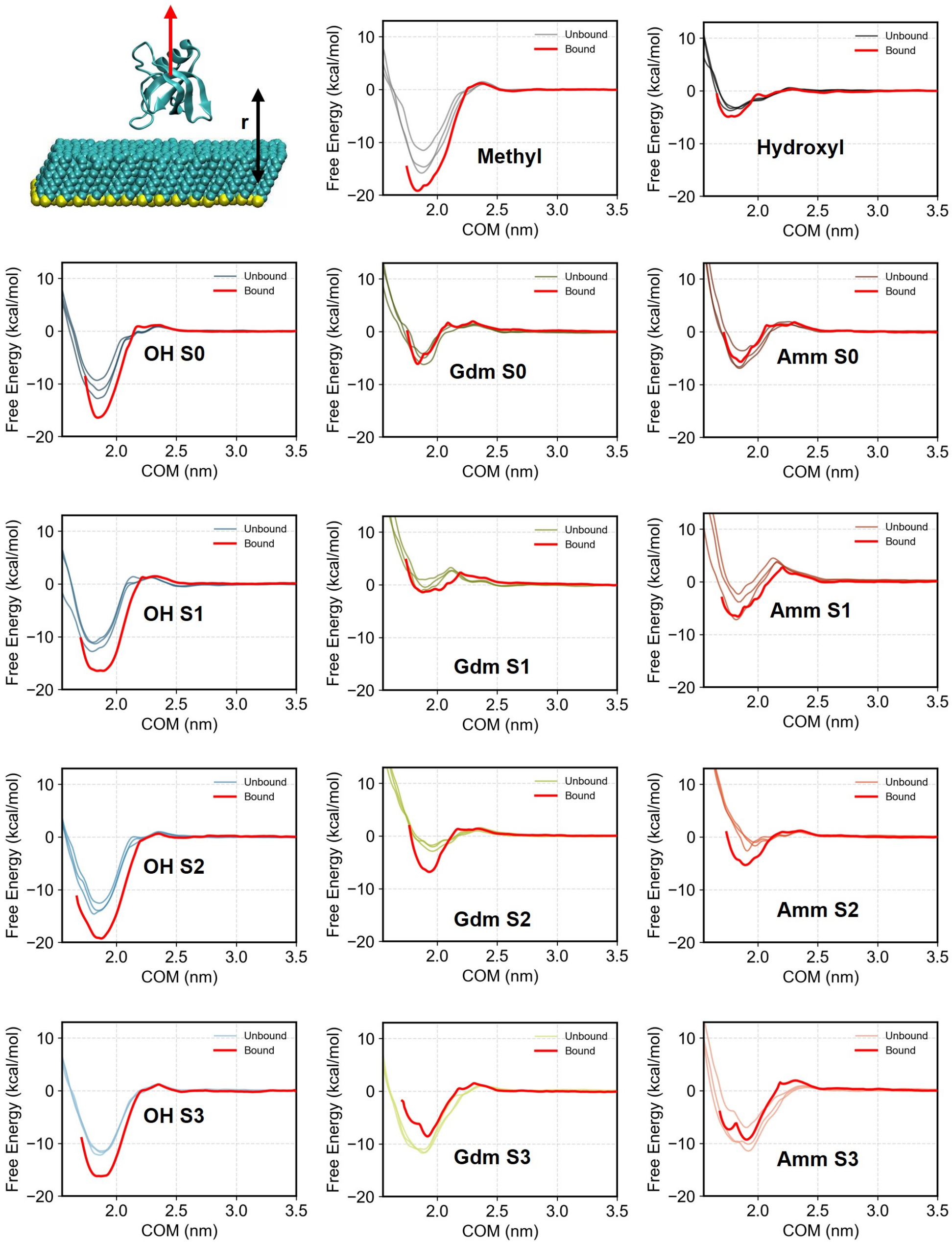

Figure S26: Potential of mean force (PMF) comparison for hydrophobin dry-bound vs wet-unbound initial state simulation. The PMF profile was computed using last 40 ns for dry-bound simulation. For wet-unbound initial state simulations, the PMF profiles are same as reported in the main manuscript for 3 replicate simulations. In the schematic, only the surface and solute are shown, with hydrogen atoms omitted for clarity.

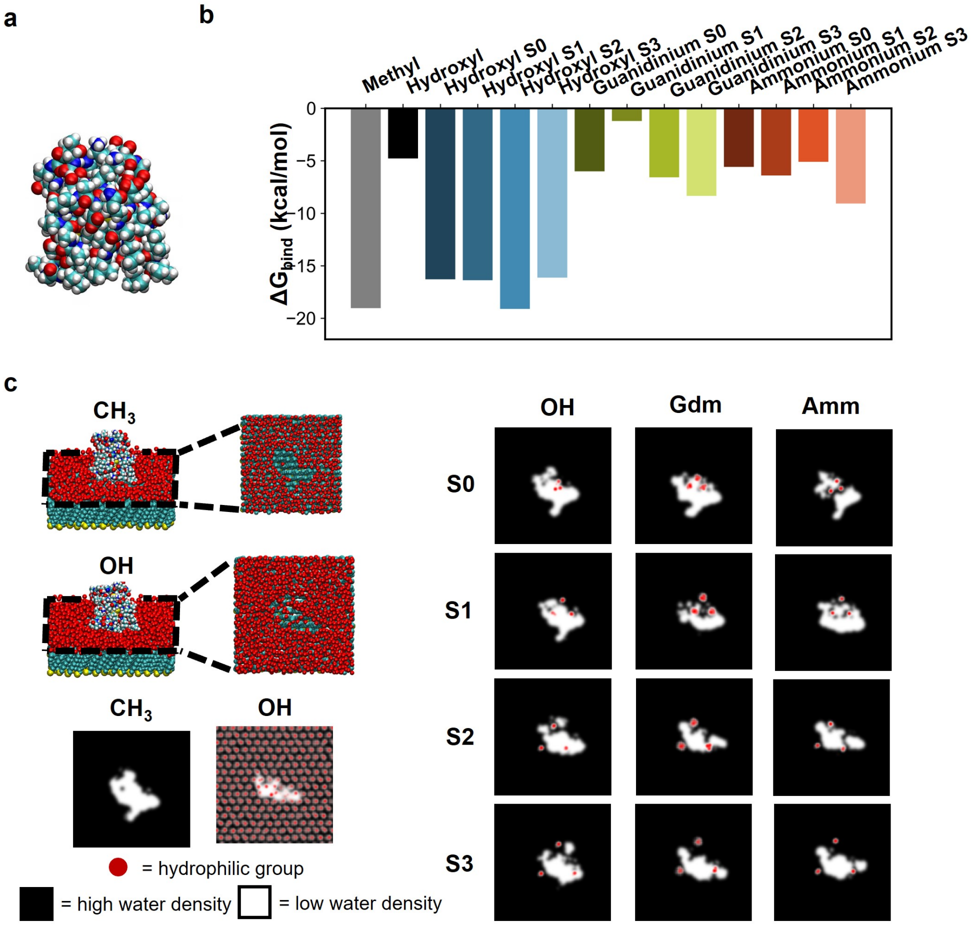

Figure S27: Free energy and dewetting profile in dry-bound initial state simulation. (a) Hydrophobin (red: oxygen, cyan: carbon, yellow: sulfur, white: hydrogen, and blue: nitrogen) (b) Free energy of hydrophobin binding on different surfaces. The error bars represent the standard error of the mean obtained from three independent simulations. (c) Dewetted area of the hydrophobin at favorable bound state corresponding to PMF minimum (In cases where favorable binding does not exist, we analyzed the footprint at distance corresponding to favorable binding of other system). The threshold for low water density is 5 molecules/nm^3. A schematic is also shown for hydrophobin GNP adsorbed on homogeneous methyl and hydroxyl SAMs. Only first two hydration shell water oxygen is shown for clarity.

References

[1] Sousa da Silva, A. W.; Vranken, W. F. ACPYPE-Antechamber python parser interface. *BMC research notes* **2012**, *5*, 367.

[2] Izadi, S.; Anandakrishnan, R.; Onufriev, A. V. Building water models: a different approach. *The journal of physical chemistry letters* **2014**, *5*, 3863–3871.

[3] Bussi, G.; Donadio, D.; Parrinello, M. Canonical sampling through velocity rescaling. *The Journal of chemical physics* **2007**, *126*.

[4] Bernetti, M.; Bussi, G. Pressure control using stochastic cell rescaling. *The Journal of Chemical Physics* **2020**, *153*.

[5] Hess, B.; Bekker, H.; Berendsen, H. J.; Fraaije, J. G. LINCS: A linear constraint solver for molecular simulations. *Journal of computational chemistry* **1997**, *18*, 1463–1472.

[6] Lichtinger, S. M.; Biggin, P. C. Tackling hysteresis in conformational sampling: how to be forgetful with MEMENTO. *Journal of Chemical Theory and Computation* **2023**, *19*, 3705–3720.

[7] Perthold, J. W.; Oostenbrink, C. Simulation of reversible protein–protein binding and calculation of binding free energies using perturbed distance restraints. *Journal of chemical theory and computation* **2017**, *13*, 5697–5708.
