# Supplementary figures and images for: Molecular Scale Hydrophobicity and Adsorption Thermodynamics on Hydrophobic-Charged Surfaces
Source: ACS Nano. 2026 Feb 16;20(8):6970–82. doi: 10.1021/acsnano.5c18643 (PMC12961923; doi:10.1021/acsnano.5c18643)

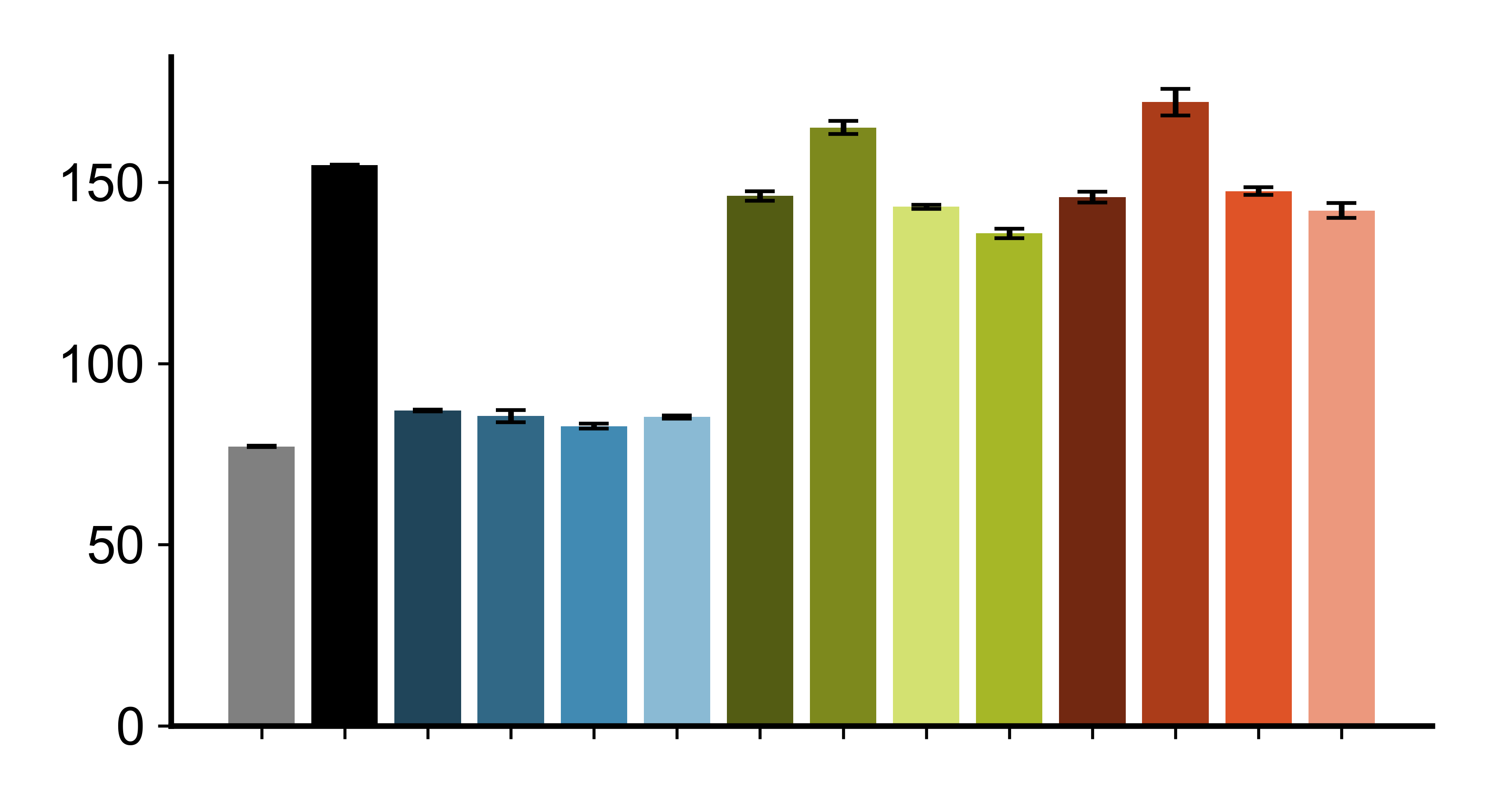

Supplement: Supplementary file 2 [file nn5c18643_si_002.zip › supplementary-files/linear-indus/free-energy.png]

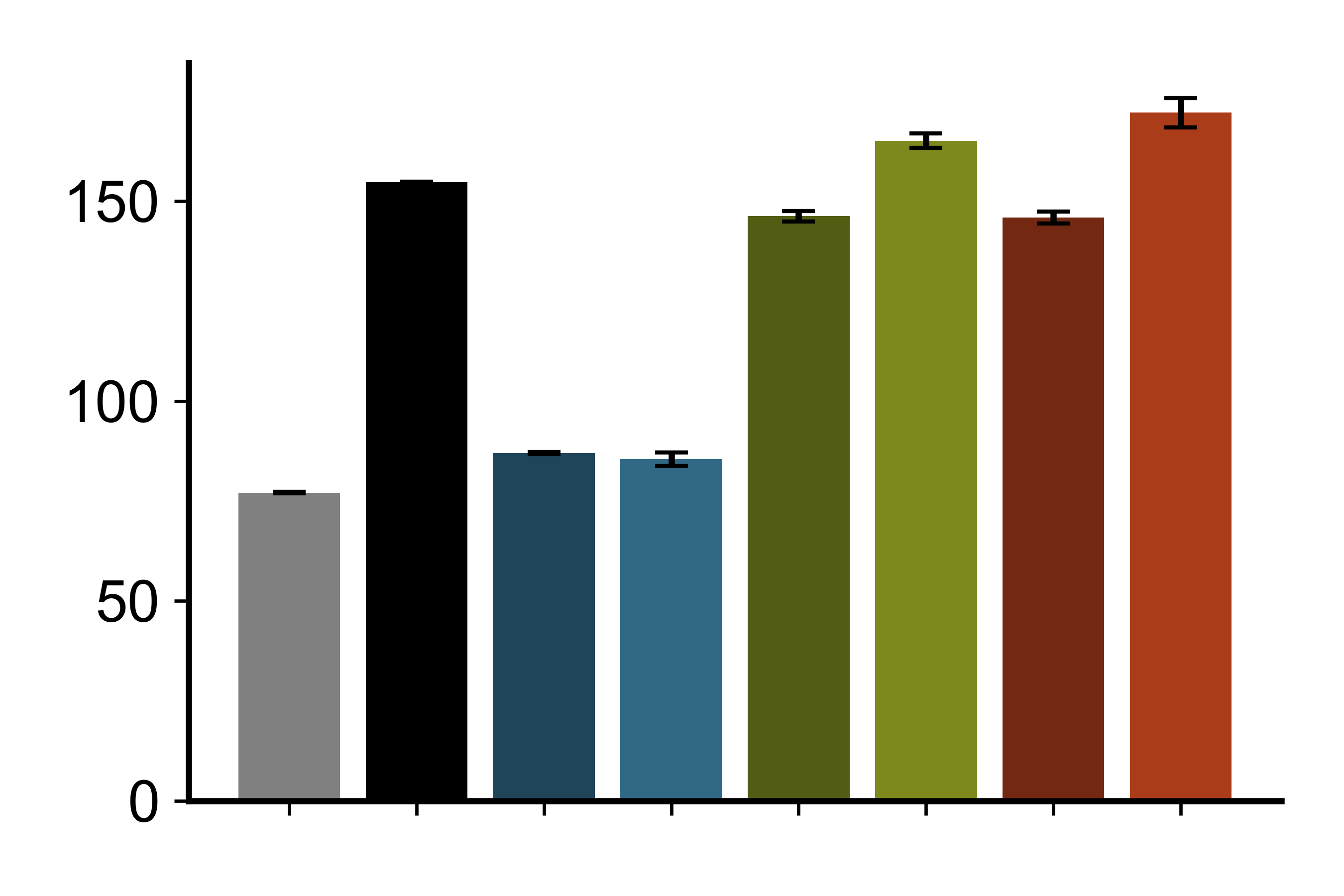

Supplement: Supplementary file 2 [file nn5c18643_si_002.zip › supplementary-files/linear-indus/free-energy-s0s1.png]

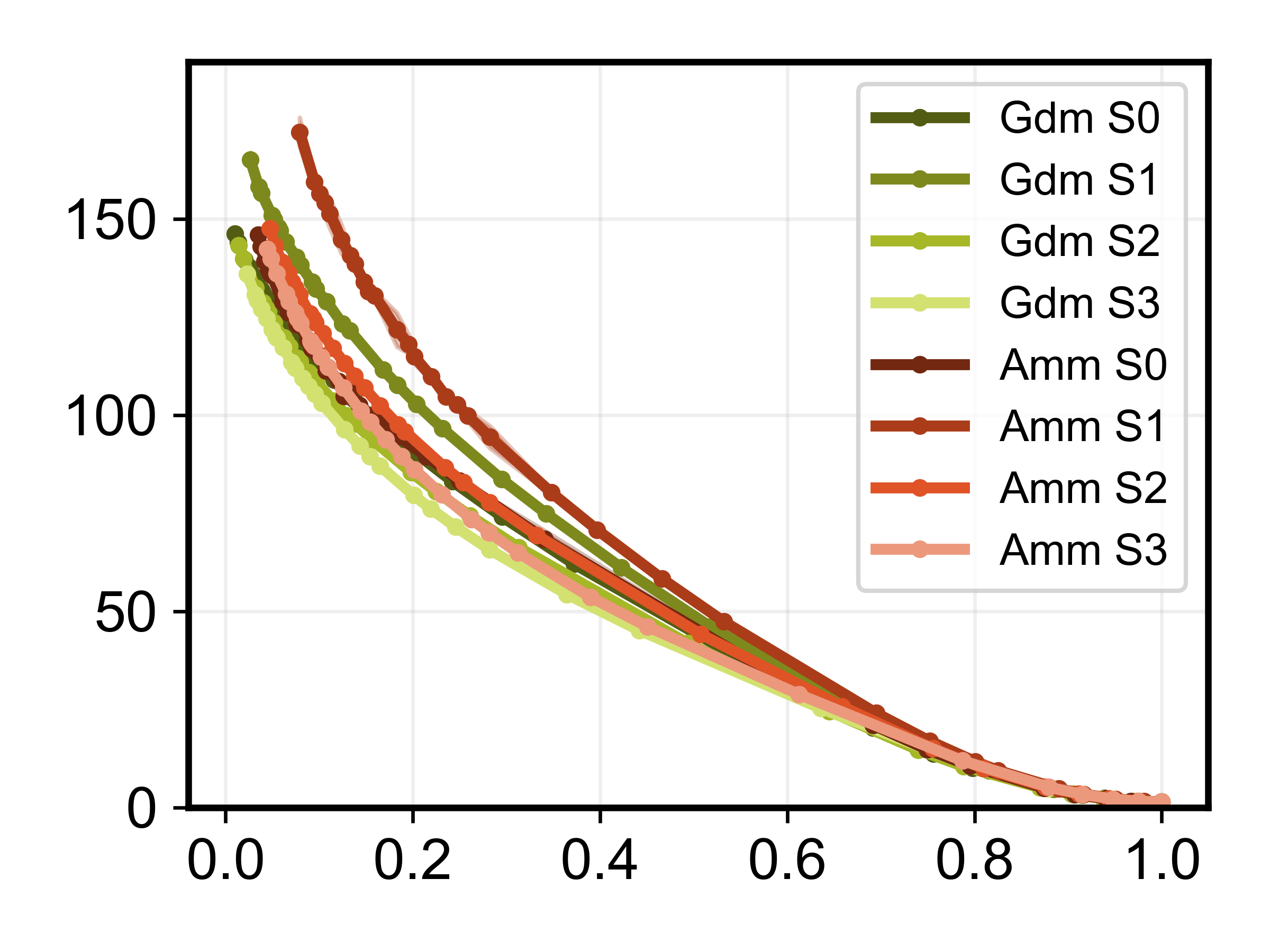

Supplement: Supplementary file 2 [file nn5c18643_si_002.zip › supplementary-files/linear-indus/indus-free-energy-charged.png]

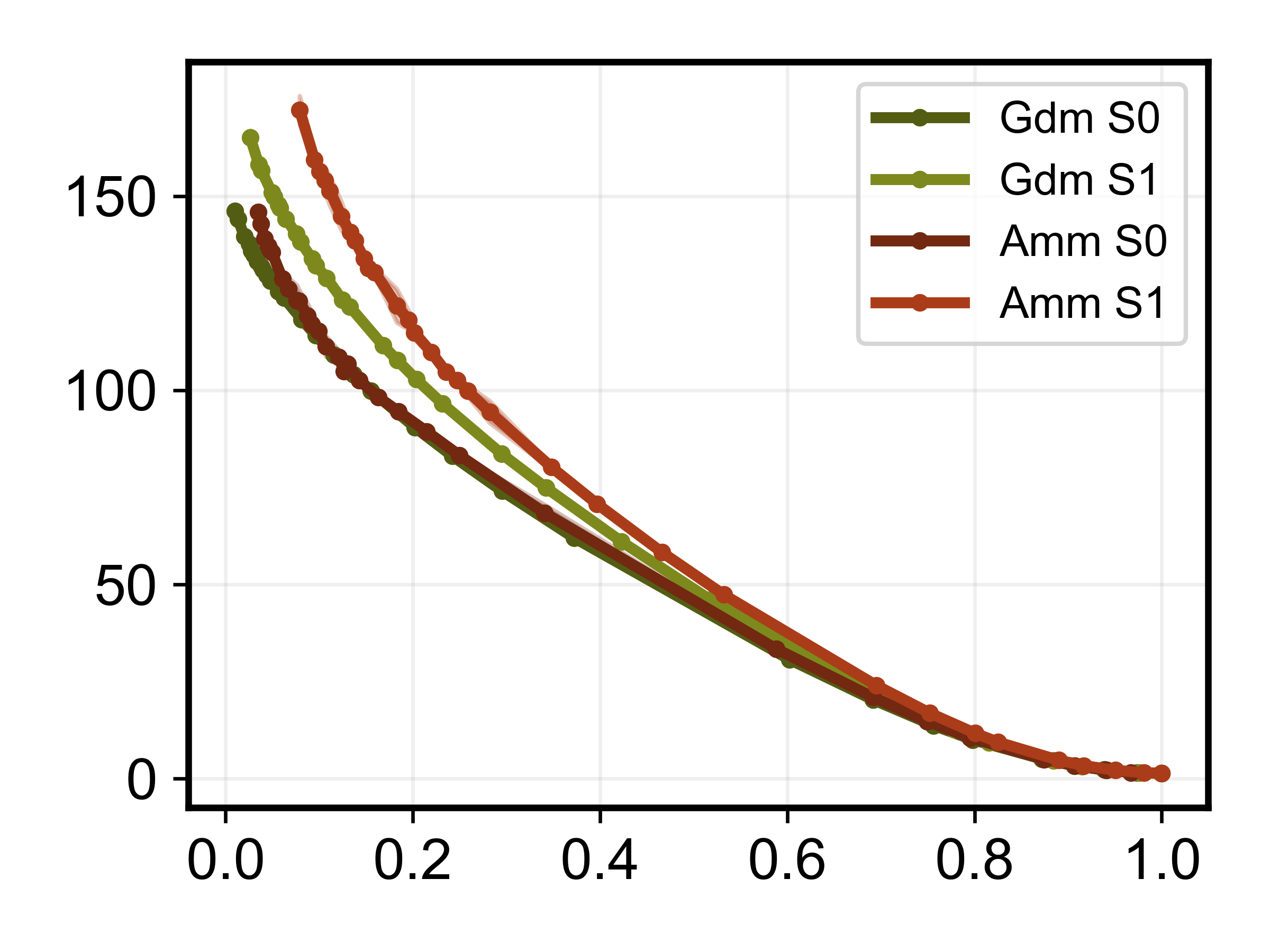

Supplement: Supplementary file 2 [file nn5c18643_si_002.zip › supplementary-files/linear-indus/indus-free-energy-charged-s0s1.png]

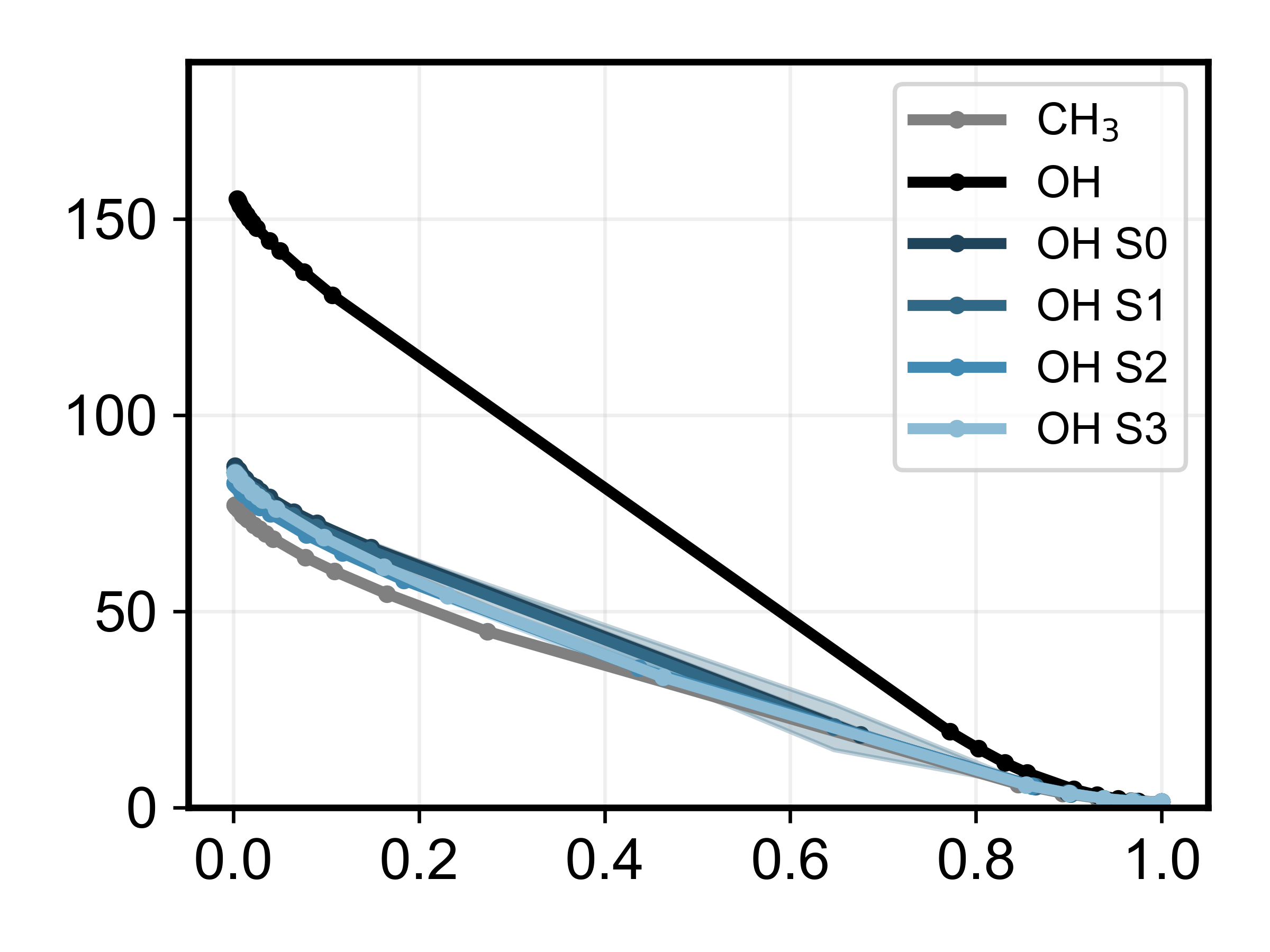

Supplement: Supplementary file 2 [file nn5c18643_si_002.zip › supplementary-files/linear-indus/indus-free-energy-uncharged.png]

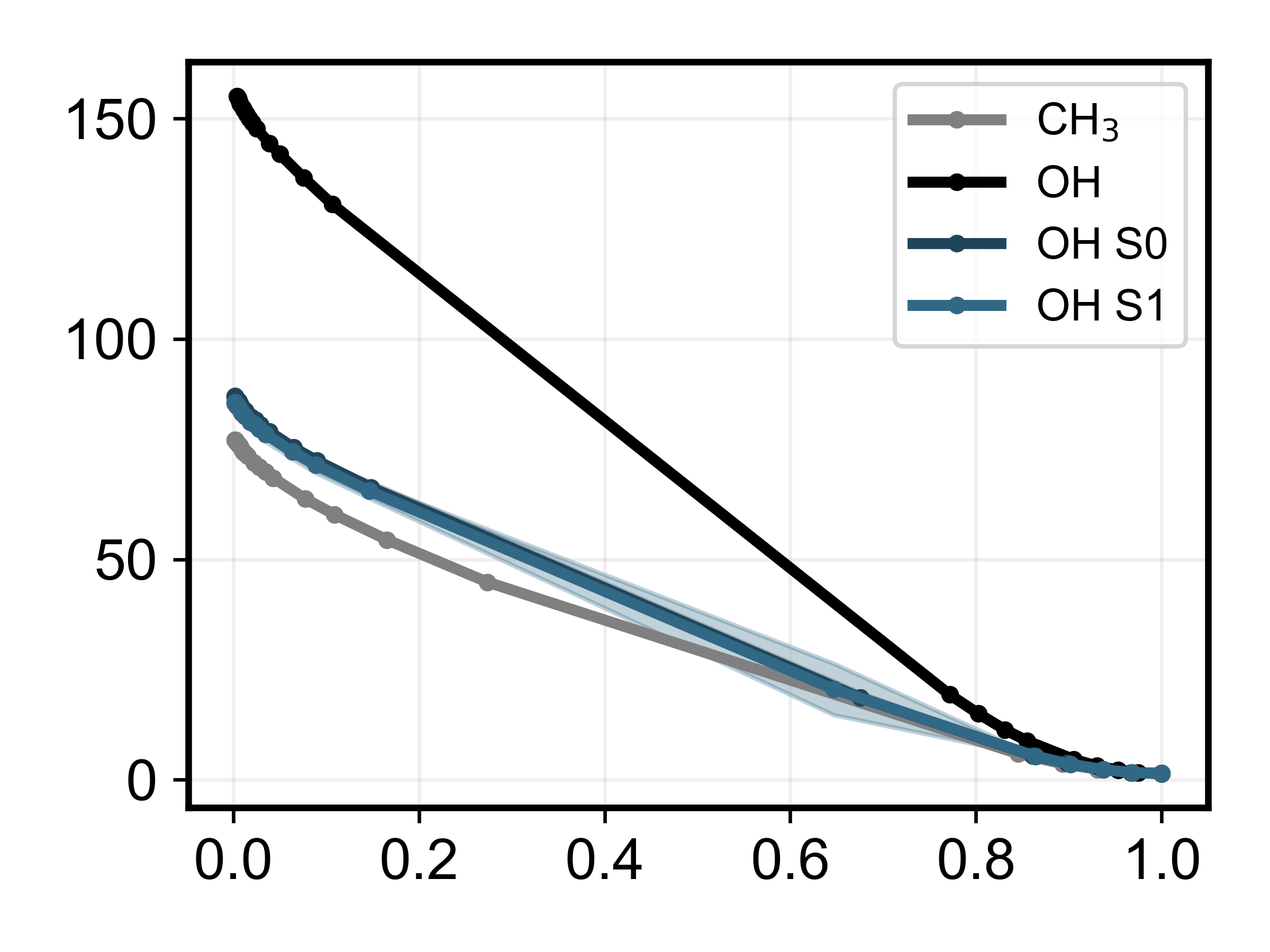

Supplement: Supplementary file 2 [file nn5c18643_si_002.zip › supplementary-files/linear-indus/indus-free-energy-uncharged-s0s1.png]

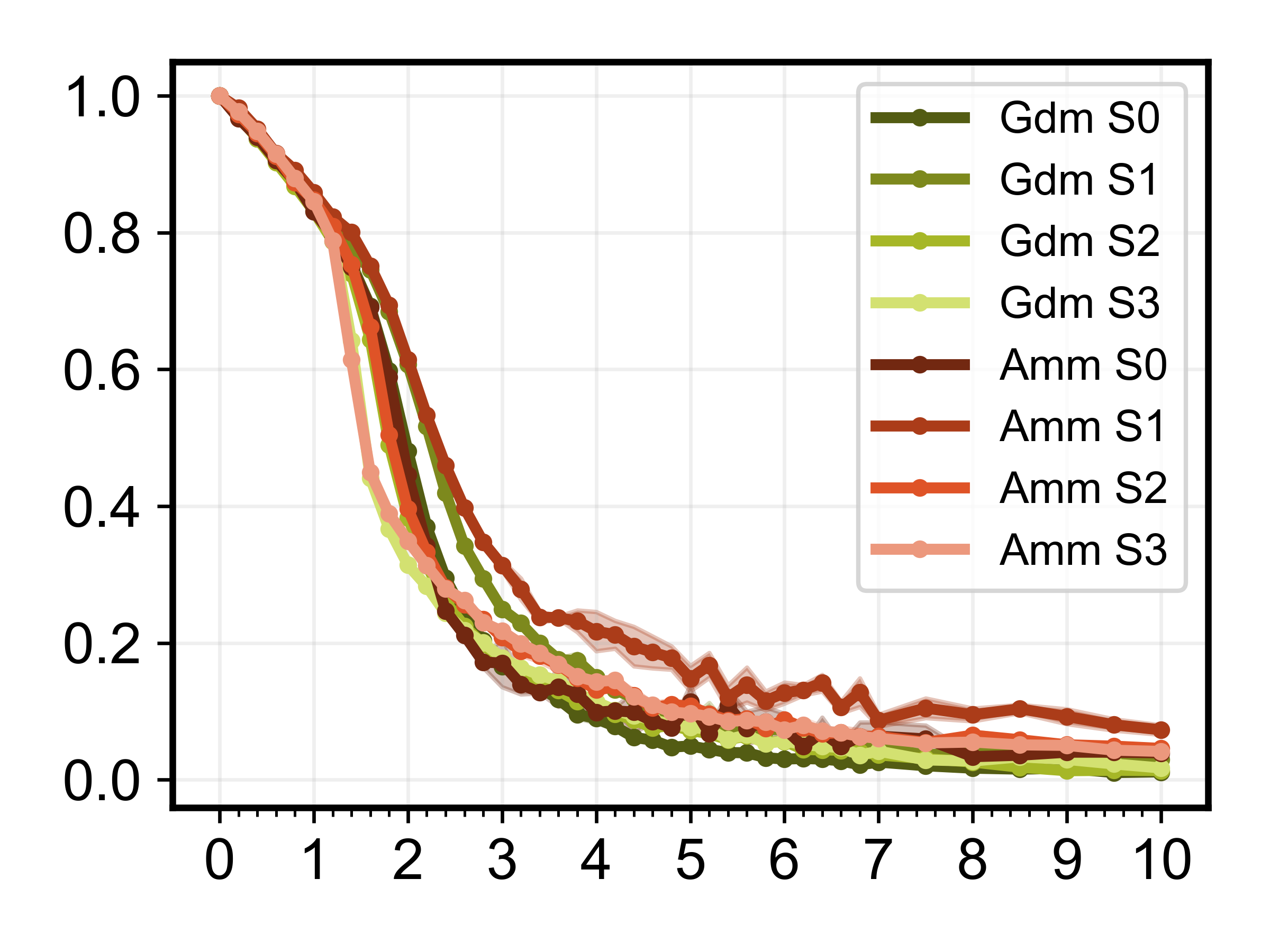

Supplement: Supplementary file 2 [file nn5c18643_si_002.zip › supplementary-files/linear-indus/linear-indus-charged.png]

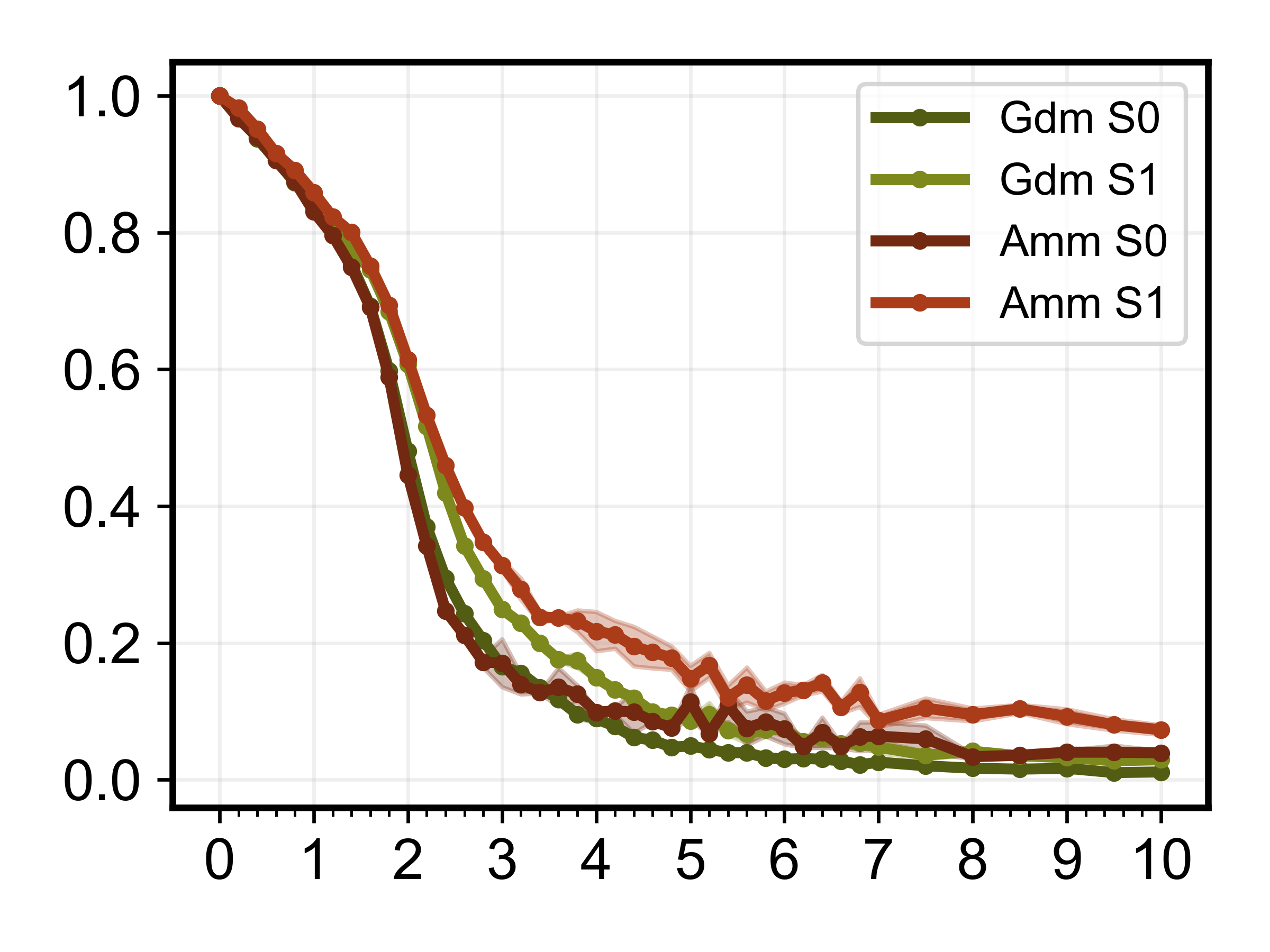

Supplement: Supplementary file 2 [file nn5c18643_si_002.zip › supplementary-files/linear-indus/linear-indus-charged-s0s1.png]

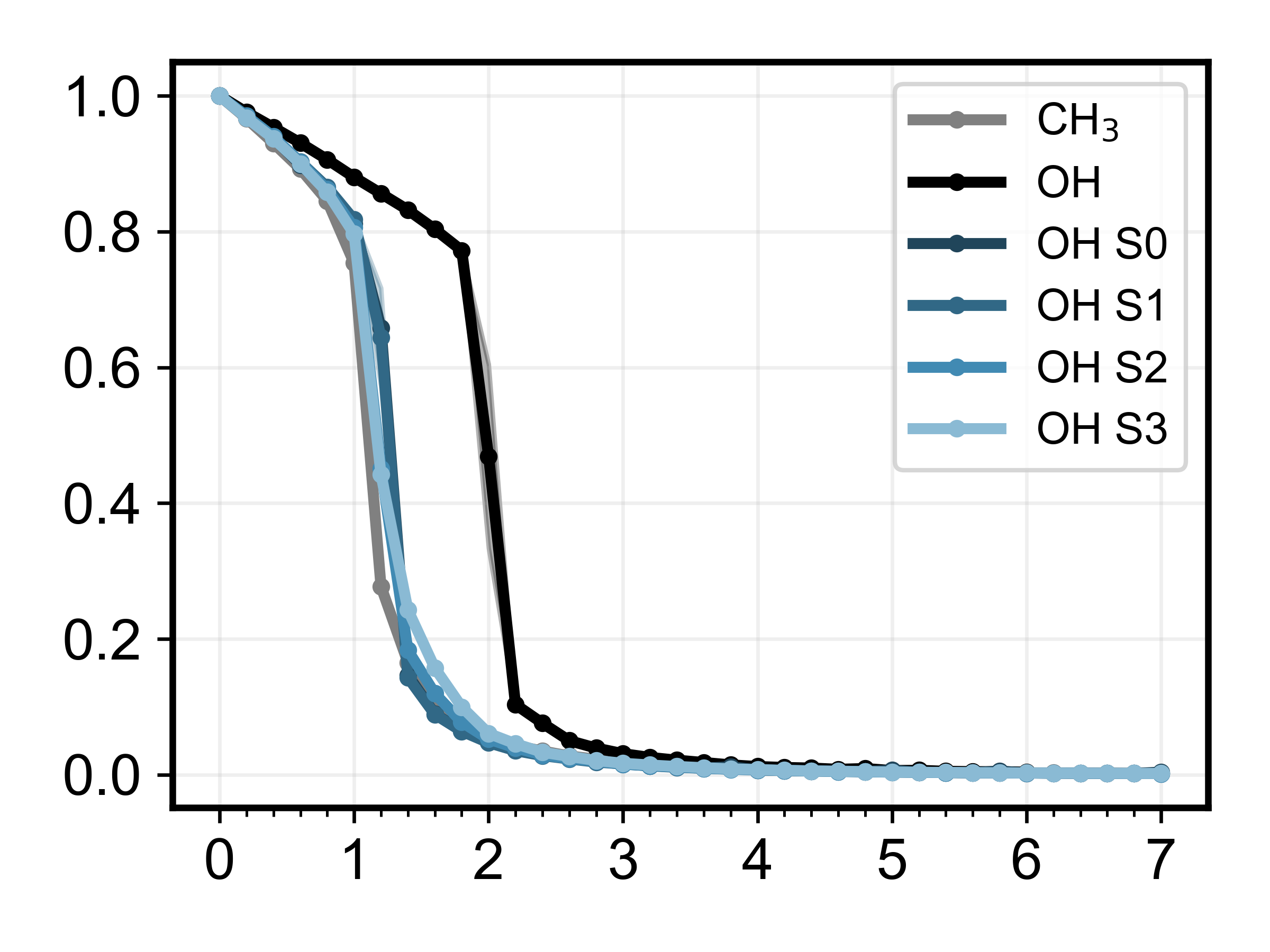

Supplement: Supplementary file 2 [file nn5c18643_si_002.zip › supplementary-files/linear-indus/linear-indus-uncharged.png]

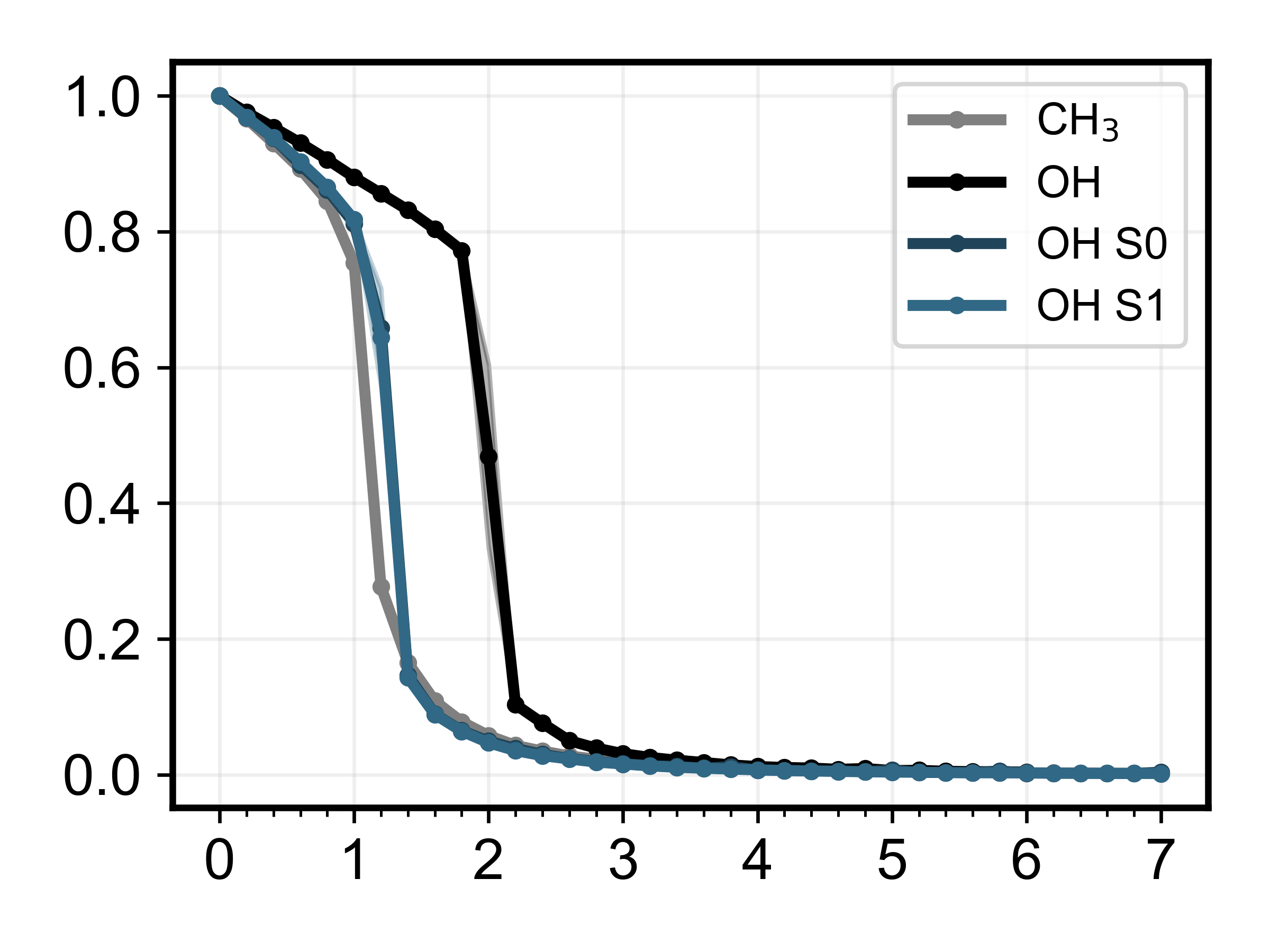

Supplement: Supplementary file 2 [file nn5c18643_si_002.zip › supplementary-files/linear-indus/linear-indus-uncharged-s0s1.png]

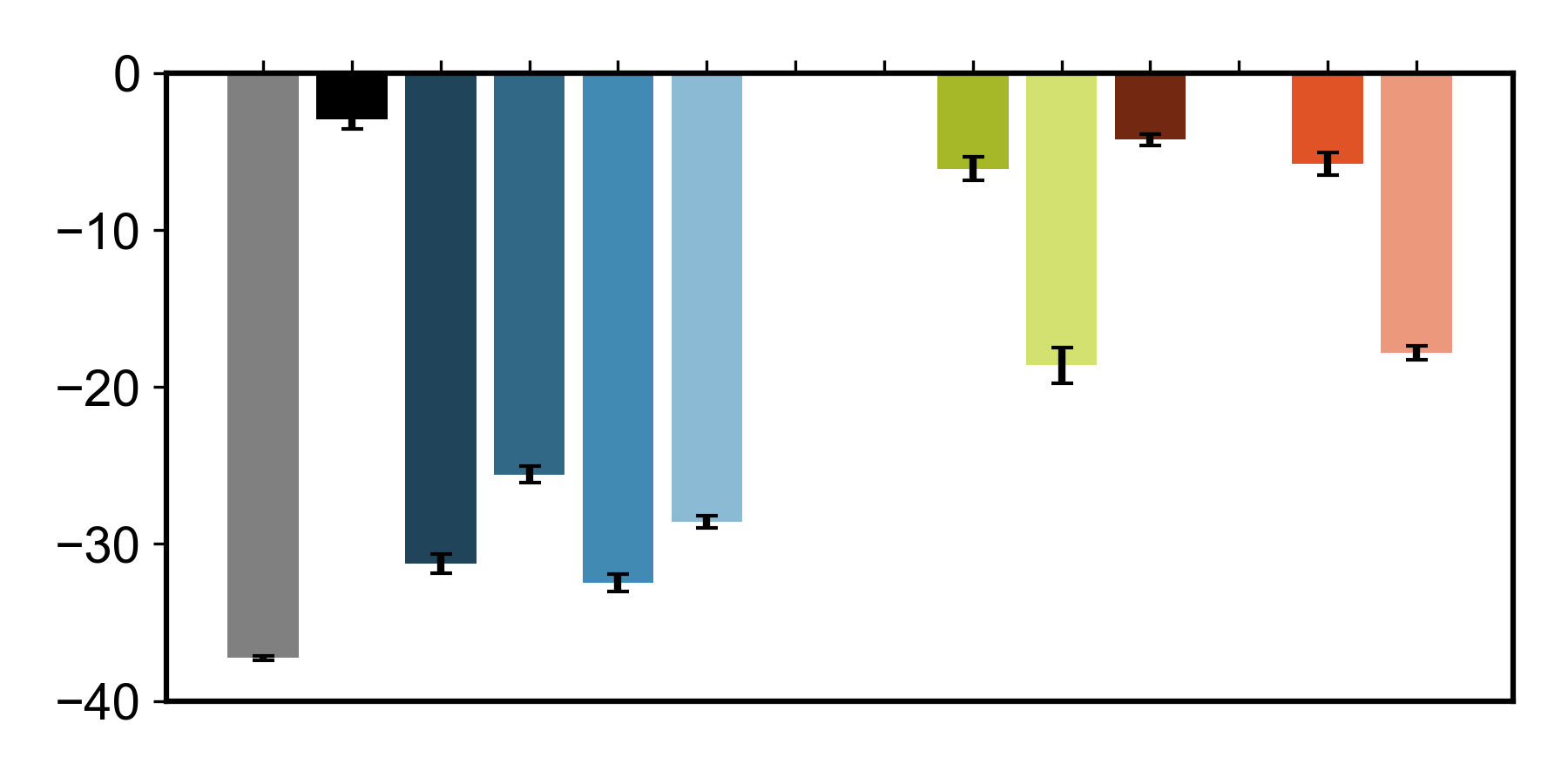

Supplement: Supplementary file 2 [file nn5c18643_si_002.zip › supplementary-files/pmf/all-2ns-equilibration/gnp/final-results/delg-all.png]

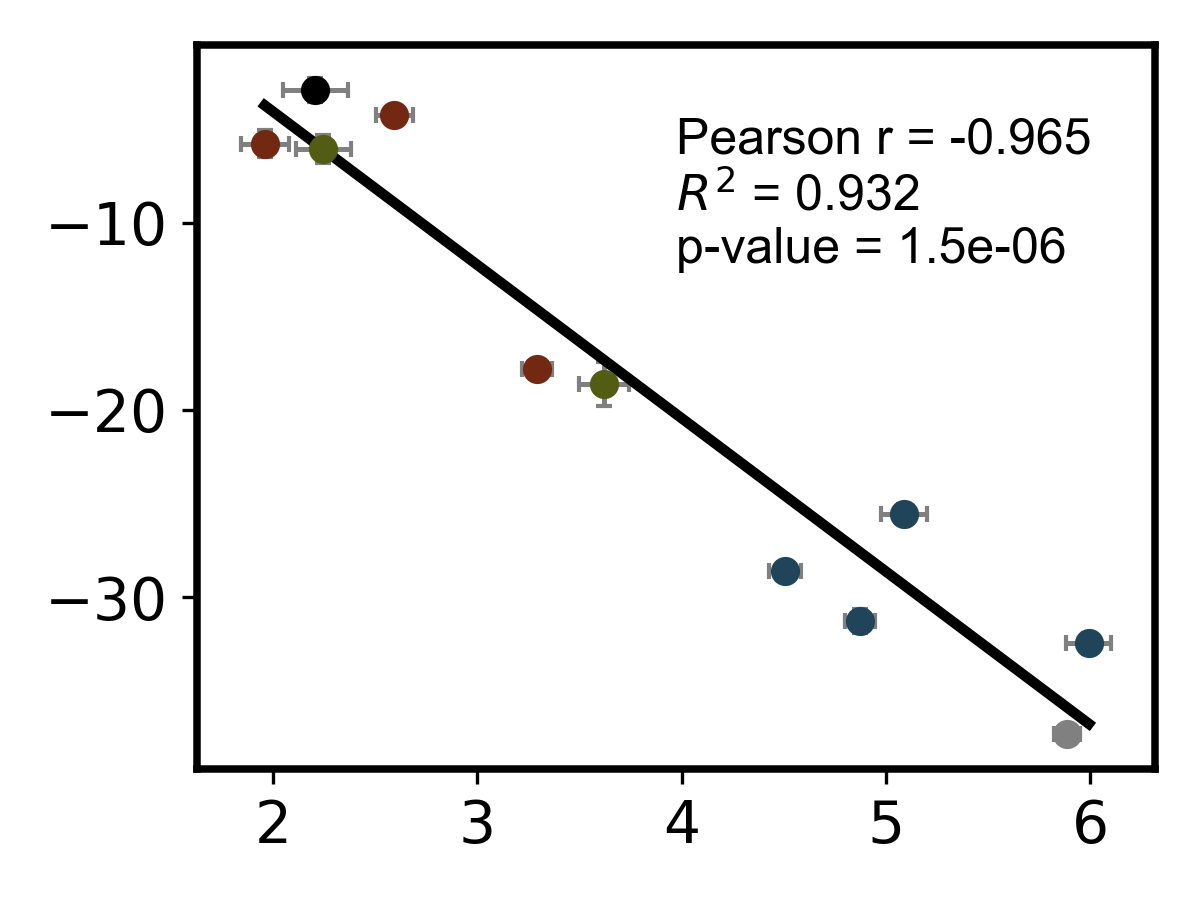

Supplement: Supplementary file 2 [file nn5c18643_si_002.zip › supplementary-files/pmf/all-2ns-equilibration/gnp/final-results/delg-vs-area.png]

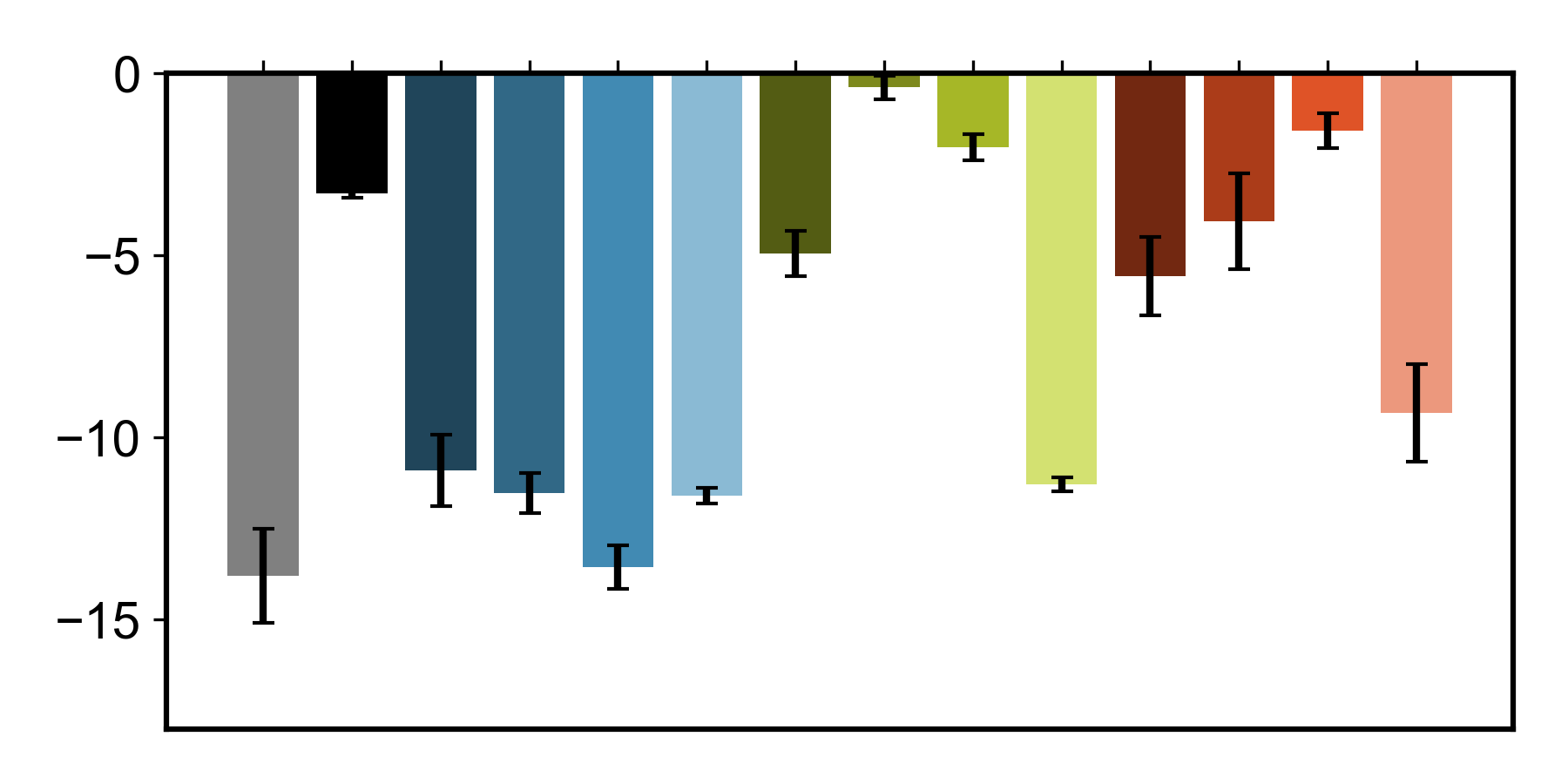

Supplement: Supplementary file 2 [file nn5c18643_si_002.zip › supplementary-files/pmf/all-2ns-equilibration/hydrophobin/final-results/delg-all.png]

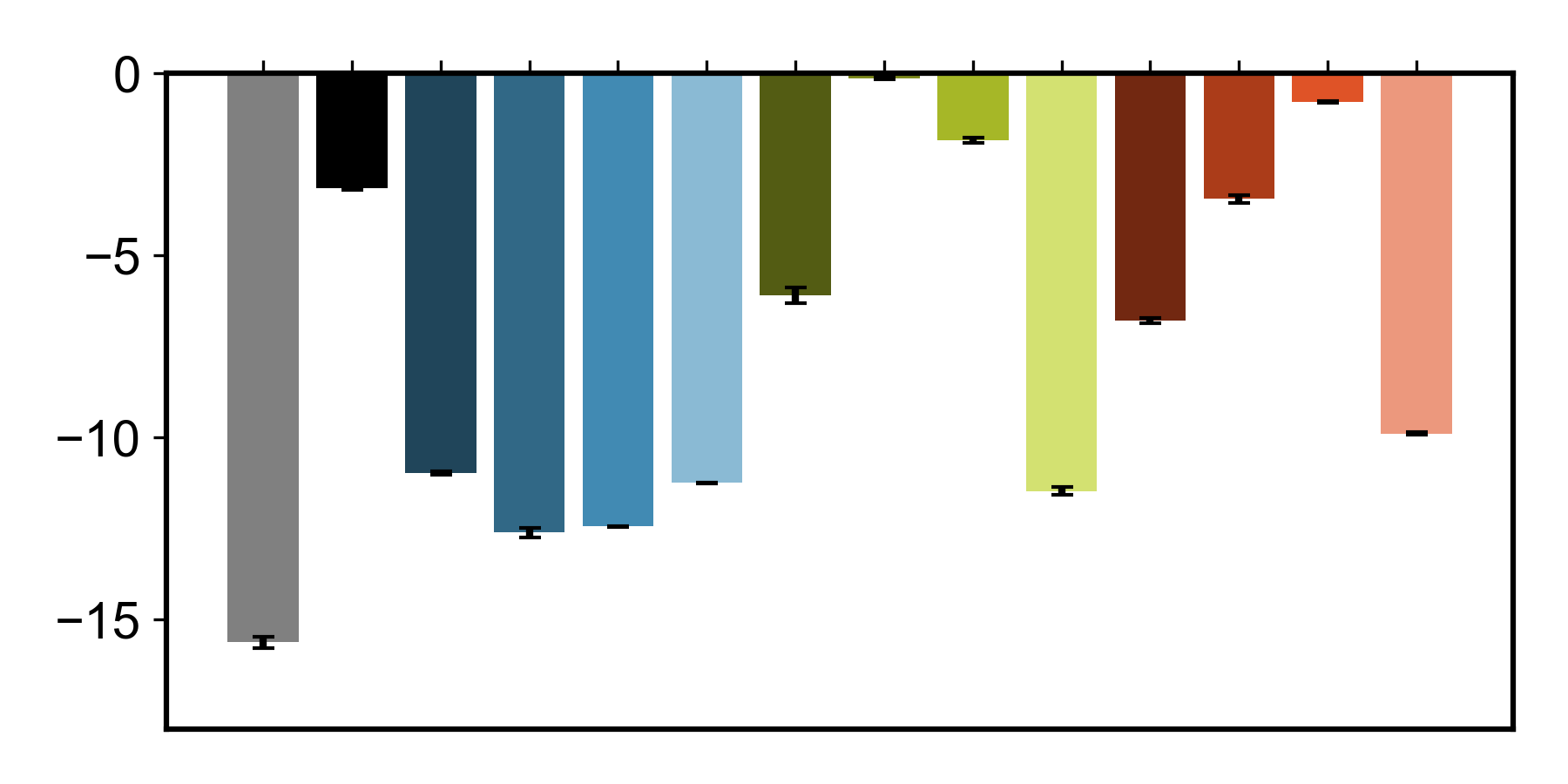

Supplement: Supplementary file 2 [file nn5c18643_si_002.zip › supplementary-files/pmf/all-2ns-equilibration/hydrophobin/final-results/delg-all-simulation1.png]

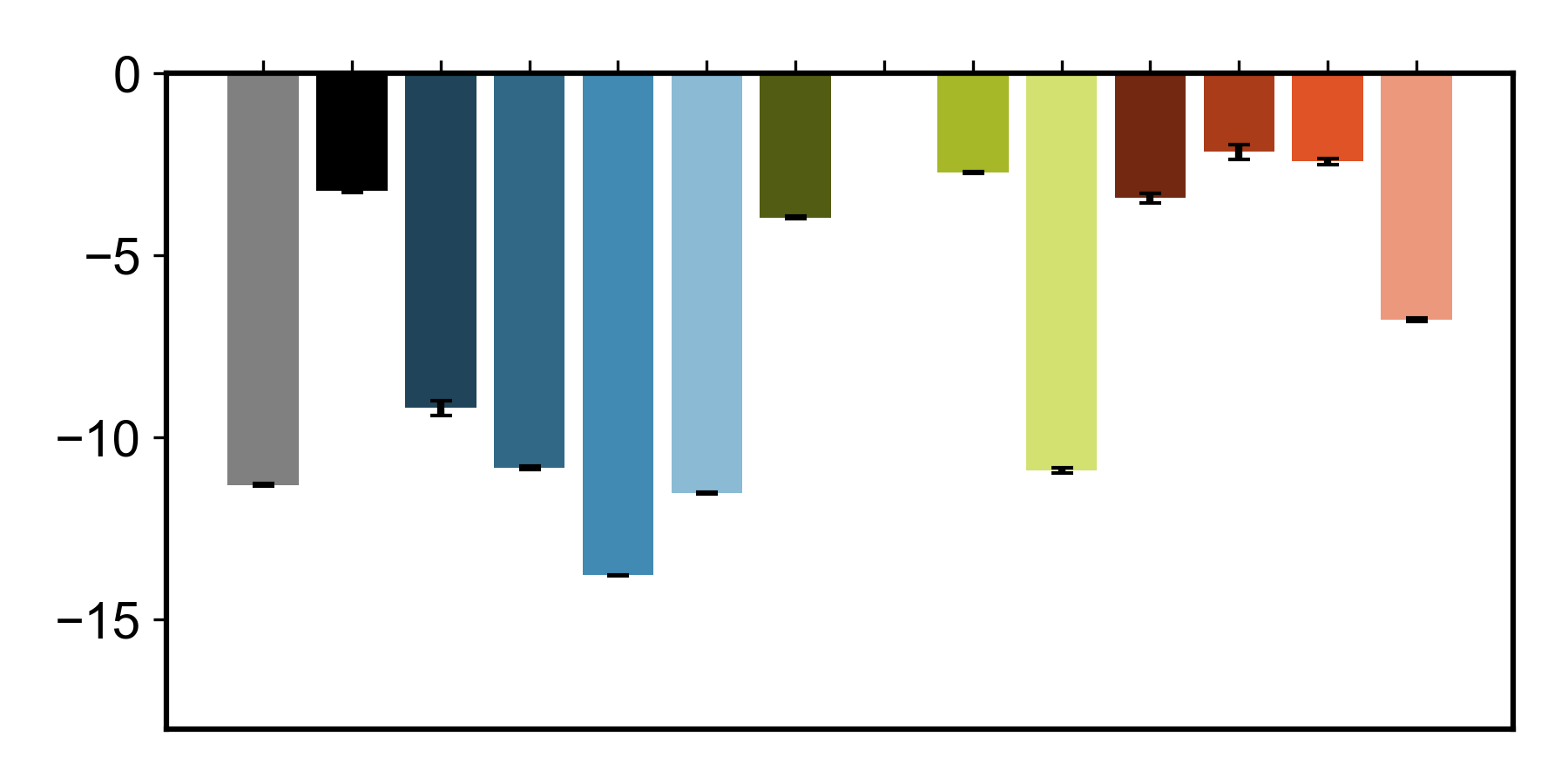

Supplement: Supplementary file 2 [file nn5c18643_si_002.zip › supplementary-files/pmf/all-2ns-equilibration/hydrophobin/final-results/delg-all-simulation2.png]

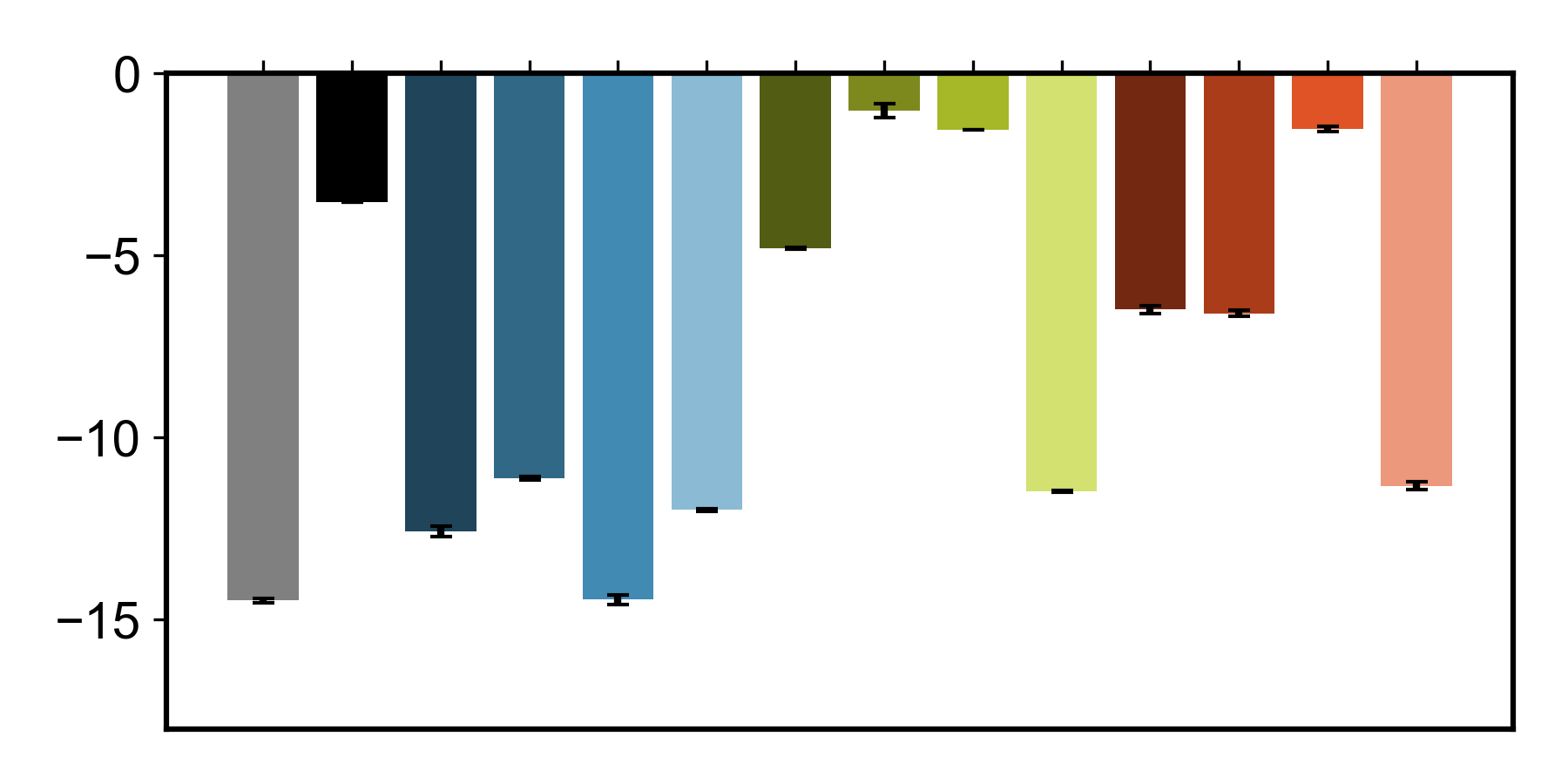

Supplement: Supplementary file 2 [file nn5c18643_si_002.zip › supplementary-files/pmf/all-2ns-equilibration/hydrophobin/final-results/delg-all-simulation3.png]

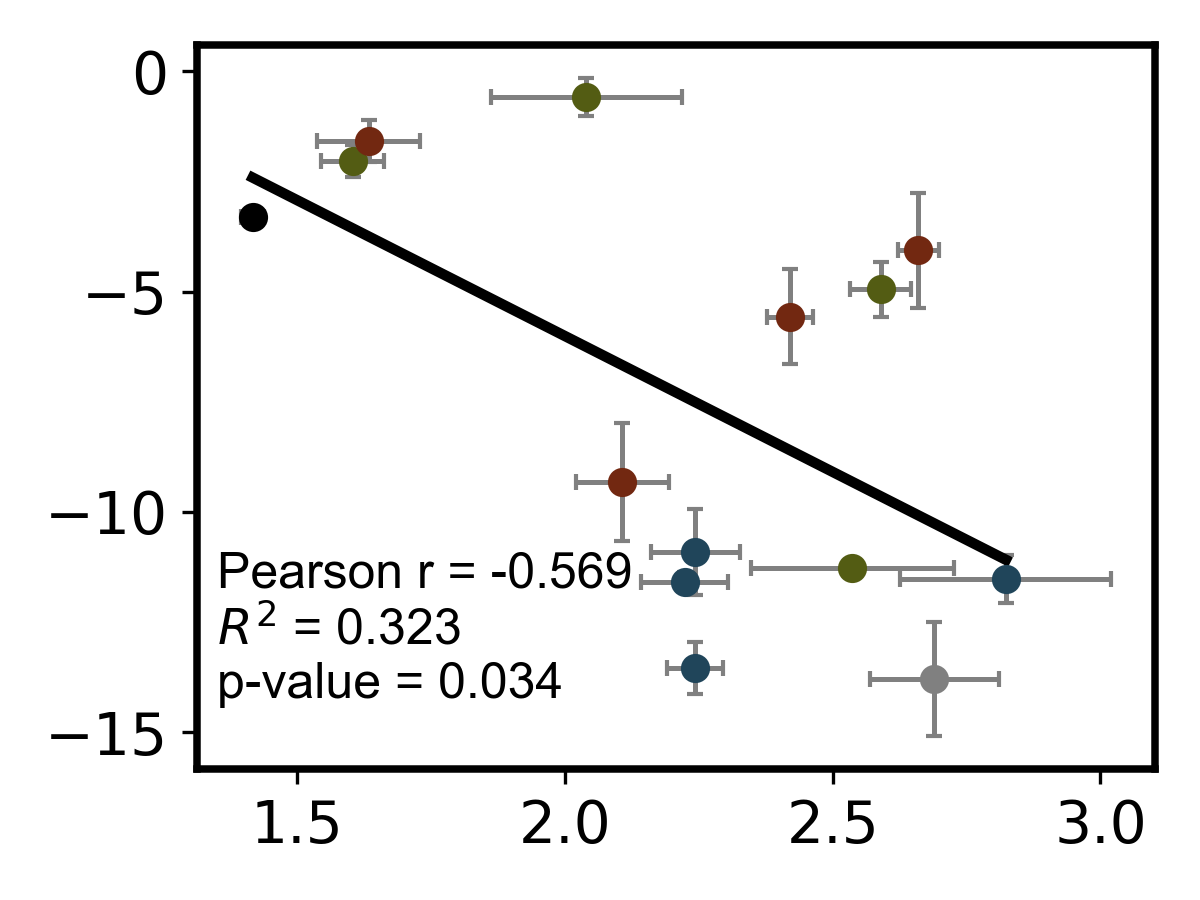

Supplement: Supplementary file 2 [file nn5c18643_si_002.zip › supplementary-files/pmf/all-2ns-equilibration/hydrophobin/final-results/delg-vs-area.png]

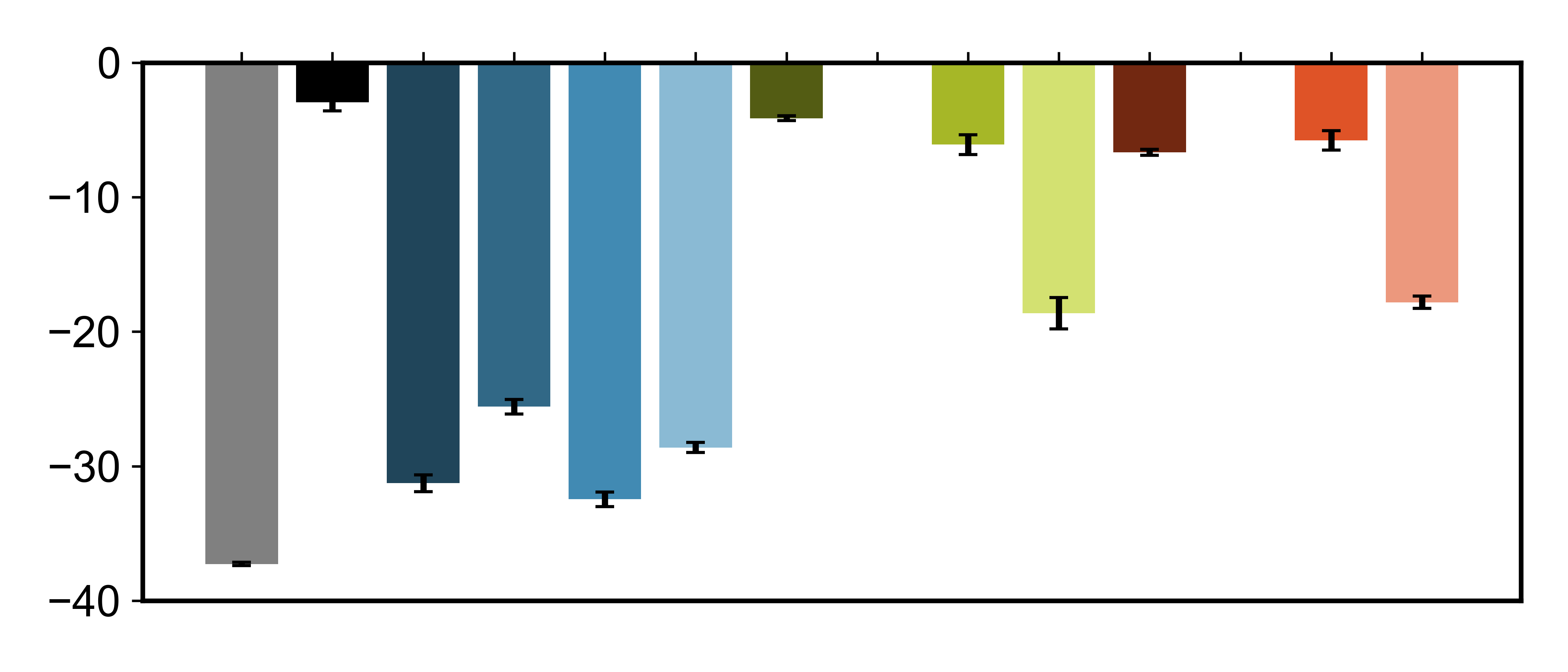

Supplement: Supplementary file 2 [file nn5c18643_si_002.zip › supplementary-files/pmf/reported-equilibration/gnp/delg-all-final.png]

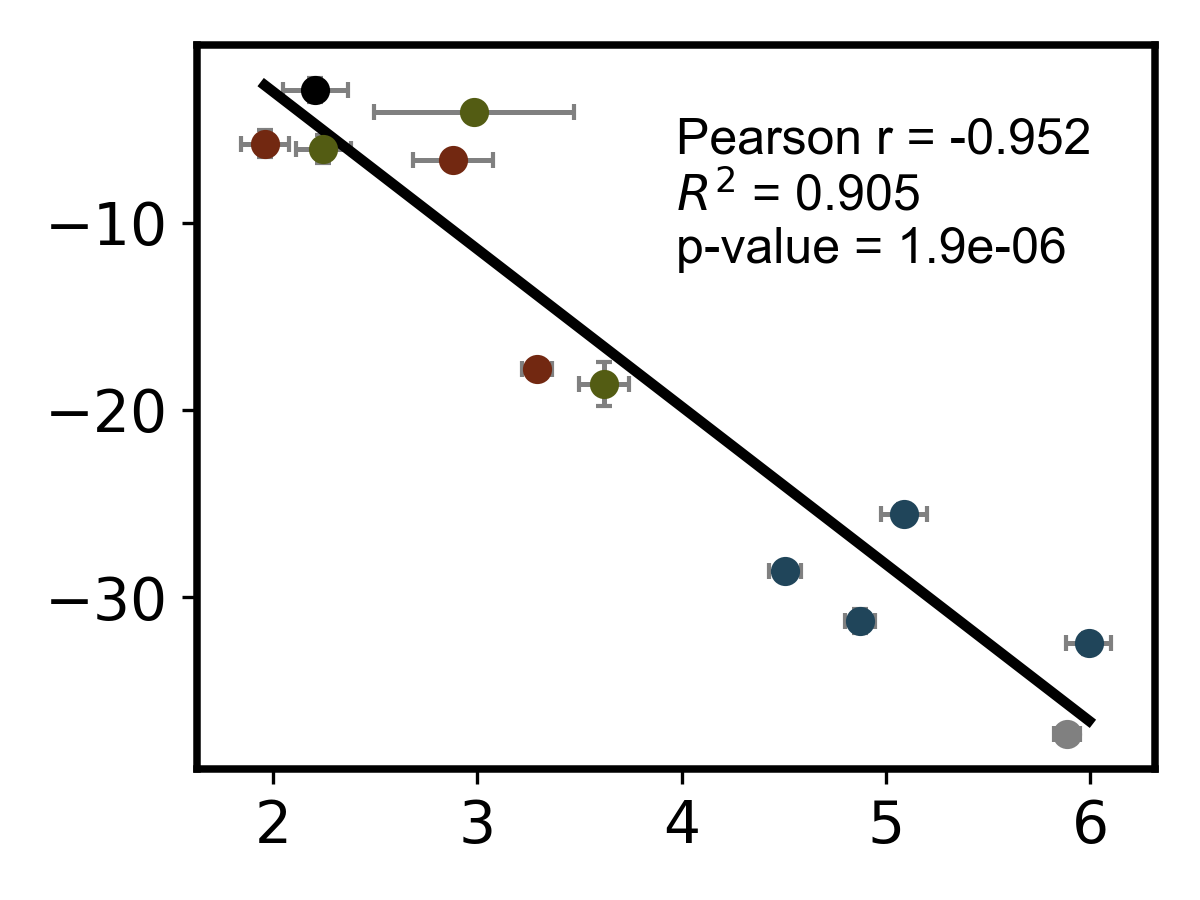

Supplement: Supplementary file 2 [file nn5c18643_si_002.zip › supplementary-files/pmf/reported-equilibration/gnp/delg-vs-area.png]

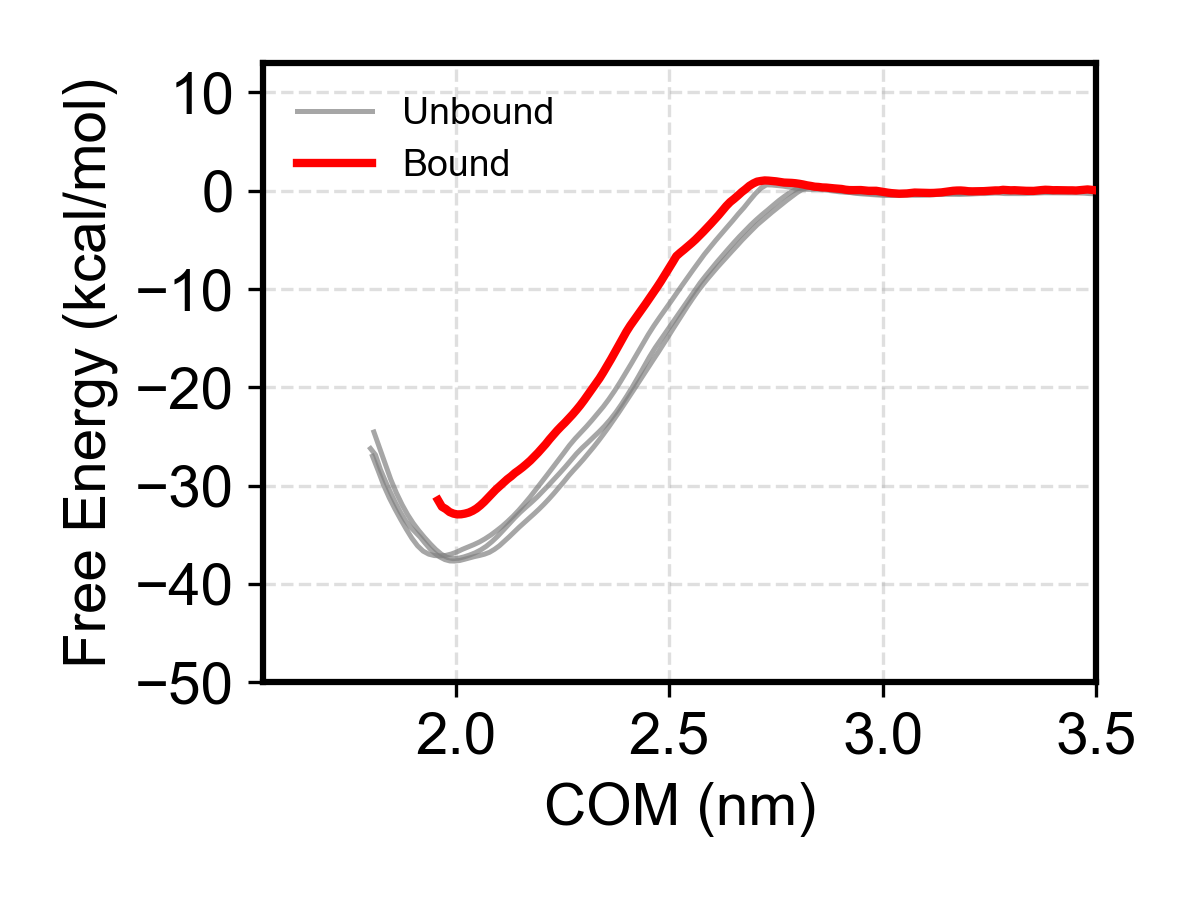

Supplement: Supplementary file 2 [file nn5c18643_si_002.zip › supplementary-files/pmf/reported-equilibration/gnp/plot-pmf/pmf-hb-ch3_100ns.png]

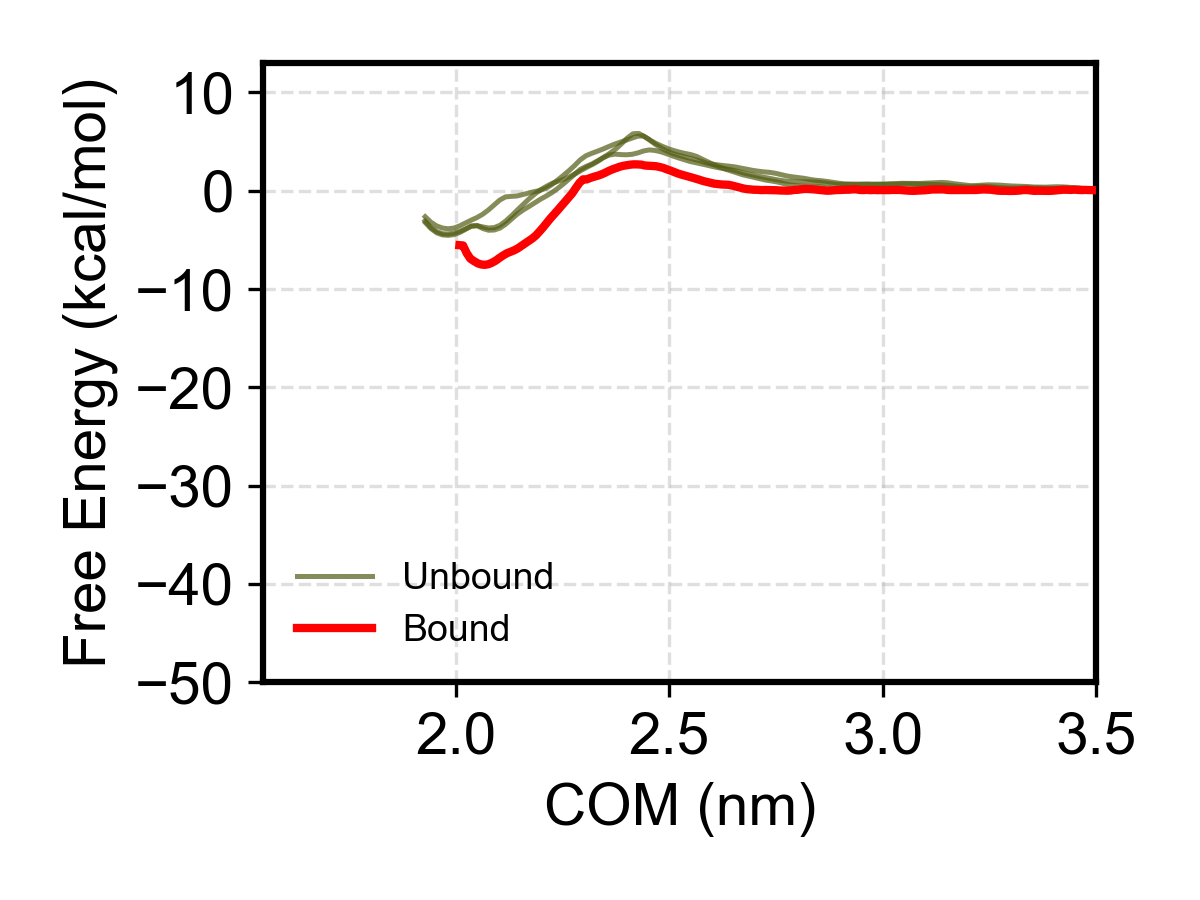

Supplement: Supplementary file 2 [file nn5c18643_si_002.zip › supplementary-files/pmf/reported-equilibration/gnp/plot-pmf/pmf-hb-gms0_100ns.png]

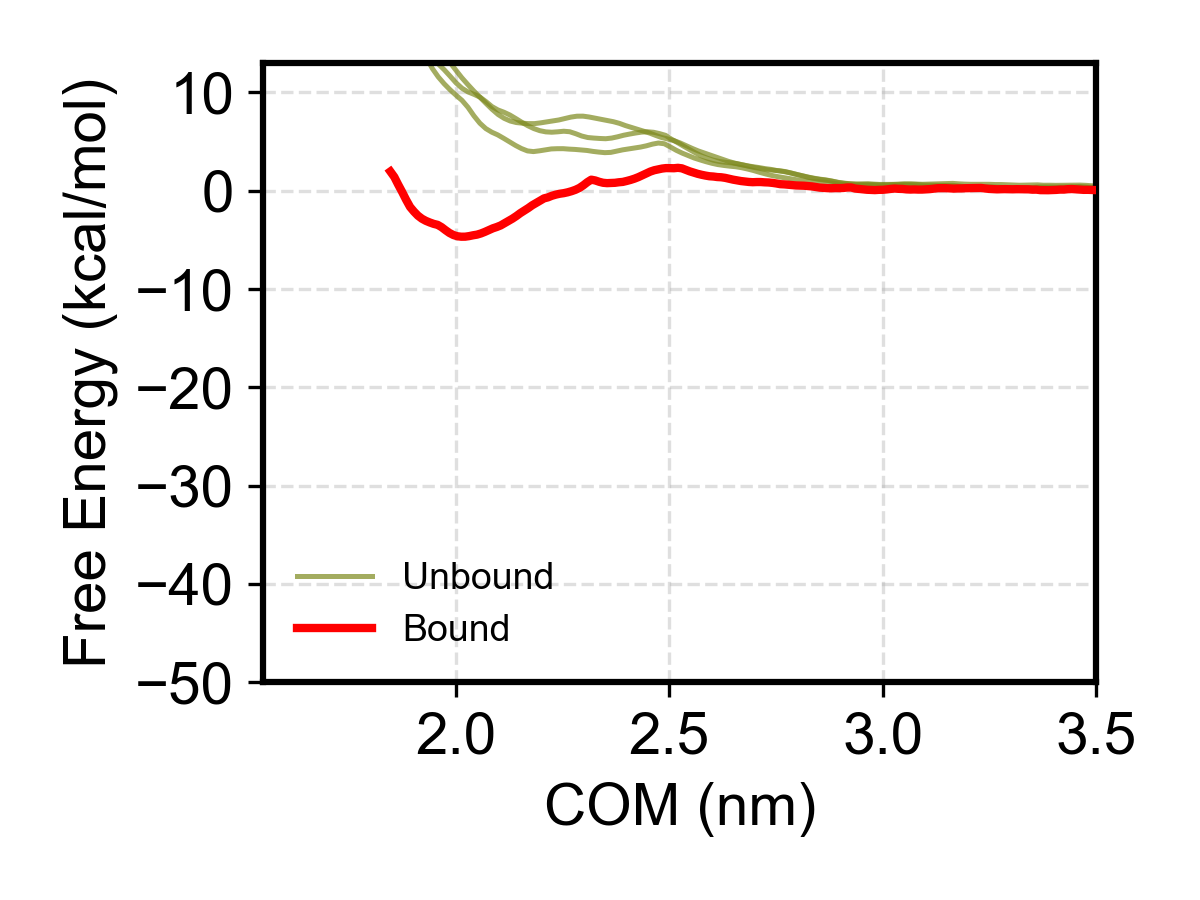

Supplement: Supplementary file 2 [file nn5c18643_si_002.zip › supplementary-files/pmf/reported-equilibration/gnp/plot-pmf/pmf-hb-gms1_100ns.png]

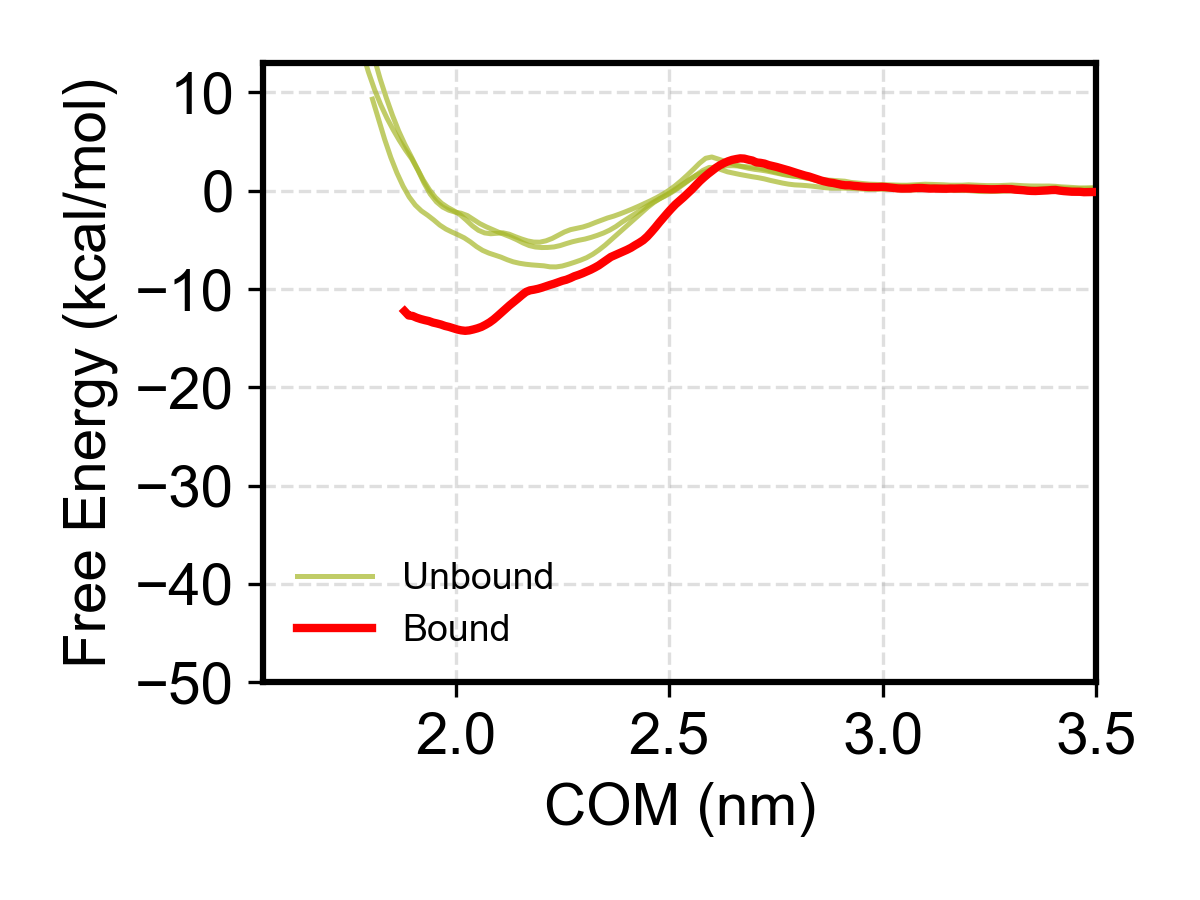

Supplement: Supplementary file 2 [file nn5c18643_si_002.zip › supplementary-files/pmf/reported-equilibration/gnp/plot-pmf/pmf-hb-gms2_100ns.png]

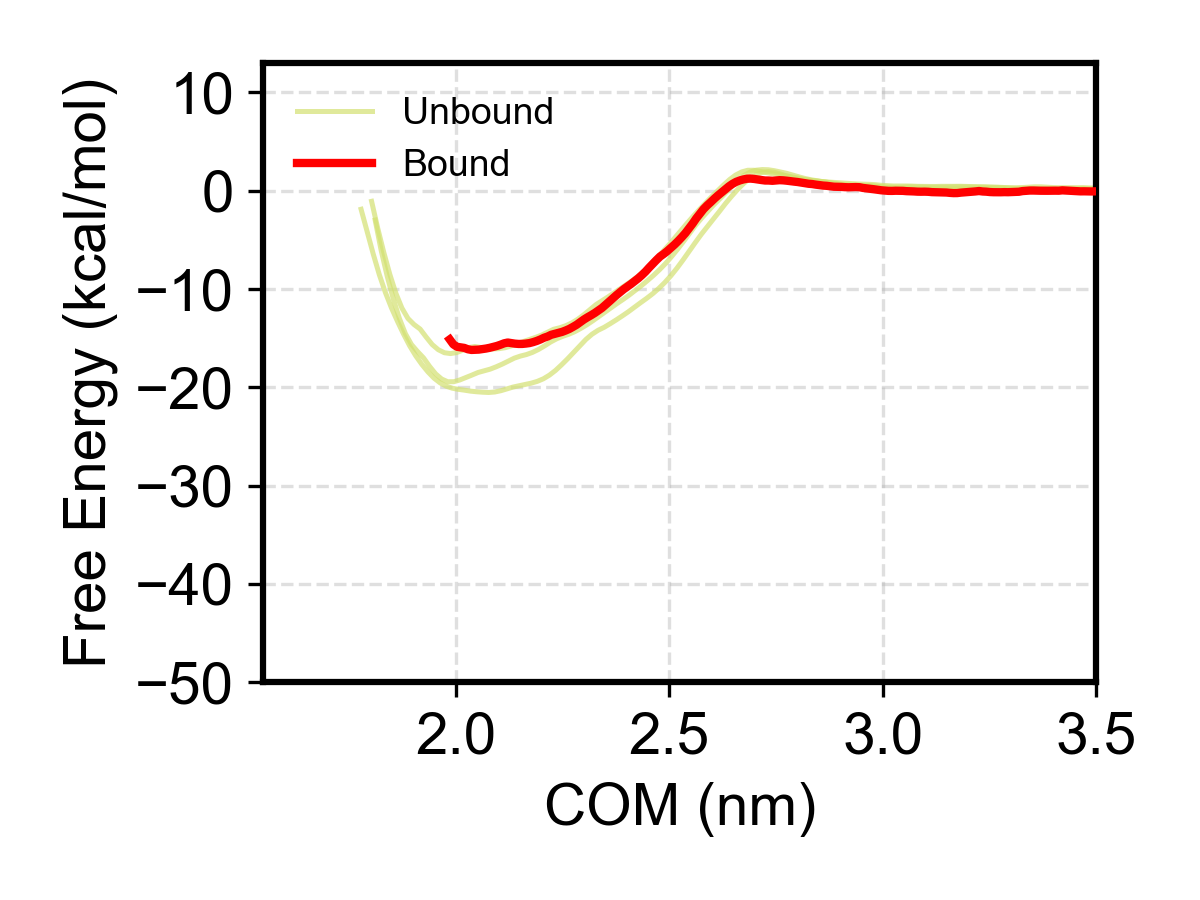

Supplement: Supplementary file 2 [file nn5c18643_si_002.zip › supplementary-files/pmf/reported-equilibration/gnp/plot-pmf/pmf-hb-gms3_100ns.png]

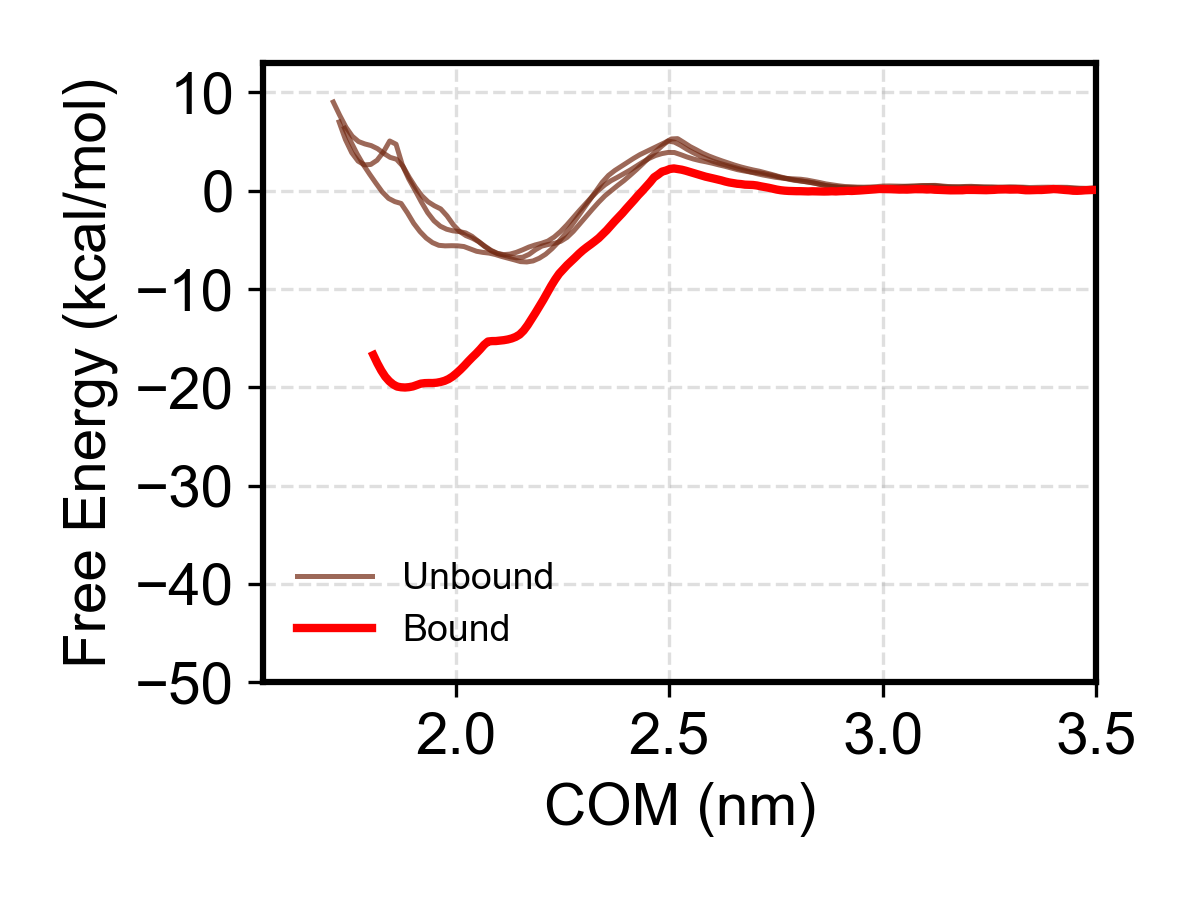

Supplement: Supplementary file 2 [file nn5c18643_si_002.zip › supplementary-files/pmf/reported-equilibration/gnp/plot-pmf/pmf-hb-nh3s0_100ns.png]

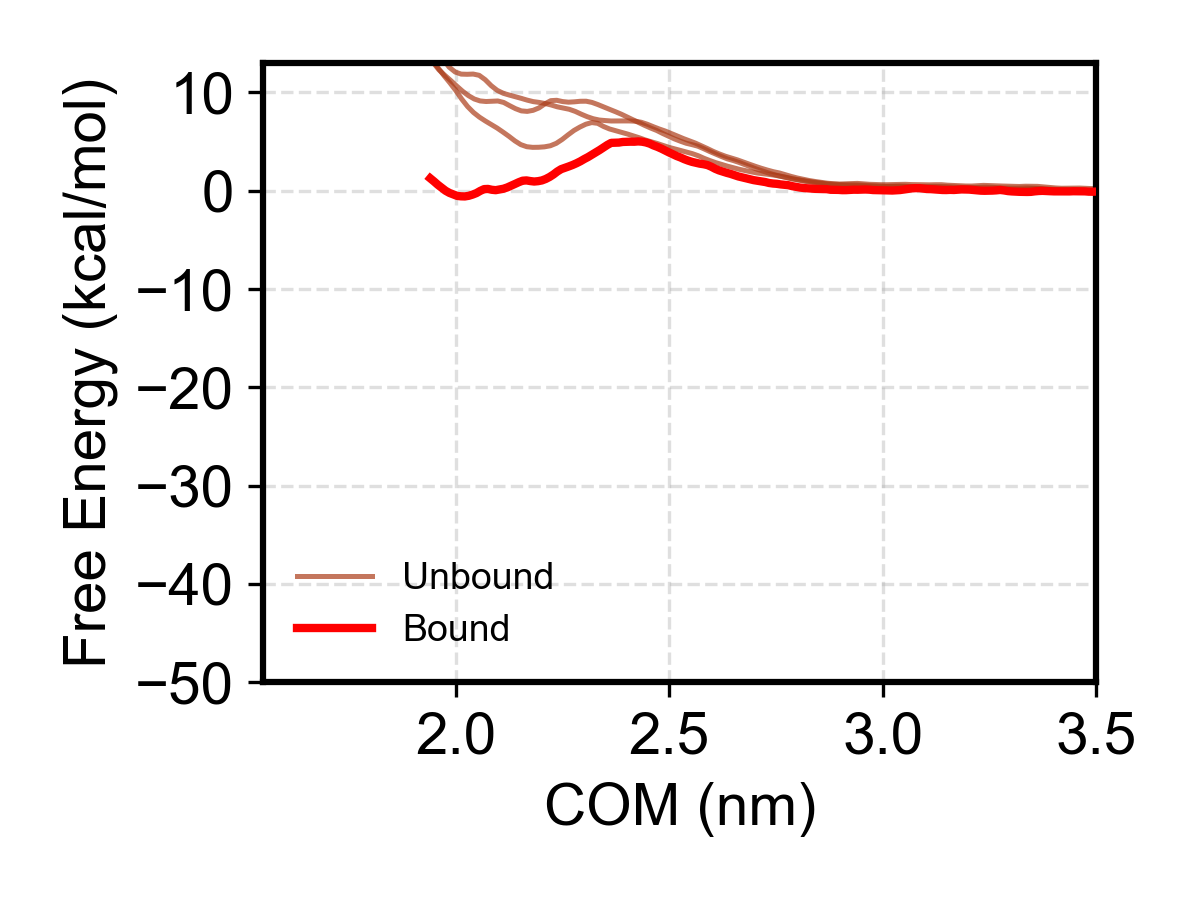

Supplement: Supplementary file 2 [file nn5c18643_si_002.zip › supplementary-files/pmf/reported-equilibration/gnp/plot-pmf/pmf-hb-nh3s1_100ns.png]

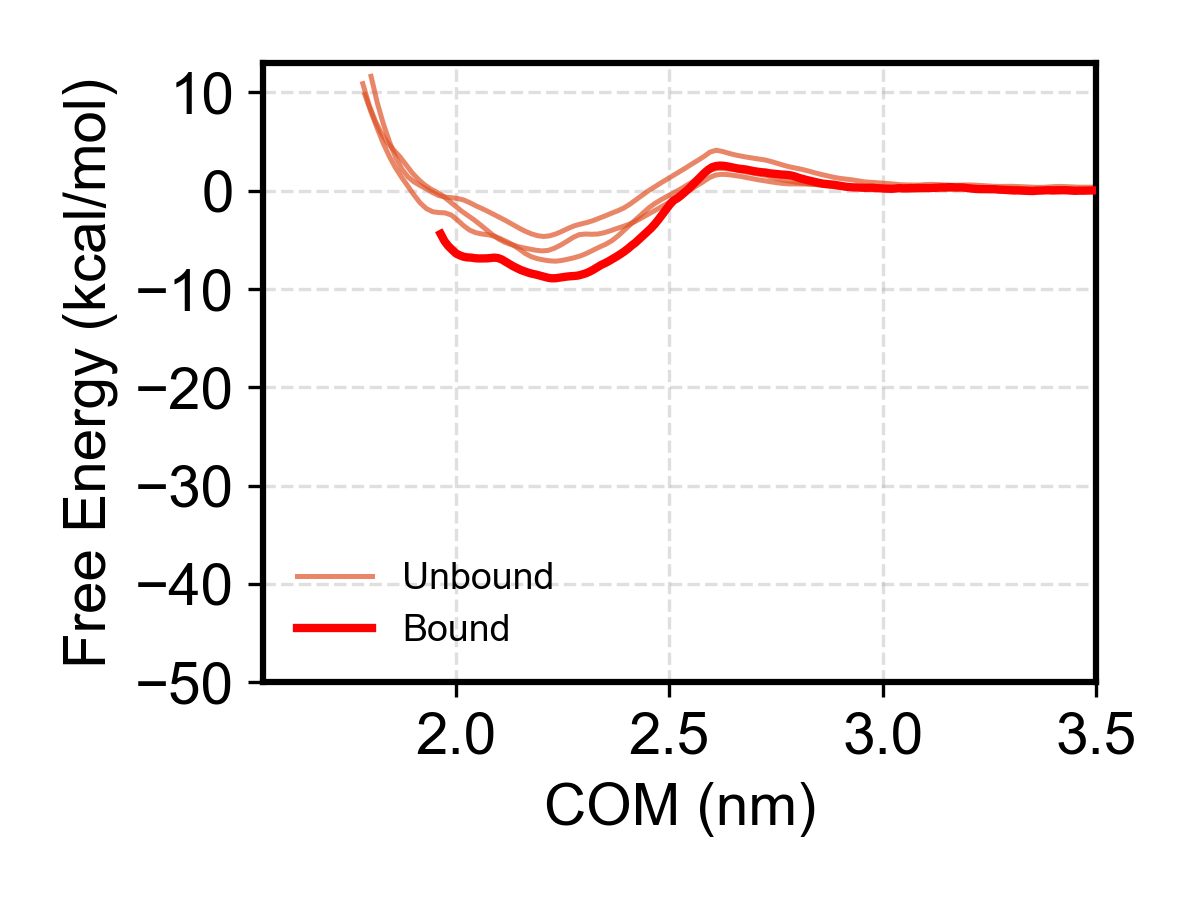

Supplement: Supplementary file 2 [file nn5c18643_si_002.zip › supplementary-files/pmf/reported-equilibration/gnp/plot-pmf/pmf-hb-nh3s2_100ns.png]

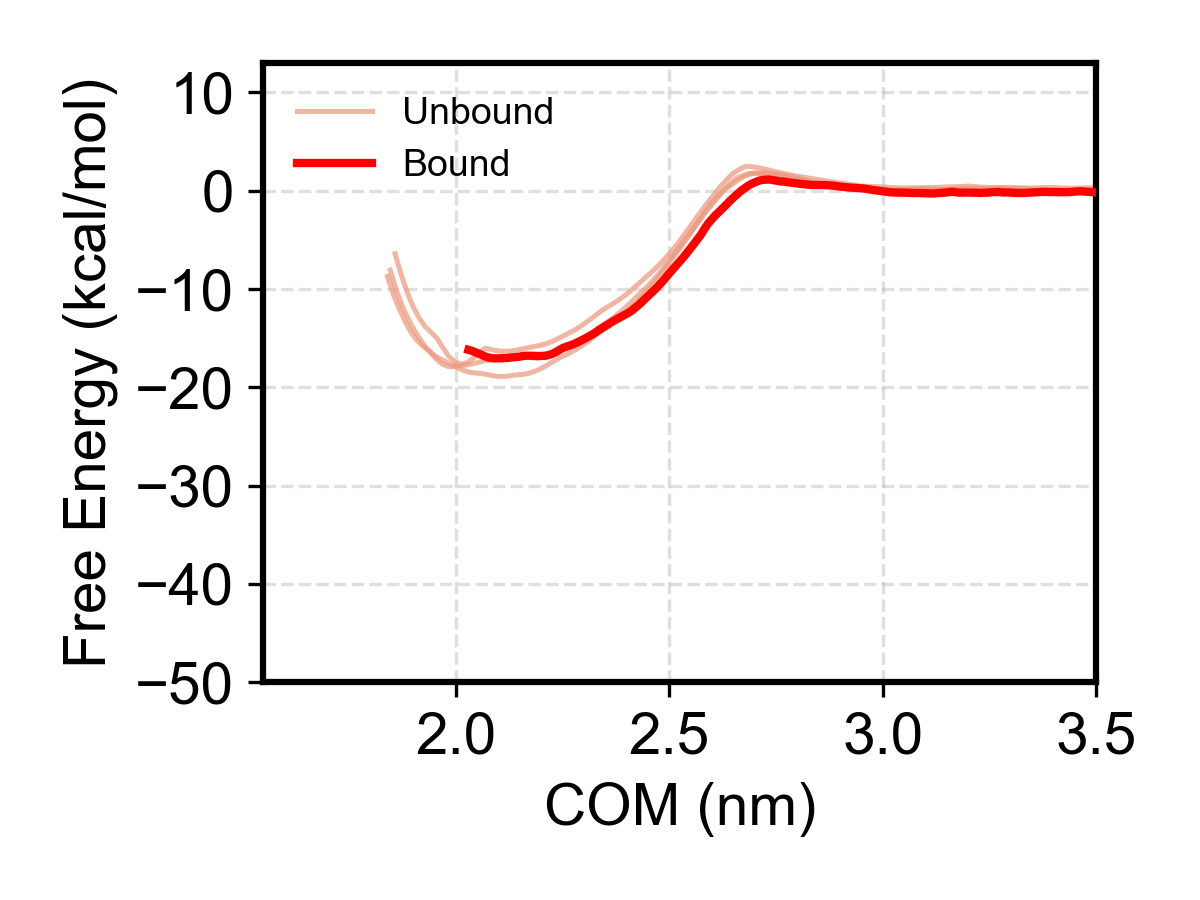

Supplement: Supplementary file 2 [file nn5c18643_si_002.zip › supplementary-files/pmf/reported-equilibration/gnp/plot-pmf/pmf-hb-nh3s3_100ns.png]

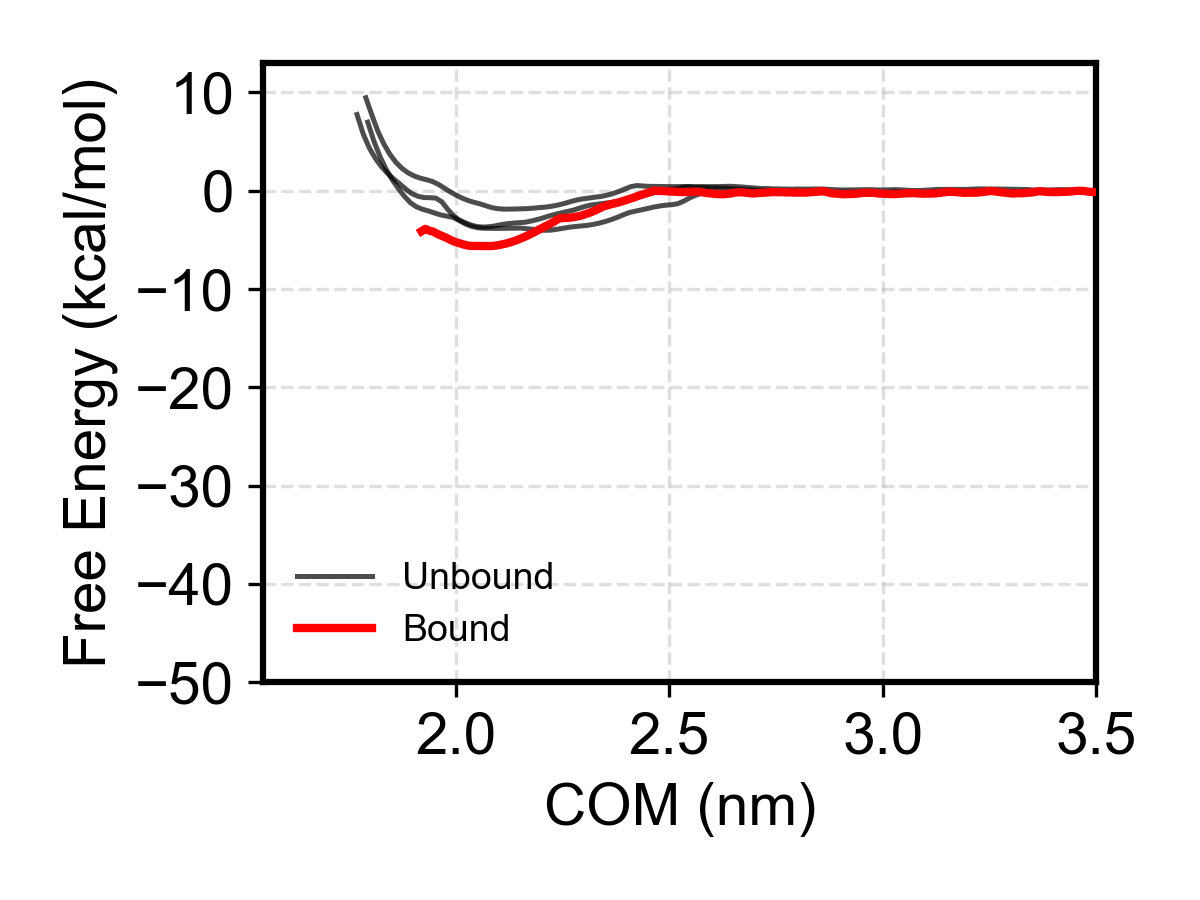

Supplement: Supplementary file 2 [file nn5c18643_si_002.zip › supplementary-files/pmf/reported-equilibration/gnp/plot-pmf/pmf-hb-oh_sam_100ns.png]

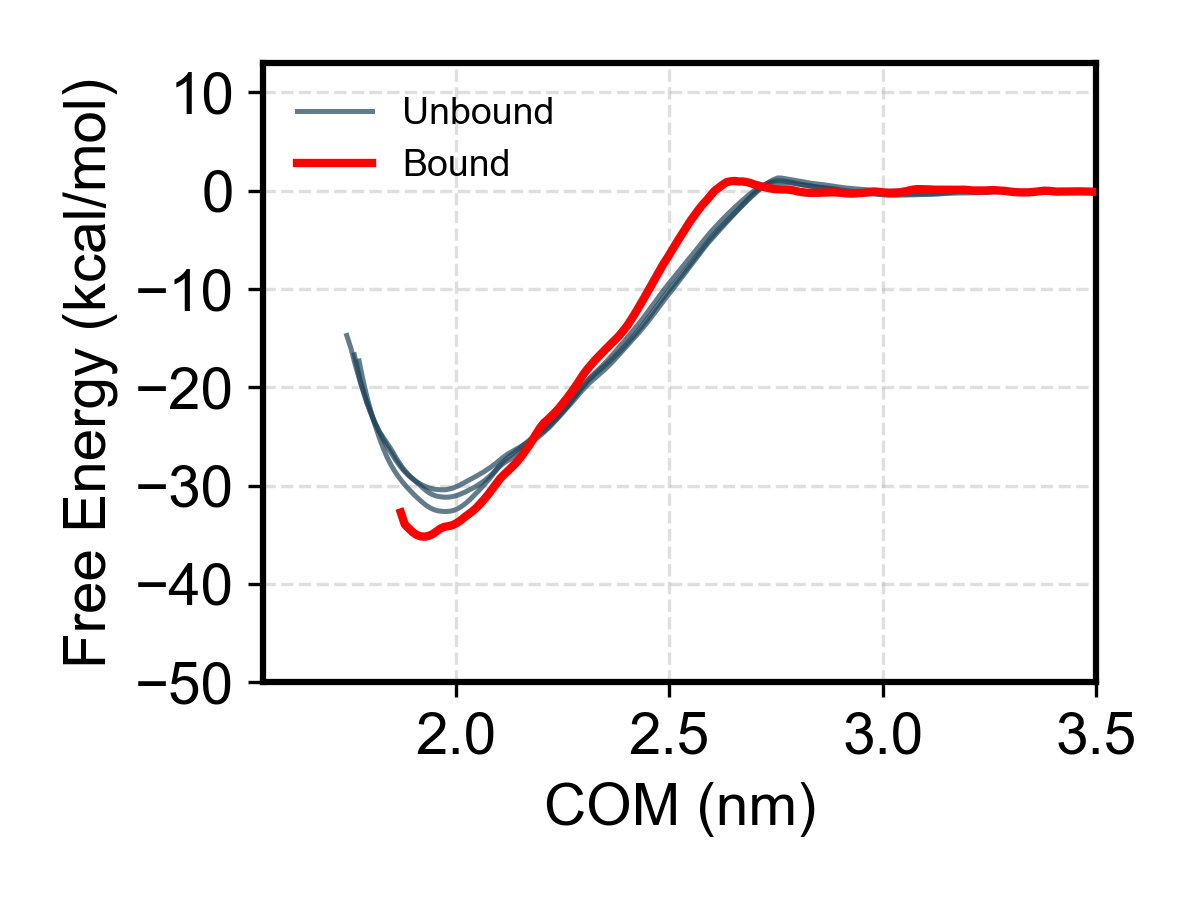

Supplement: Supplementary file 2 [file nn5c18643_si_002.zip › supplementary-files/pmf/reported-equilibration/gnp/plot-pmf/pmf-hb-ohs0_100ns.png]

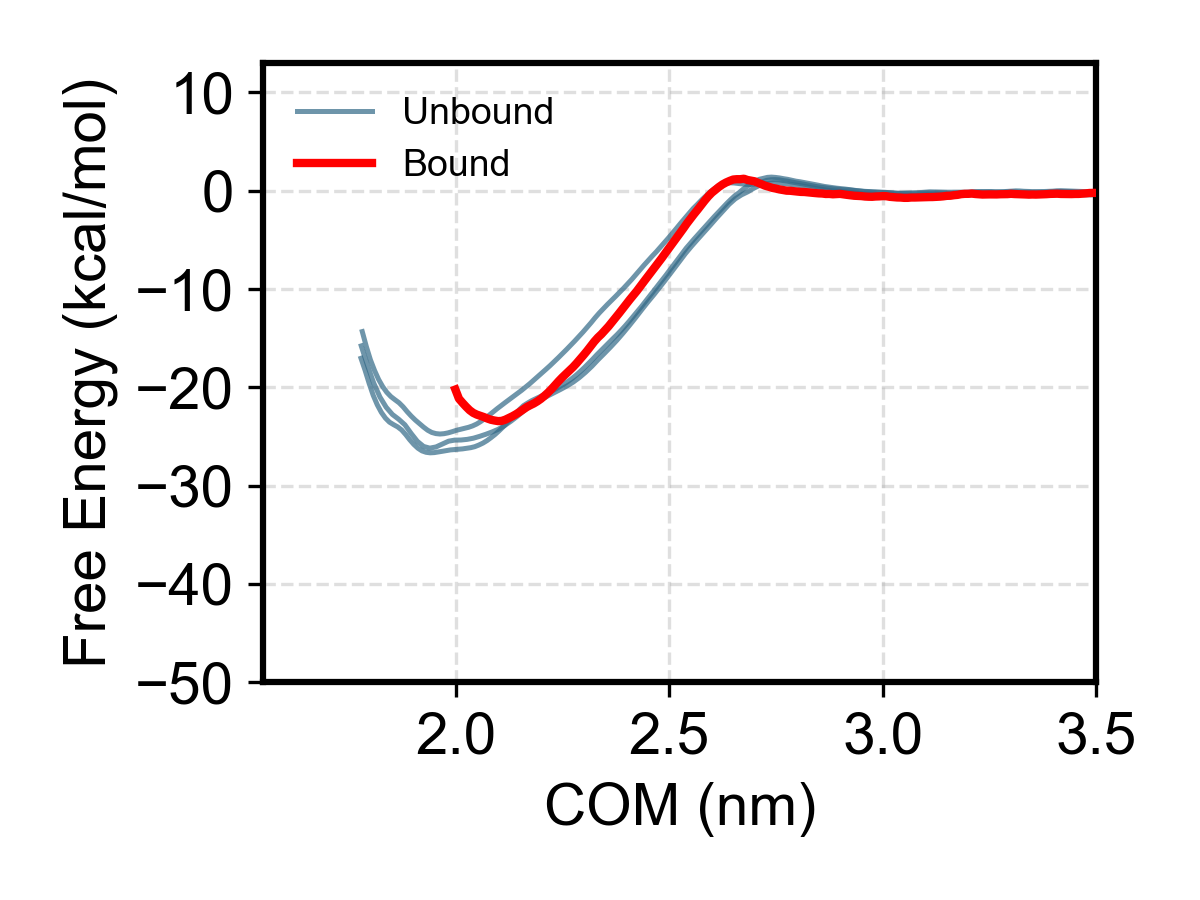

Supplement: Supplementary file 2 [file nn5c18643_si_002.zip › supplementary-files/pmf/reported-equilibration/gnp/plot-pmf/pmf-hb-ohs1_100ns.png]

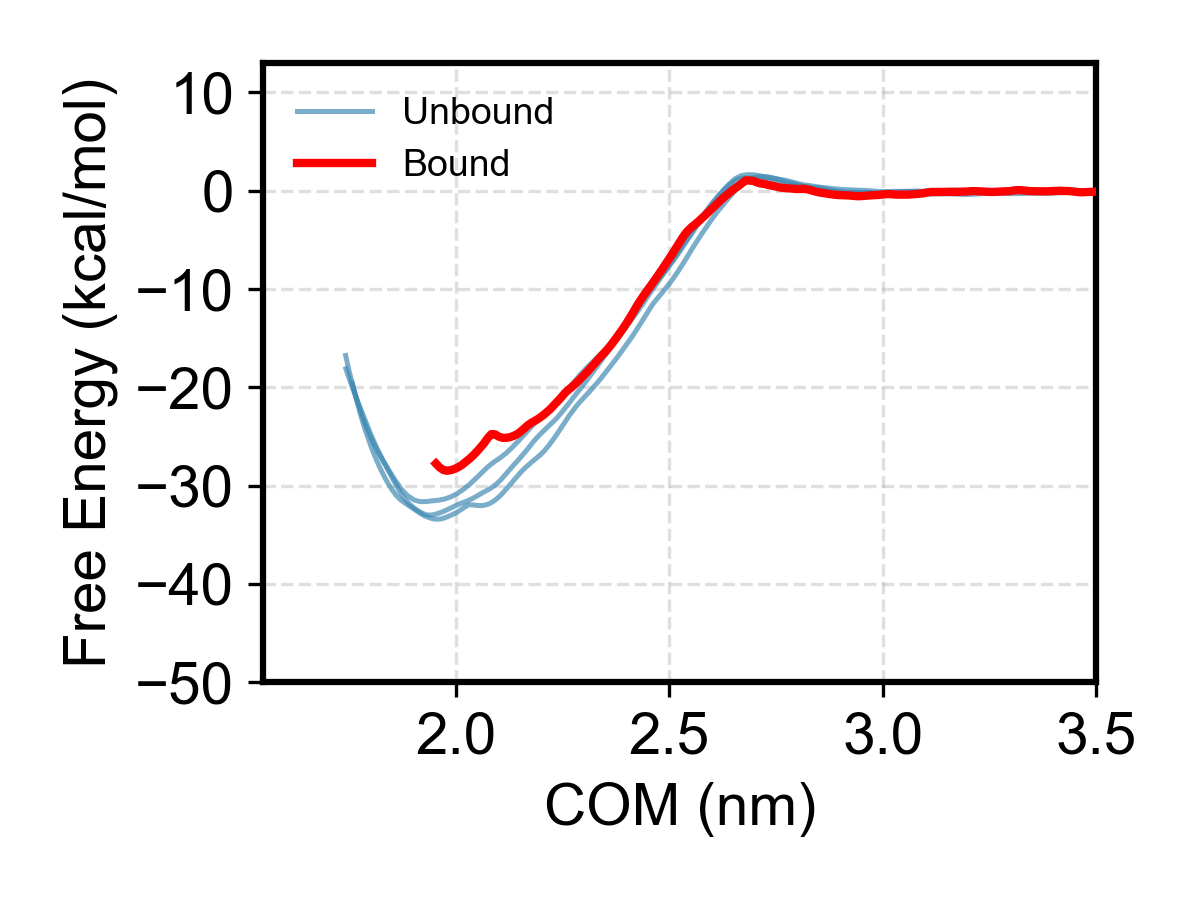

Supplement: Supplementary file 2 [file nn5c18643_si_002.zip › supplementary-files/pmf/reported-equilibration/gnp/plot-pmf/pmf-hb-ohs2_100ns.png]

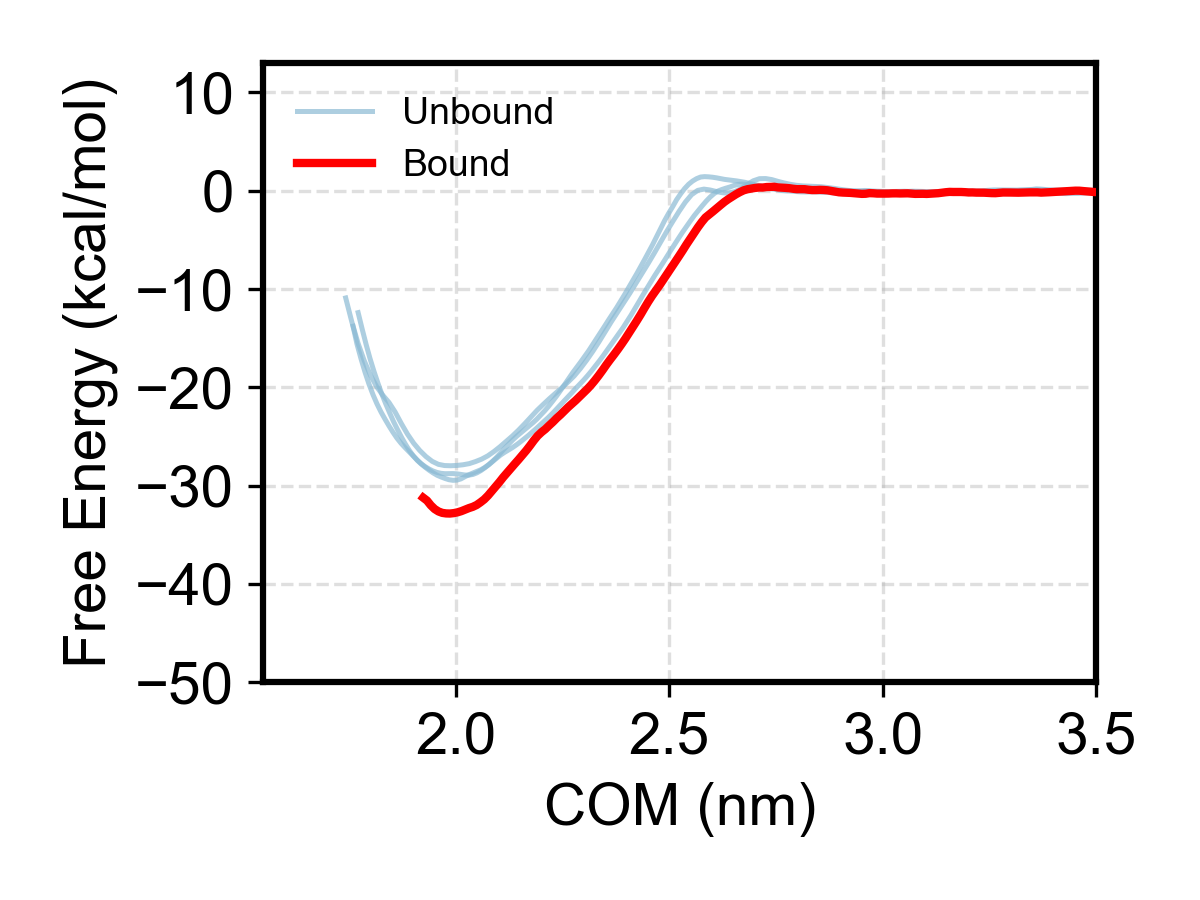

Supplement: Supplementary file 2 [file nn5c18643_si_002.zip › supplementary-files/pmf/reported-equilibration/gnp/plot-pmf/pmf-hb-ohs3_100ns.png]
